# High Levels of Genetic Diversity within Nilo-Saharan Populations: Implications for Human Adaptation

**DOI:** 10.1016/j.ajhg.2020.07.007

**Published:** 2020-08-10

**Authors:** Julius Mulindwa, Harry Noyes, Hamidou Ilboudo, Luca Pagani, Oscar Nyangiri, Magambo Phillip Kimuda, Bernardin Ahouty, Olivier Fataki Asina, Elvis Ofon, Kelita Kamoto, Justin Windingoudi Kabore, Mathurin Koffi, Dieudonne Mumba Ngoyi, Gustave Simo, John Chisi, Issa Sidibe, John Enyaru, Martin Simuunza, Pius Alibu, Vincent Jamonneau, Mamadou Camara, Andy Tait, Neil Hall, Bruno Bucheton, Annette MacLeod, Christiane Hertz-Fowler, Enock Matovu, the TrypanoGEN Research Group of the H3Africa Consortium, Enock Matovu, Issa Sidibe, Dieuodonne Mumba, Mathurin Koffi, Gustave Simo, John Chisi, Vincent P. Alibu, Annette Macleod, Bruno Bucheton, Christianne Hertzfowler, Alison Elliot, Mamadou Camara, Ozlem Bishop, Julius Mulindwa, Oscar Nyangiri, Magambo Phillip Kimuda, Elvis Ofon, Bernadin Ahouty, Justin Kabore

**Affiliations:** 1 College of Veterinary Medicine, Animal Resources and Biosecurity, Makerere University, P.O. Box 7062, Kampala, Uganda; 2 College of Natural Sciences, Makerere University, P.O. Box 7062, Kampala, Uganda; 3 Centre for Genomic Research, University of Liverpool, Liverpool L69 7ZB, UK; 4 Institut de Recherche en Sciences de la Santeì (IRSS) - Uniteì de Recherche Clinique de Nanoro (URCN), Nanoro, Burkina-Faso; 5 Institute of Genomics, University of Tartu, 51010 Tartu, Estonia; 6 Department of Biology, University of Padova, Via U. Bassi, 58/B - 35121 Padova, Italy; 7 Université Jean Lorougnon Guédé (UJLoG) de Daloa, Côte d’Ivoire; 8 Institut National de Recherche Biomedicale, Avenue de la Democratie, Kinshasa Gombe, P.O. Box 1197 Kinshasa, Democratic Republic of Congo; 9 Faculty of Science, University of Dschang, P.O. Box 67, Dschang, Cameroon; 10 University of Malawi, College of Medicine, Department of Basic Medical Sciences, Private Bag 360, Chichiri, Blantyre 3, Malawi; 11 Institute, Centre International de Recherche-Développement sur l’Elevage en zones Subhumides (CIRDES), 01 BP 454 Bobo-Dioulasso 01, Burkina Faso; 12 Department of Disease Control, School of Veterinary Medicine, University of Zambia, P.O. Box 32379, Lusaka, Zambia; 14 Institut de Recherche pour le Développement (IRD), IRD-CIRAD 177, TA A-17/G, Campus International de Baillarguet, 34398 Montpellier, France; 15 Programme National de Lutte contre la Trypanosomose Humaine Africaine, BP 851, Conakry, Guinée; 16 Wellcome Centre for Integrative Parasitology, Biodiversity Animal Health and Comparative Medicine, Glasgow G61 1QH, UK; 17 Earlham Institute Norwich Research Park Innovation Centre, Colney Ln, Norwich NR4 7UZ, UK

**Keywords:** Nilo-Saharan, population genetic variation, signatures of selection

## Abstract

Africa contains more human genetic variation than any other continent, but the majority of the population-scale analyses of the African peoples have focused on just two of the four major linguistic groups, the Niger-Congo and Afro-Asiatic, leaving the Nilo-Saharan and Khoisan populations under-represented. In order to assess genetic variation and signatures of selection within a Nilo-Saharan population and between the Nilo-Saharan and Niger-Congo and Afro-Asiatic, we sequenced 50 genomes from the Nilo-Saharan Lugbara population of North-West Uganda and 250 genomes from 6 previously unsequenced Niger-Congo populations. We compared these data to data from a further 16 Eurasian and African populations including the Gumuz, another putative Nilo-Saharan population from Ethiopia. Of the 21 million variants identified in the Nilo-Saharan population, 3.57 million (17%) were not represented in dbSNP and included predicted non-synonymous mutations with possible phenotypic effects. We found greater genetic differentiation between the Nilo-Saharan Lugbara and Gumuz populations than between any two Afro-Asiatic or Niger-Congo populations. F3 tests showed that Gumuz contributed a genetic component to most Niger-Congo B populations whereas Lugabara did not. We scanned the genomes of the Lugbara for evidence of selective sweeps. We found selective sweeps at four loci (*SLC24A5*, *SNX13*, *TYRP1*, and *UVRAG*) associated with skin pigmentation, three of which already have been reported to be under selection. These selective sweeps point toward adaptations to the intense UV radiation of the Sahel.

## Introduction

**Figure 1:**
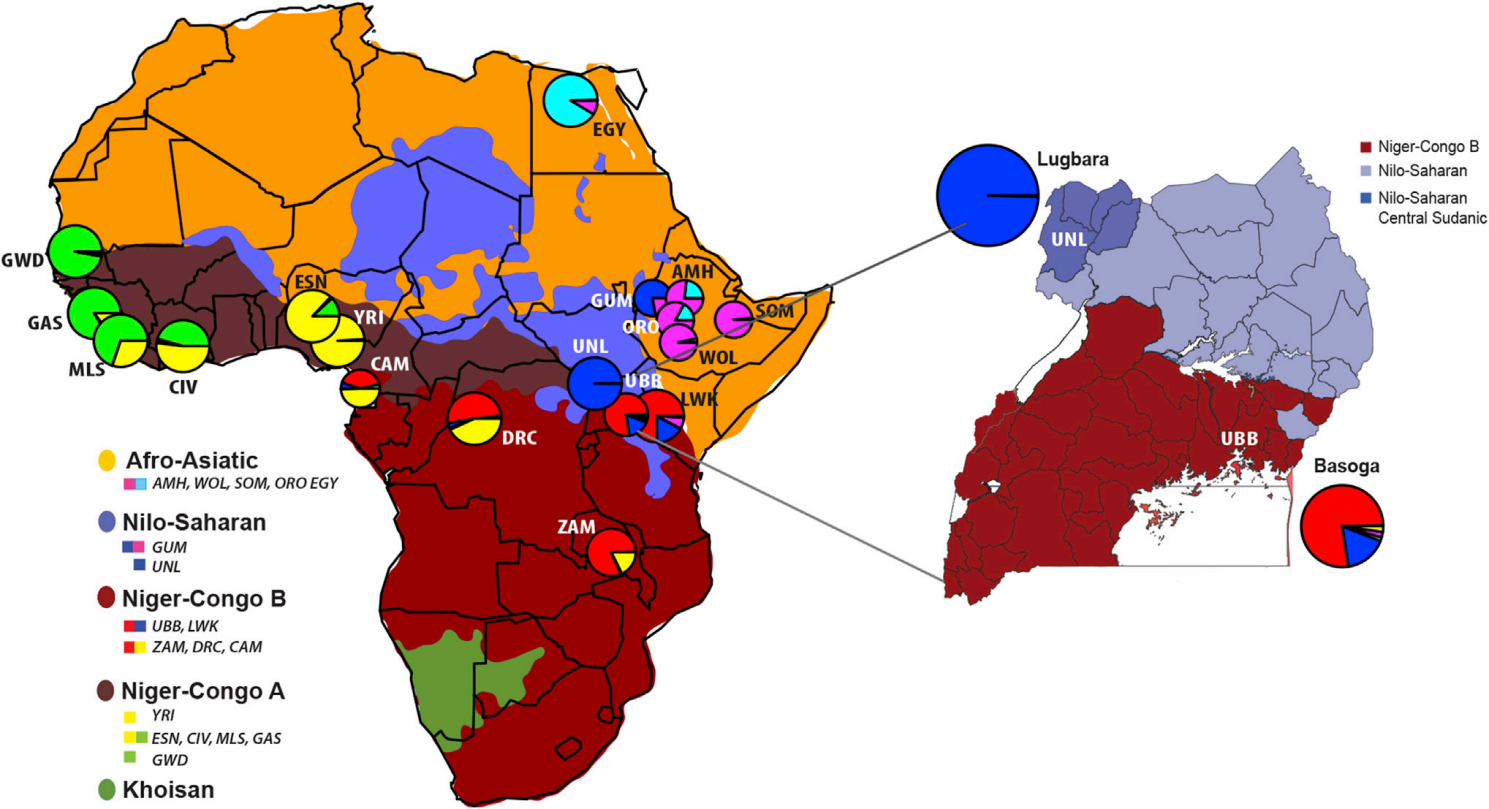
Map of Africa Showing the Distribution of Five Major African Linguistic Families, the Locations Where Samples Were Collected, and the Proportions of Different Genetic Components The pie chart size is proportional to the sample size and pie chart proportions and colors correspond to the proportions and colors of ADMIXTURE components within that population for K = 6 (Figure 3). Note that the map colors for languages are not associated with pie chart colors. The legend shows first the map color for each major linguistic group and second the major colors (>25% admixture component) of the admixture pie charts for each population in that linguistic group. The linguistic distribution map was compiled from data in Ethnologue and used under the Creative Commons Attribution-ShareAlike 4.0 International License. Our populations were sampled from Guinea (GUI), Côte d’Ivoire (CIV), Cameroon (CAM), Democratic Republic of Congo (DRC), Zambia (ZAM), and Uganda (UNL & UBB), the 1000 Genomes project (Gambia [GWD], Sierra-Leone [MSL], Nigeria [ESN, YRI], Kenya [LWK], Egypt [EGY]), and the African Genome Variation project (Ethiopia [AMH, GUM, ORO, SOM, WOL]). The inset map shows sampling sites in Uganda. The Lugbara (UNL) were from West Nile region that is predominantly occupied by Nilo-Saharan speakers and the Basoga (UBB) were from the southern region, which is occupied by Bantu speaking people. This map was overlaid with pie charts derived from the admixture plot using R tools. The Ugandan map was generated using QGIS3.6 (see Web Resources) with regional ethnicity classification traced with inference from “Ethnologue languages of Uganda.”^8^

The modern humans who migrated out of Africa in the last 100 ka came from only a subset of all African populations. The peoples who remained were more genetically diverse and have continued to diversify in response to changing environmental and disease pressures and admixture events.^1^, ^2^, ^3^, ^4^, ^5^, ^6^ African populations have also migrated and intermixed to create the rich mosaic of genetic and cultural variation that is found today.^7^ The paucity of genetic, historical, and archaeological records has led to a heavy dependence on linguistic analysis for classification of African populations, and this strategy has identified four major African language families (Afro-Asiatic, Niger-Congo, Nilo-Saharan, and Khoisan) (Figure 1) and provided evidence for the migration of Bantu speakers out of the Nigeria-Cameroon border region into South and East Africa.^4^ The advent of genetic analysis has generally supported the main population groups identified by linguistic analysis but has also revealed admixture between speakers of different language groups and language acquisitions from genetically unrelated groups.^4^^,^^6^^,^^9^

The Nilo-Saharan family comprises 206 languages spoken by 34 million people (1996 estimate) and is divided into approximately 12 subgroups.^10^^,^^11^ This family is particularly problematic for linguists because there is only weak evidence for establishing the relationships between the subgroups and some authors treat Nilo-Saharan as a collection of isolated language groups rather than a single family.^11^ Some smaller Nilo-Saharan groups (Gumuz, Koman, Kadu, Chabu) have been excluded from the Nilo-Saharan family by some authors or treated as early branching distantly related groups by others.^10^^,^^12^ Genetic data can be used to show how linguistic groups map onto genetically defined human populations.^4^ However, genomes have been sequenced from fewer than 100 of the 2,139 African linguistic groups recognized by Ethnologue.^6^^,^^13^, ^14^, ^15^, ^16^ Here we have sequenced the genomes of 50 individuals from the Nilo-Saharan Lugbara population of Northwestern Uganda. The Gumuz is the only other Nilo-Saharan population to be sequenced at this scale and the linguistic evidence for its inclusion in the Nilo-Saharan family is debated.^10^^,^^12^ For comparison we also sequenced the genomes of 250 individuals from 6 new Niger-Congo populations from Guinea, Côte d’Ivoire, Cameroon, Democratic Republic of Congo, Zambia, and Uganda and also included published data from 13 additional African populations from the 1000 Genomes and African Genome Variation Projects.^2^^,^^17^ We show that the Lugbara are genetically distinct from all Niger-Congo and Afro-Asiatic populations and from the Gumuz.^2^^,^^5^ Through this level of sequencing, we have been able to use the major methods for identification of loci under selection, iHS and xpEHH, which require at least 15 genomes to achieve 80% power.^18^ To date, this number of samples has only been sequenced from 7 Niger-Congo, 6 Afro-Asiatic, and a single putative Nilo-Saharan population (Gumuz).^2^^,^^16^^,^^19^ Analyses of Niger-Congo genomes have already identified loci associated with resistance to malaria and human african trypanosomiasis (HAT).^20^^,^^21^ In the Lugbara we found loci under selection associated with skin pigmentation and hair formation.

## Subjects and Methods

### Study Samples

The samples used for this study were obtained from the TrypanoGEN biobank,^22^ the numbers and ethnic groups of the samples from each country are shown in Table S1. Groups of samples that cluster together on the MDS plot and appear similar on the Admixture plots are referred to by the name of the linguistic group unless there were multiple linguistic groups within a cluster, in which case they are referred to by the country name or abbreviation (Table S1). Ethical approval for the study was provided by the ethics committees of each TrypanoGEN consortium member: Uganda (Vector Control Division Research Ethics Committee (Ministry of Health), Uganda National Council for Science and TechnologyHS 1344), Zambia (The University of Zambia Biomedical Research Ethics Committee: 011-09-13), Democratic Republic of Congo (Minister de la Sante Publique: No 1/2013), Cameroon (Le Comité National d’Ethique de la Recherche pour la Sante Humain: 2013/364/L/CNERSH/SP), Côte d’Ivoıre (Ministere de la Sante et de la Lutte Contre le SIDA, Comité National D’Ethique et de la Recherche 2014/No 38/MSLS/CNER-dkn), and Guinea (Comité Consultatif de Déontologie et d’Éthique [CCDE] de l’Institut de Recherche pour le Développement: 1-22/04/2013). All the participants in the study were guided through the consent forms, and written consent was obtained to collect biological specimens. Study participants provided informed consent for sharing and publishing their anonymized data.

Peripheral blood was collected from the participants at the field sites, frozen, and transported to reference laboratories. DNA was extracted using the whole blood MidiKit (QIAGEN). The DNA was quantified using the Qubit (QIAGEN) and approximately 1 μg was used for sequencing at the University of Liverpool, UK. DNA from Cameroon and Zambia was sequenced at Baylor College, USA.

### Sequencing and SNP calling

300 participants’ DNA samples (Lugbara [UNL], 50; Basoga [UBB], 33; Zambia [ZAM], 41; Democratic Republic of Congo [DRC], 50; Cameroon [CAM], 26; Côte d’Ivoire [CIV], 50; Guinea [GAS], 50) were selected and subjected to whole-genome sequencing (Table S1). The whole-genome sequencing libraries of samples from Guinea, Côte d’Ivoire, Uganda, and DRC were prepared using the Illumina Truseq PCR-free kit and sequenced on the Illumina Hiseq2500 to 10× coverage at the Centre for Genomic Research (University of Liverpool). The samples from Zambia and Cameroon were sequenced on an Illumina X Ten system to 30× at the Baylor College of Medicine Human Genome Sequencing Centre. The sequenced reads were mapped onto the human_g1k_v37_decoy reference genome using BWA.^23^ The SNP calling on all the samples was carried out using the genome analysis tool kit GATK v3.4^24^ to create a GVCF file for each individual. GVCF files were then merged to create a combined VCF file also using GATK. SnpEff was used for variant annotation.^24^ An analysis of copy number variation has been published separately.^25^

From the 1000 Genomes project^16^ we obtained variant call files of 50 samples from each of the Esan and Yoruba from Nigeria; Mende from Sierra Leone; Gambian from Western Division of The Gambia; Luhya from Western Kenya; five samples from each of five populations of West Eurasian origin: Utah residents with northern and western European ancestry, Finnish from Finland, British in England and Scotland, Iberian from Spain, Toscani from Italy.

From the African Genome Variation Project^2^^,^^26^ we extracted 50 Egyptian genome sequences and 24 from each of the following Ethiopian populations: Amhara, Ethiopian Somali, Oromo, Wolayta, and Gumuz. The African Genome Variation datasets were obtained from European Genome-Phenome Archive,^27^ EGA: EGAD00001000598, EGA: EGAD00001003296, EGA: EGAD00010001221, under the terms of the Wellcome Sanger Institute (WSI) data access agreement.

### Data Quality Control and Filtering

The data were filtered to minimize batch effects potentially introduced by the presence of samples sequenced at different depths by different labs. For descriptive statistics of the TrypanoGEN dataset all loci were retained. For all other analyses, sites that met any of the following criteria were removed; missing data > 10%, loci with < 3 SNP calls, minor allele frequency (MAF) < 0.01, Hardy-Weinberg equilibrium p < 0.001. For population analyses, the remaining SNP loci were thinned in order to retain only loci with *r*^*2*^ < 0.1. Individuals with >10% missing data were also removed. Data were phased with Shapeit2 v2.r837,^28^ which also imputed missing data, prior to combining our data with genomes from the 1000 genomes and African Genome Variation projects using BCFtools (v.1.6),^27^ retaining only loci that were present in all datasets.

For signatures of selection, the filtered and phased variant call format files were further filtered using VCFtools v.0.1.16^29^ to remove loci with MAF < 0.05.

### Multidimensional Scaling Analysis

To infer the population structure based on the underlying genetic variation among the populations, we carried out multidimensional scaling (MDS) using PLINK 1.9^30^ and plotted MDS coordinates using R v.3.2.1.^31^ The MDS was carried out on our sequence data, which was merged with a maximum of 50 samples from each of the 13 additional populations from Africa and Europe from the 1000 Genomes project^16^ and the African Genome Variation project.^2^^,^^26^

### Population Admixture

Admixture was tested for 1 to 9 genetic components (K) using ADMIXTURE 1.23^32^ with 3 replicate runs for each value of K.

All plausible pairs of available populations that might be sources of the selected East African Populations (UNL, UBB, LWK, GUM, AMH) were tested for evidence of contribution to those populations using the F3 test in AdmixTools^33^ and implemented in R using *admixr*.^34^

### Allele Frequency Statistics: In-breeding Coefficient, Tajima D, F_ST_

We followed the workflow of Cadzow et al. for allele frequency statistics.^35^ To determine the extent of inbreeding within each of our populations, we measured the inbreeding coefficient, F,^36^ using VCFtools (v.0.01.14).^29^ The Tajima D statistic^37^ was used to identify regions that did not fit the neutral model of genetic drift and mutation in bins of 3 kb also in VCFtools. The level of population differentiation was estimated with Wright’s F_ST_^38^ in PLINK v.1.9. The pairwise F_ST_ matrix was generated between our sequence data, 1000 Genome project,^16^ and the African Genome Variation Project populations.^2^^,^^26^

### Signatures of Selection

The sequence data were scanned for regions that might be under selection using the Extended Haplotype Homozygosity (EHH) test within and between populations.^39^ The SNP were phased using SHAPEIT v.2.2,^28^ and the R software package *rehh3*^40^ was used to calculate two EHH derived statistics: the intra-population integrated Haplotype Score (iHS)^41^ and inter-population xpEHH score,^42^ that identify SNPs that are under selection in one population but not in another. Only SNPs with a MAF > 0.05 were included in the analysis. We used the method of Voight et al. to identify the regions of the genome under the strongest selection pressure;^41^ the genome was divided into 100 kb bins and the fraction of SNP with iHS > 2 in each bin was obtained. Bins with <20 SNP were disregarded. The 1% of bins with the highest fraction of SNP with absolute iHS > 2 were considered to be significant.^41^ Bins were annotated with the lists of genes that they contained using Biomart. Different types of evidence for signatures of selection were combined using Bedtools v.2.26.0^43^ to identify the intersection of the iHS, with xpEHH and the allele frequency-based statistics of F_ST_ and Tajima D.

## Results

We sequenced the genomes of 50 individuals from the Nilo-Saharan Lugbara population and 250 from 17 linguistic groups from Guinea, Côte d’Ivoire, Cameroon, Democratic Republic of Congo, Uganda, and Zambia (Tables S1 and S2).

The samples from Zambia and Cameroon were sequenced to 30× coverage while other populations were sequenced to 10× coverage. The call rate was 97.4% in the 10× samples and 99.4% in the 30× samples. The 30×-sequenced samples had higher proportions of heterozygotes (9.3%) compared with the 10× sequenced samples (7.5%) and there was a concomitant higher frequency of low Hardy-Weinberg p values in the 10× data (Figure S1). There were 38,963,563 raw variants, filtering removed fourteen individuals and 23,017,723 loci leaving 286 samples and 15,945,844 variant loci that were available for population and signatures of selection analyses. Table S3 shows the number of loci removed by each filtering step, most variants were removed from the analysis because of low count or frequency of minor alleles (21,604,569 MAF < 1% or minor allele count ≤ 2). The mean call rate after filtering was 99.2% for the 10× samples and 99.95% for the 30× samples. The data were phased with Shapeit2, which imputed genotypes at the small number of remaining missing loci. The commonest form of bias in low-coverage data is an excess of singleton variant loci^44^ and these were removed by the filtering strategy (Figure S1).

### The Nilo-Saharan Lugbara Population Has a High Proportion of Novel Variation

We observed little evidence of inbreeding within the populations; the majority of the individuals had an inbreeding coefficient (F) of less than 0.1 (Figure S2). We classified variants as known if they were present in dbSNP build 150 (20/11/2019) and novel if not. We identified approximately 22 million variant loci in the Lugbara population (Table S4, Figure S3). The frequencies of known and novel variants were similar in all the six Niger-Congo populations (12.9% novel, SE 0.003); however, the Nilo-Saharan Lugbara population from North West Uganda had significantly more novel SNPs (17.1% p < 0.001) (Figure S3C), presumably due to an under-representation of Nilo-Saharan populations in previous genomic studies. We assessed the impacts of the variants on function using snpEff; 99% of SNP were classified as “modifier,” and these were mainly intergenic; the remaining 1% of SNPs had more informative classifications: low, moderate, or high impact (Table S4, Figures S3B and S3C). Of the 1% of SNP with informative classifications (low, moderate, or high impact), nearly 90% were predicted to have moderate impact in both known and novel variants. The frequency of high-impact variants was twice as high in the novel variants as it was among the known variants (6.3% *cf.* 3.0%). There was a larger proportion of rare alleles (MAF < 5%) in the set of novel SNPs than in the known SNPs (Figure S4), as expected for SNPs that are unique to a specific population or geographic region.

### The Nilo-Saharan Lugbara Population Is Distinct from Other African Populations

Bi-allelic loci from the 286 TrypanoGEN samples were merged with 1,000 Genomes and African Genome Variation Project data to obtain 10,857,449 loci that were present in all three datasets for population analysis. These were filtered to remove linked loci (*r*^2^ > 0.1) yielding a final dataset of 1,465,578 SNP and 731 samples that were used for MDS, Admixture, and F3 analysis.

**Figure 2:**
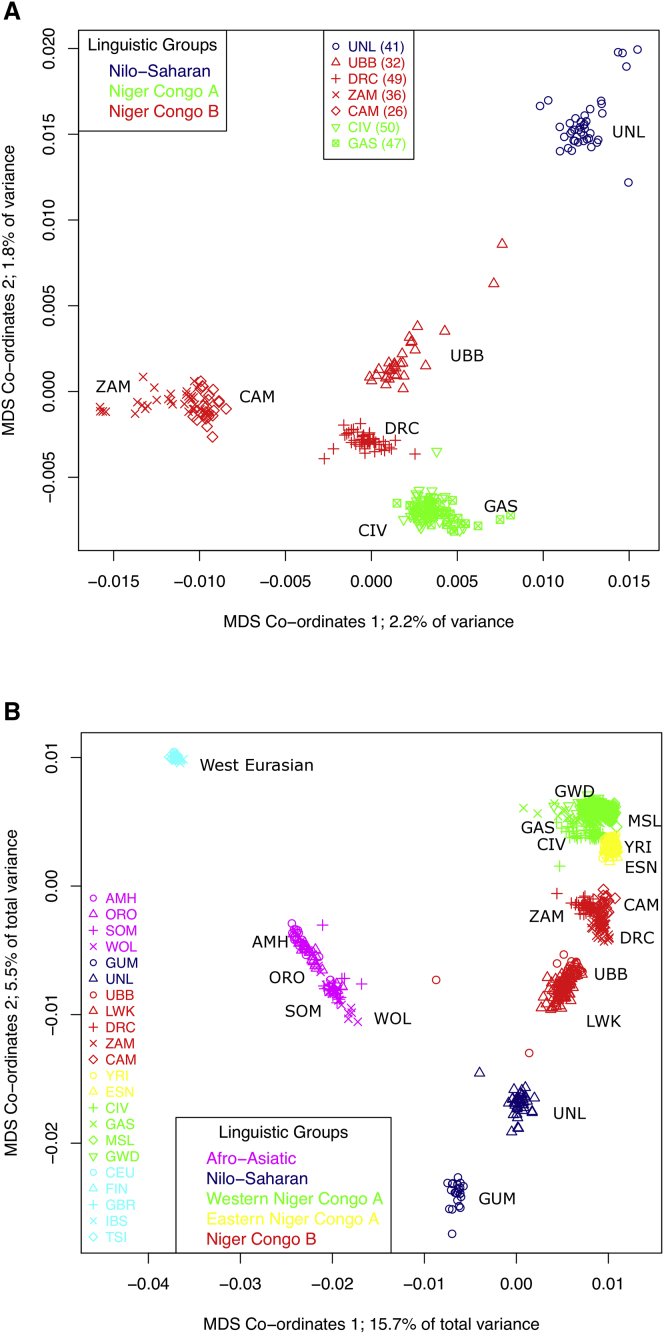
Multidimensional Scaling Analysis of Sequenced Populations (A) This study: Guinea (GAS), Côte d’Ivoire (CIV), Cameroon (CAM), Democratic Republic of Congo (DRC), Uganda (Nilotics, UNL, Niger Congo B, UBB), and Zambia (ZAM); seven Soli/Chikunda (Niger-Congo B)-speaking individuals were outliers by MDS and are not shown in this plot but are shown in Figure S5A. (B) This study and African Genome Variation Project Ethiopian samples Amhara (AMH), Welayta (WOL), Oromo (ORO), Ethiopian Somali (SOM), and Gumuz (GUM) and 50 samples from each 1000 Genomes African population Nigeria (ESN, YRI), Gambia (GWD), Mende Sierra Leone (MSL), Kenya (LWK). Colors for each cluster are taken from the color for the dominant genetic component for that cluster in the admixture plot at K = 6.

Multidimensional scaling analysis (Figure 2) showed that samples formed tight geographic groups irrespective of data source or sequence coverage. The exception was the Nilo-Saharan Lugbara population from North West Uganda, which was distinct from both the Nilo-Saharan Gumuz of Ethiopia and the Basoga from southeast Uganda. The two Nilo-Saharan populations were well separated from each other and from the East African Niger-Congo B and the Ethiopian Afro-Asiatic populations. Even when combined with a West Eurasian dataset (Figure S5B), the two putative Nilo-Saharan populations (Lugbara and Gumuz) appeared as divergent from each other as Niger-Congo-A and Niger-Congo-B populations from East and West Africa. This demonstrates that the focus on genetics of Niger-Congo and Afro-Asiatic populations has led to the neglect of the greater diversity within other African populations.

### The Nilo-Saharan Lugbara Show Low Genetic Admixture and High Genetic Distance from Other African Populations

**Figure 3:**
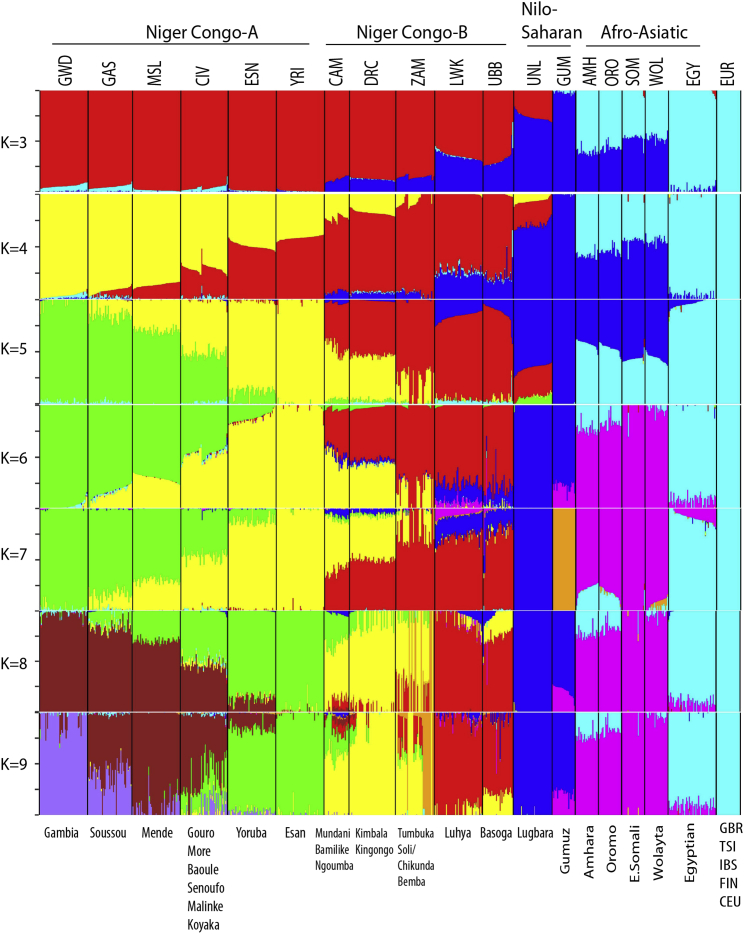
Genetic Admixture and Differentiation in Our Data, Selected 1000 Genomes, and AGVP Populations Admixture plot (731 samples) for K = 3 to K = 9. Genome sequences from this study, 1000 Genomes African samples, AGVP Egyptian, Ethiopian, and European populations (GBR, British from England and Scotland; TSI, Toscani in Italy; IBS, Iberian in Spain; FIN, Finnish in Finland; CEU, Utah residents with Northern and Western European ancestry). Three replicates were carried out for each value of K.

We then used Admixture to analyze the population structure of the same 731 samples used for the MDS analysis. The admixture coefficients of variation were very similar (0.262–0.271) for all numbers of genetic components (K3-9) (Figure S6). Although caution should be used when interpreting Admixture clusters as broad genetic components,^45^ remarkably at all values of K except K = 7 Gumuz and Lugbara shared a single large component, which was also important in Afro-Asiatic samples (at K ≤ 5) and to a lesser extent in East African Niger Congo B samples (LWK, UBB) (Figure 3).

With K > 5 the Niger-Congo populations separated into an east African cluster of the Ugandan Basoga and Kenyan Luhya, a central African cluster of the Zambia, Cameroon, and Democratic Republic of Congo, a Nigerian cluster of the Esan and Yoruba, and a far west-African cluster of the Côte d’Ivoire, Sierra Leone, Guinea, and Gambia populations. We also observed at K ≥ 8 a homogeneous group of seven Soli/Chikunda (Niger-Congo B)-speaking individuals within the Zambia population with no admixture with other populations and who were also outliers on the MDS coordinates plot (Figure S5A), the source of this divergent ancestry is unknown.

### F3 Tests of Admixture Hypotheses

**Figure 4:**
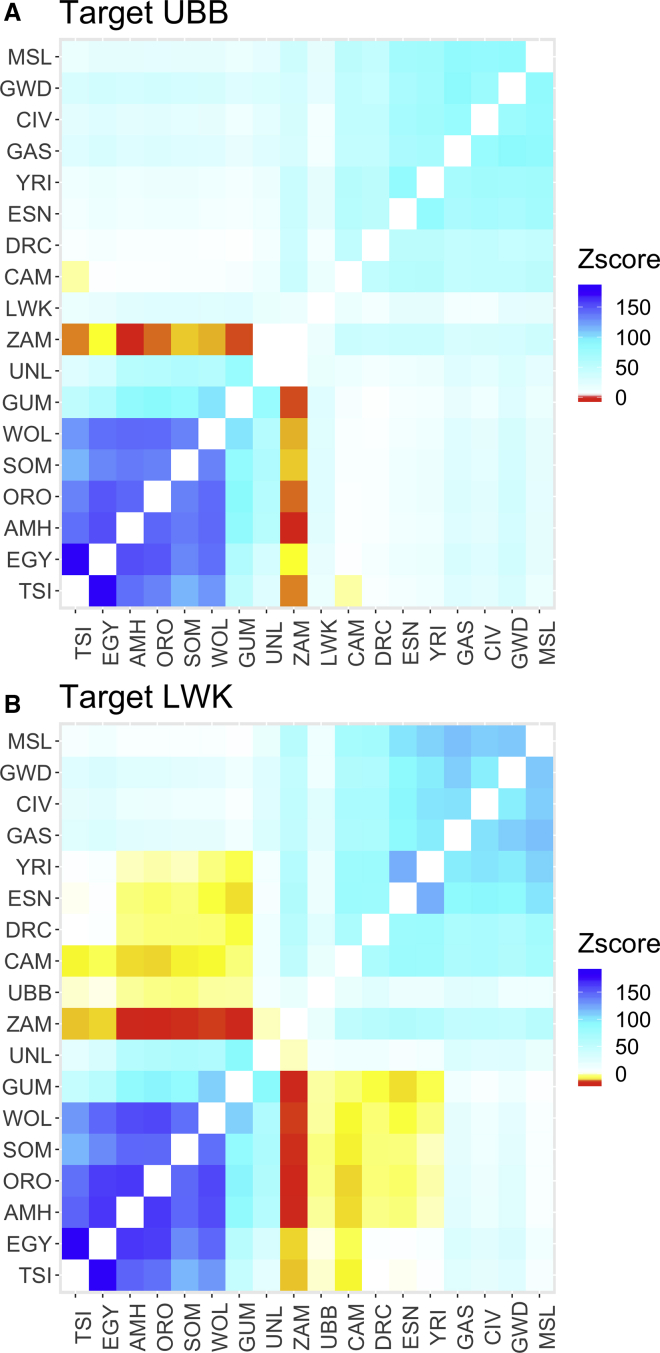
F3 Tests of Admixture (A) Target UBB; Z scores for probability that a pair of populations contributed ancestry to the Uganda Niger Congo B Basoga. (B) Target LWK; Z scores for probability that a pair of populations contributed ancestry to Kenyan Luhya. Heatmap color represents intensity of Z score for probability that a population contributes genetic components to the target. Negative Z scores (yellow to red) are associated with increasingly strong evidence of a contribution and positive scores (cyan to blue) are associated with increasingly strong evidence against a contribution. White squares are inconclusive.

The admixture hypotheses generated by Admixture were tested with the three populations (F3) test implemented with AdmixTools.^33^ All possible pairs of 2 West Eurasian (TSI, EGY) and 17 African populations (AMH, ORO, SOM, WOL, DRC, CAM, ZAM, ESN, YRI, GWD, MSL, GUI, CIV, LWK, UBB, GUM, UNL) were tested as possible sources of five East African populations (Afro-Asiatic AMH; Nilo-Saharan GUM and UNL; East African Niger-Congo B UBB and LWK) (Figures 4 and S8).

Pairs of each African population and each West Eurasian population were plausible sources to the Amhara (AMH) population consistent with the Admixture plot which suggests that the Afro-Asiatic populations have a large West Eurasian admixture component as previously reported (Figure S8).

No pairs of populations were jointly source to either of the Nilo-Saharan populations (UNL and GUM) (Figure S8). However, the Gumuz and Lugbara had very different contributions to the ancestry of the Kenyan Luhya (Figure 4), despite sharing apparently similar ancestral components in the Admixture plot (Figure 3). There was evidence that both the Gumuz and Afro-Asiatic populations were plausible sources to the Luhya when paired with most African populations (Zscore < −16 for pairings with Zambia). In contrast there was very little evidence of ancestry from the Lugbara, which were only compatible with the Zambian population as plausible admixture sources, and even there the signal was much weaker (Z score = −2.7). The Gumuz but not the Lugbara also contributed to the Ugandan Basoga ancestry (Figure 4) but only when paired with the Zambian population.

These observations are most consistent with the population structure indicated in the Admixture plot at K = 6. At K = 6 the dominant ancestry component in Lugbara and Gumuz (dark blue in Figure 3) is also shared with the Luhya and Basoga, but this is not consistent with the F3 data. However, a minor component of the Gumuz (pink at K = 6), which is not observed in the Lugbara, is also shared with Luhya and Basoga and this is consistent with F3 data, which shows a Gumuz but not Lugbara contribution to these populations. The pink perhaps represents a pre-Bantu expansion East African population that has contributed to the Gumuz, Luhya and Basoga genomes but not the Lugbara.

We obtained pairwise F_ST_ distances between the Ugandan Lugbara and the other African populations to determine the genetic distance between them (Table S5, Figure S7). F_ST_ was relatively high (mean F_ST_ > 0.015) between the Nilo-Saharan Lugbara samples and the Niger-Congo populations, except for the Uganda Basoga population (mean F_ST_ = 0.011) and Kenyan Luhya population (mean F_ST_ = 0.012). The Lugbara and Gumuz populations are about 1,000 km apart compared with the approximately 4,000 km, which separates the West and East African Niger-Congo A and B populations. However, F_ST_ between Niger-Congo A and B (0.008) was lower than between Lugbara and Gumuz (F_ST_ = 0.025, Table S5), indicating that Lugbara and Gumuz populations have very different histories.

### Signatures of Selection in Nilo-Saharan Lugbara

Given the relative genetic isolation of the Nilo-Saharan Lugbara, we hypothesized that they could have unique genetic adaptations to their environment. We sought to identify those regions of the genomes that were under selection, using the linkage disequilibrium-based models of extended haplotype homozygosity (EHH). Those alleles with extreme EHH were then validated using the allele frequency-based F_ST_ statistic and Tajima’s D. Of the 15,945,844 variant loci that passed QC, only those with MAF > 5% were retained for these analyses, a total of 8,882,525 in the Lugbara and 9,107,514 in the Basoga.

### Signatures of Selection in the Lugbara and Basoga Populations

**Figure 5:**
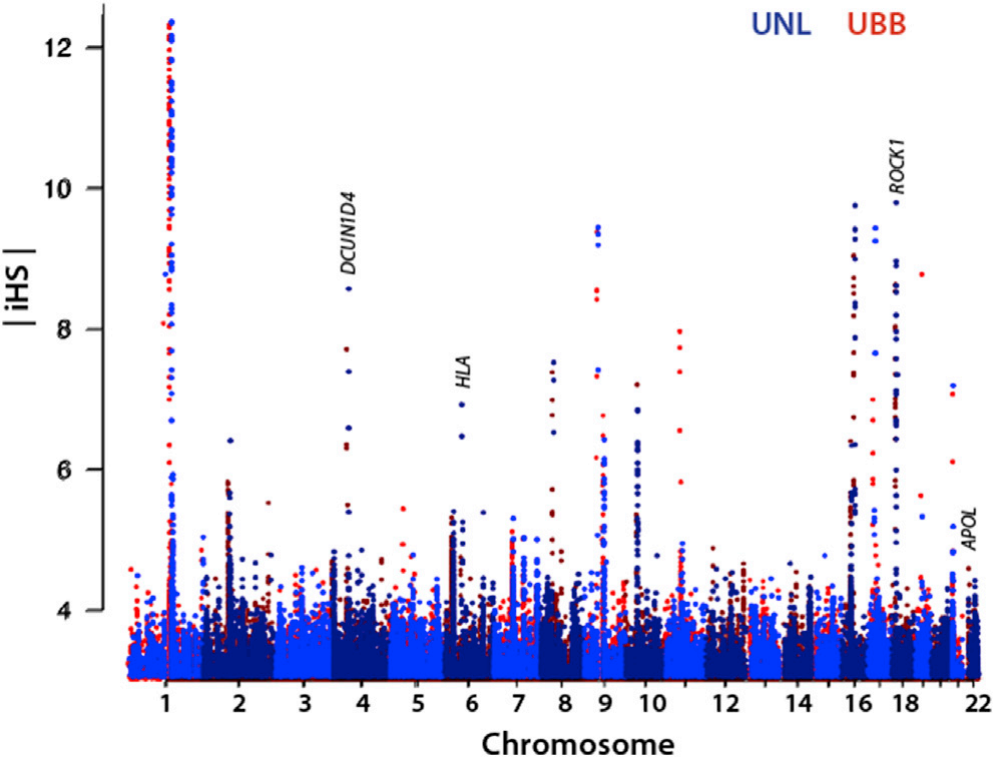
Genome-wide Signatures of Selection in the Lugbara and Basoga Manhattan plot showing SNPs with extreme absolute iHS values (|iHS| > 3.0) that occur in the Lugbara (UNL blue) and Basoga (UBB red) populations.

We compared the regions under selection within the Lugbara and Basoga populations. The Basoga population was selected due to their geographic proximity to the Lugbara (500 km) (Figure 1), the minimally shared genetic ancestry between these two Ugandan populations (Figure 3), and because the Ugandan Basoga can act as representatives of Niger-Congo B populations. Using the phased haplotype dataset of the Lugbara and Basoga populations, the EHH derived integrated haplotype score (iHS) values were calculated using the *rehh3* software for which we observed a normal distribution of the absolute iHS values (Figure S9). The Manhattan plot (Figure 5) shows 12 regions with extreme iHS (|iHS| > 6). However, there were protein-coding genes within 100 kb of only two of these peaks (*ROCK1*, *DCUN1D4*). Both genes are involved in diverse ranges of intracellular activities making it difficult to predict a specific effect on phenotype.^46^^,^^47^ We therefore calculated the frequency of SNP with |iHS| > 2 in 100 kb bins^41^ to identify the regions with greatest evidence of selection and that might contain genes associated with known phenotypes (Table S9). The *HLA* region had some of the highest frequencies of SNP with |iHS| > 2 as well as some of the highest values of iHS (> 6) and has been found to have signatures of selection previously.^48^ A list of genes that are under selection and are also shared between the UNL and UBB populations is shown in Table 1.

**Table 1:** The Top 20% of Protein-Coding Genes with Strongest Signatures of Selection in the Lugbara Population

**Chr**	**Associated Protein-Coding Gene**	**Associated Effect**	**Ref.**
1	*BX842679.1,****LYPD8, SDHC, C1orf192, NBPF20, PRDM2, SLC9A1, FAM46B***, *GFI1*^*a*^*, GPR89A,****PRPF3, ITLN2, F11R***, *NBPF14, DESI2,****PRMT6, FLG***^***b***^***, XCL2***, *CENPL***, *FGGY, PRAMEF10, NR0B2, C1orf172***, *RIMKLA,****PPIAL4G***, *C1orf159,****CD48***	^a^myeloid leukemia, ^b^atopic dermatitis	^49^^,^^50^
2	***IRS1***^***C***^, *RGPD5,****PARD3B***, *PFN4, TP53I3, DYNC1I2,****CH17-132F21.1, C2orf47, SPATS2L, ZNF2***, *ARHGAP15, VPS54,****AC017081.1, RAB3GAP1, MAP3K19,****ST3GAL5,****RFTN2***, *ASXL2, GALNT14,****AMER3, PROKR1***	^c^diabetes	^51^
3	*HACL1,****C3orf67***, *LRRIQ4,****FXR1, TMEM45A, TOP2B***, *ALCAM,****IQCB1, GOLGB1***, *TF*^*d*^*, FAM162A, WDR5B, ABCF3, VWA5B2, RPL24, IQCF3, HTR3E, ACTRT3, FILIP1L, SPSB4, MYNN, COLQ,****ABHD14A-ACY1***, *NEK4, EIF5A2, RPL22L1,****CAMK2N2, PSMD2, KCNH8***, *SFMBT1, TMEM110*	^d^anemia	^52^
4	***ABCG2***, *DCAF4L1, TMEM33, KLHL8, USP46,****ERVMER34-1***, *PAICS,****C4orf33***, *STATH, RXFP1, TECRL, ENPP6, STOX2, ANTXR2,****KLHL2***, *HTN1, HTN3,****SCLT1***, ***EIF4E, NDST3***^***e***^, *C4orf46*	^e^schizophrenia	^53^
5	*NR2F1, PARP8,****TMEM232, PRELID2, JAKMIP2, PJA2, RP11-1026M7.2***, *IL9, SLC25A48,****TIMD4***^***f***^***, FAM153B***, *NNT,****RBM27, PLAC8L1***, *SDHA,****MYO10, TTC1, SKP1***, *MED7, FAM71B, ITK*^g^, *TGFBI*	^f^tuberculosis, ^g^HIV	^54^^,^^55^
6	***SAMD3, TMEM200A, UNC5CL, IPCEF1, OPRM1, EPHA7***, *PKIB, DDO, METTL24,****TULP4***, *ID4,****HLA-DQB1***^**h**^**, *HLA-DQA1***, *BAI3, COX6A1P2, FGD2, SOX4, MYLK4, WRNIP1,****GRIK2***	^h^HIV, ^h^tuberculosis, ^h^diabetes	56, 57, 58
7	*IGF2BP3,****MUC12***, *MUC3A,****NAMPT, AOC1, KCNH2, C7orf62, AC006967.1, RBM48, GATS, PVRIG, GNA12, POM121L12, OR9A2***^**i**^**, *KEL, CARD11***, *TRPV5,****AZGP1,****THSD7A, ZNF680, AGR2,****CDK6***, *SERPINE1, ISPD*	^i^odor perception	^59^
8	***FAM83A, PRR23D1, LRLE1, ZNF696, STC1***, *SFRP1, ADCY8,****CSMD1, SDR16C5***, *ZNF705G,****DDHD2, PPAPDC1B***, *PBK, CLN8,****COPS5***		
9	***AL953854.2, BX255923.1, CR769776.1, TPRN***^**j**^**, *SSNA1, CBWD5, AL591479.1, CBWD7***, *PHF2,****C9orf85, BX649567.1***, *TRMT10B, GRIN1,****BRINP1, RP11-195B21.3***, *AL365202.1,****INPP5E***	^j^deafness	^60^
10	*BLNK,****ZNF37A, FAM21C, AL591684.1, PLEKHS1, CDNF***^**K**^, *SORCS1,****A1CF, ASAH2B, DNAJB12, LARP4B,****MALRD1, BLOC1S2, PKD2L1, ANKRD2, UBTD1,****ADAM12, AFAP1L2, FANK1, KNDC1***, *UTF1,****MTRNR2L7, C10ORF68***	^k^stroke	^61^
11	***SPATA19***, *MRVI1,****DPP3, CTD-3074O7.11, MOGAT2, ANO3, FAM86C1, TREH***, *DDX6, PGAP2, FADS3, AL356215.1,****UBASH3B***, *UVRAG*^l^, ***IFT46***	^l^autophagy	^62^
12	***SDR9C7, GALNT9***^**m**^, *MGAT4C,****NTS, SCYL2***^**n**^, *KCNJ8,****AC073528.1***, *PRPH, TROAP, CLEC6A,****LRIG3, TMTC2***, *HECTD4, SMCO2, AEBP2,****LGR5***, *GAS2L3, CIT, C12orf56, ANO6,****CCDC59***	^m^neuralblastoma ^n^arthrogryposis	^63^^,^^64^
13	***SLC15A1, DOCK9, THSD1, GPC5, HNRNPA1L2, C1QTNF9B, SPRY2, CKAP2***, *RFC3,****RGCC, VWA8***, *DZIP1*		
14	***PPP2R5C***, *DCAF5,****SERPINA6, RP11-796G6.2***, *TEX22,****EGLN3, NPAS3***		
15	***NDNL2, LMAN1L, FAM219B, MPI***, *PGPEP1L, CERS3*^O^, *CKMT1A, CSK*^P^, *CYP1A2,****CORO2B, ITGA11***, *RAB11A,****NEDD4, C2CD4A, FGF7, HDC, C15orf60***, *DUOX2,****CPLX3***, *BLM,****HCN4***	^o^ichthyosis, ^p^SLE	^65^^,^^66^
16	***OTOA***, *METTL22, TMEM114, CBLN1,****USP10, KLHL36, PDILT, UMOD***^**q**^, *RP11-20I23.1, GCSH,****CTD-2144E22.5***, *NKD1*	^q^kidney disease	^67^
17	***KRTAP4-4, PIK3R5, PIK3R6, MEOX1, MAP2K3, KCNJ12, SLC47A2, LGALS3BP, FLJ45079***, *NLK, KRT37, KRT38, C17orf82, TBX4***, *NARF, CLEC10A, ASGR2, IKZF3***, *AC132872.2,****ZNF18, ENGASE***, *C1QTNF1, FAM211A, ZNF287*		
18	*ARHGAP28,****SLC14A2, MAPRE2***, *DSEL,****KIAA1468, PIGN***		
19	***TRPM4, RFX1, RLN3***, *PSG1,****ZNF600***, *ZNF28,****NOSIP, RCN3***, *NFKBID,****ARRDC2***, *DNMT1, EIF3G,****CATSPERG***, *AP3D1, DOT1L,****ECSIT***, *MIER2, AC018755.1, PLEKHJ1, TSHZ3*		
20	***RIMS4, CPNE1, RP1-309K20.6, WFDC12, FAM182B, ROMO1, NFS1, SPINT4***, *C20orf166, KCNB1, PTGIS,****DLGAP4***, *AAR2,****CST7, SLPI, MATN4***, *ARFGEF2,****ZSWIM3, ZSWIM1***, *PANK2*		
21	*TPTE*		
22	*KIAA1644, RP1-32I10.10,****CHEK2, TTC38***, *FAM118A, SMC1B, LDOC1L,****USP41, APOL4***^**r**^**, *APOL2***^**r**^, *TUBA8, USP18, POLR2F,****MICALL1, EIF3L***	^r^pathogen immunity	^68^

Genes are extracted from the protein coding genes in the top 1% of 100 kb iHS Windows (Table S8) with each gene having a mean iHS > 3.0 in the Lugbara population. The genes in bold are those that also have evidence of selection in the Basoga population. Genes with superscripts are those that are associated with the phenotype in the “Associated Effect” Column.

### Signatures of Selection in the Lugbara but Not Basoga Populations

**Figure 6:**
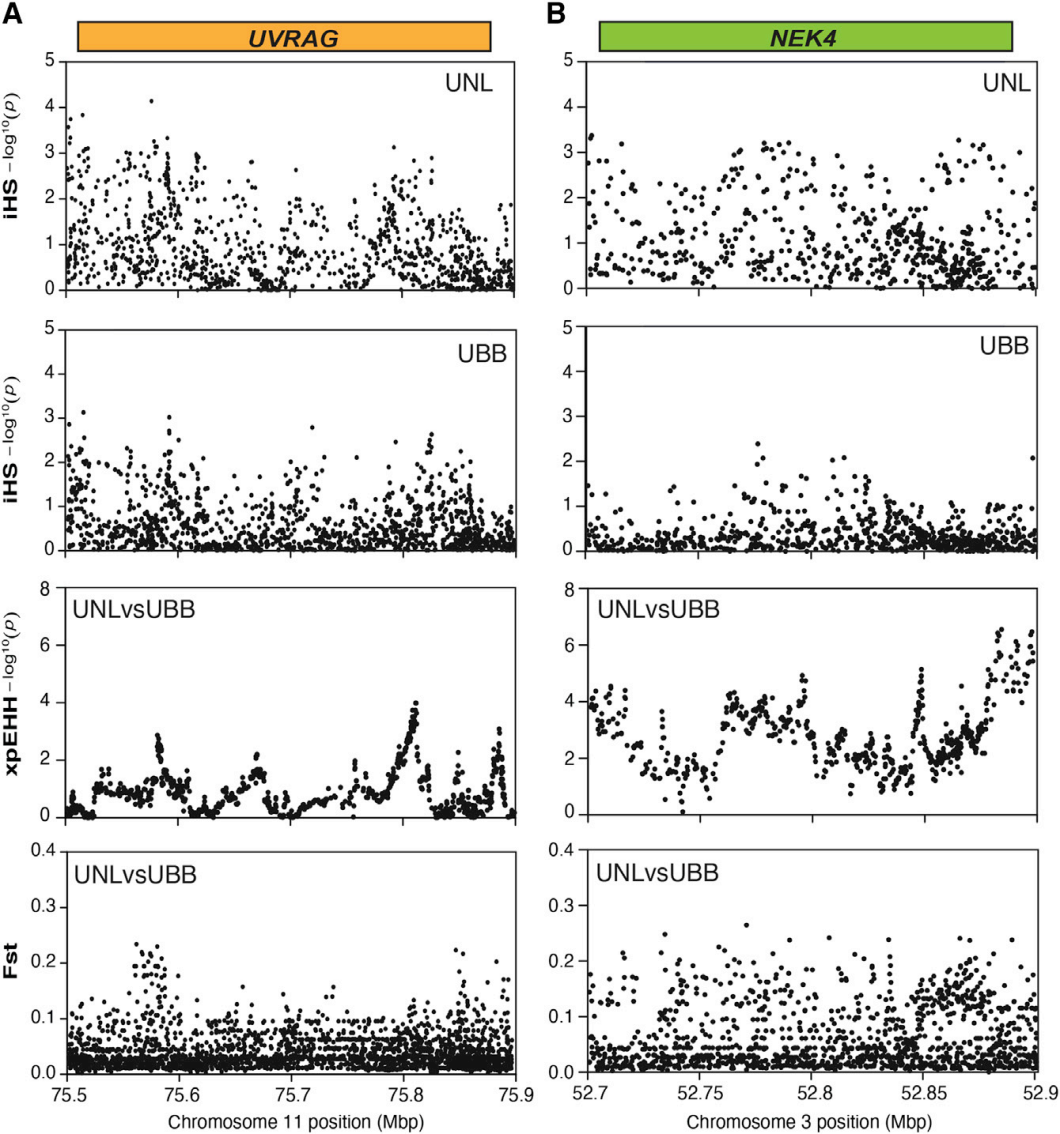
Signatures of Selection Unique to the Uganda Nilotic Lugbara Population Evidence (iHS, xpEHH, and Tajima D) for differential selection signatures between Lugbara (UNL) and Basoga (UBB) at the *UVRAG* locus on chromosome 11 (A) and the *NEK4* locus on chromosome 1 (B).

In order to identify SNPs associated with adaptation in the Lugbara population, we identified those selective sweeps in which the signature allele has achieved fixation in the Lugbara population but remains polymorphic in the Basoga population.^69^ We first identified loci within the Lugbara population that had extreme iHS values and occurred at a high frequency within a 100 kb window (SNPs having iHS > 2.0 and count > 20, Table S9). We then identified those that occur only in the UNL population (Table S10). Finally, we identified those genes with extreme iHS that are highly differentiated between the Lugbara and Basoga populations using high F_ST_ (top 5% quantile), high Tajima’s D, and high cross population EHH (xpEHH > 2.5). The three different metrics were combined by ranking genes on each individual metric and then obtaining the sum of the ranks for each gene (Table S11). From this we identified a set of top ranked genes (Table 2) which were highly differentiated between the Lugbara (UNL) and Basoga (UBB) populations. The three highest ranked genes were *NEK4,* which is associated with schizophrenia,^70^ *COLQ*, which is most highly expressed in CD8 T cells and CD56 NK cells,^71^^,^^72^ and *UVRAG*, which is involved in melanosome biogenesis and skin pigmentation^73^ and protection against UV radiation (Figure 6).

**Table 2:** Top-Ranked Extreme Signatures that Are Highly Differentiated between the Lugbara and Basoga Populations

**Chr**	**Gene**	**|iHS| Max**	**|iHS| Mean**	**Frequency iHS > 2**	**No.of SNPs iHS > 2**	**TajimaD_mean [UNL]**	**FST_Mean [UNL-UBB]**	**xpEHH_Max [UNL-UBB]**	**Rank Score**
3	*NEK4*	3.21	3.35	0.24	48/199	2.05	0.06	4.38	61
3	*COLQ*	4.15	3.37	0.23	43/189	1.92	0.02	3.58	62
11	*UVRAG*	4.14	3.31	0.23	72/312	1.73	0.03	3.88	68
7	*FAM3C*	4.87	3.10	0.19	51/265	2.40	0.04	2.94	70
12	*MGAT4C*	3.63	3.65	0.23	66/283	1.95	0.02	3.02	77
5	*ATP10B*	4.31	3.08	0.21	61/291	1.84	0.02	4.60	88
5	*TENM2*	3.44	3.19	0.34	104/305	1.73	0.01	4.23	90
3	*SMIM4*	4.04	3.07	0.27	57/208	0.36	0.05	3.57	91
11	*DGAT2*	4.14	3.26	0.23	72/312	1.45	0.02	2.32	95
5	*C5orf30*	3.50	3.04	0.17	38/218	2.34	0.05	3.42	101
3	*HACL1*	4.15	3.98	0.23	43/189	1.03	0.01	1.69	105
3	*GNL3*	3.21	3.00	0.24	48/199	2.05	0.08	2.67	106
10	*CYP2C8*	4.43	3.04	0.17	68/404	2.50	0.02	1.19	108
2	*ATP5G3*	3.70	3.21	0.17	48/279	1.82	0.01	3.32	111
10	*PDLIM1*	3.68	3.15	0.16	55/337	1.76	0.02	3.03	111
1	*WDR3*	3.80	3.18	0.15	21/136	1.61	0.01	4.17	113
22	*POLR2F*	4.99	3.35	0.23	45/200	0.88	0.00	1.26	115
14	*TEX22*	3.23	3.34	0.15	38/262	2.30	0.02	2.53	117
10	*C10orf129*	3.68	3.03	0.16	55/337	3.46	0.04	1.86	119
3	*DUSP7*	3.57	3.17	0.26	43/165	0.12	0.03	1.79	122

Genes were ranked separately for xpEHH, F_ST_, and Tajima D. The rank score was obtained by ranking genes separately by Tajima D, F_ST_, and xpEHH and then an overall score was obtained by summing the ranks of the three metrics.

## Discussion

### SNP Discovery

Africa has the most genetically diverse populations on earth but while there are projects to sequence in excess of 100,000 genomes from populations in Europe,^74^ Asia,^75^ and the Americas^76^ the 1000 Genomes Project is still the single largest dataset for Africa with 661 genome sequences. Not only do African genomes have a greater density of polymorphisms than genomes elsewhere, they also frequently have shorter haplotypes, which require a greater density of markers to phase accurately.^77^ To date, most African genome-wide association studies (GWASs) have been undertaken using chips designed for West Eurasian populations. This can severely limit researchers’ power to discover loci controlling disease. For example, a GWAS to identify loci regulating severe malaria failed to recapture the sickle cell locus because of limited linkage between markers and the functional SNP.^78^ Our sequence data from six Niger-Congo populations and the Nilo-Saharan Lugbara have already contributed to the development of an Illumina Omni chip that is enriched for African SNPs and should reduce the number of important loci missed by GWASs in African populations.^79^

### Demographic Inference

In this study, we carried out whole-genome sequencing on populations from six different sub-Saharan African countries, and combined our data with genome sequences from the 1000 Genomes and African Genome Variation projects to better understand the relationship of the Lugbara to neighboring populations. The great diversity of Nilo-Saharan languages meant that they were recognized as belonging to a single family only in 1966 and there is still a debate about whether all these languages share a common root.^80^ The Lugbara belong to the large Central Sudanic group of languages, while the Gumuz language has been hard to classify within the Nilo-Saharan family; the language may be an early branch from the family or it may be a language isolate and not related to Nilo-Saharan languages at all.^12^ Genetic evidence has shown that Gumuz speakers are closely related to other Nilo-Saharan speaking groups from West Ethiopia, Sudan, and Sud-Sudan^5^ and are well differentiated from neighboring Afro-Asiatic populations (Figure 2 and Table S5A). Our data show that F_ST_ between the Lugbara and the Gumuz (0.025) exceeds that between African Niger-Congo A and Niger Congo B populations (mean = 0.008, SE 0.0005) and also exceeded that within European, East Asian, and South Asian populations but not the American population in the 1000 Genomes data (Tables S5B and S5C). This is consistent with the relatively large F_ST_ between the Lugbara and the Gumuz being caused by differences in admixture history as well as isolation.

The two Nilo-Saharan populations also appeared very different in the F3 analyses (Figures 4 and S8). The Gumuz was most similar to the Afro-Asiatics with respect to their African component, in that there was evidence of shared ancestry to the Luhya (Figure 4A) when paired with any Niger-Congo B or Nigerian population and to the Basoga (Figure 4B) when paired with the Zambian population. The Lugbara, in contrast, appeared as a source population for the Basoga and Luhya only when paired with the Zambian population. This difference is surprising given the similarity of the two Nilo-Saharan populations in the admixture plots at most values of K. The patterns of genetic contribution from the Lugbara and Gumuz to the Luhya and Basoga in the F3 data are most consistent with the Admixture data at K = 6 where Gumuz but not Lugbara share a small ancestry component with the Afro-Asiatics. This component (pink) is also present in the Luhya but is marginal in the Basoga (Figure 3; K = 6). This component shared between the Gumuz, Basoga, and Luhya may represent an ancient East African population that was present before the Bantu Expansion.

The data are consistent with the Gumuz being genetically members of the Nilo-Saharan family and not an isolate, as some linguists have suggested.^10^^,^^12^ The large genetic distance between the Lugbara and Gumuz may be indicative of the deep splits within the Nilo-Saharan family, which merit much greater efforts to capture. A recent study included 2–4 samples from each of 9 lineages, supports the large genetic diversity within this family, and indicates that this family is a rich source of novel genetic variation.^6^ With sequence information from further Nilo-Saharan populations, the genetic relationship of the Lugbara and Gumuz to other members of the family will also be resolved.

### Signatures of Selection

We identified signatures of selection in multiple genes associated with immune responses and other conditions. However, the multiple and diverse functions of individual genes make it hard to predict the specific adaptations or phenotypes that might have driven selection at these loci. Nevertheless, there was a group of genes associated with skin tone and hair form which are plausibly associated with the particularly dark color of the skin of Nilo-Saharans and the intense UV radiation they experience. *UVRAG* showed the third greatest combined evidence for selection in Lugbara but not Basoga (Table 2). This gene, which is involved in melanine deposition in response to ultraviolet (UV) radiation,^73^ has not previously been found under selection. Two other genes involved in skin pigmentation (*SNX13* and *TYROBP*) were in the top 1% of gene regions under selection in Lugbara and were also under selection in Basoga (Table S8) and a further five genes involved in skin pigmentation (*IRF4, TYRP1, HERC2, SLC24A5, OPRM1*) had some evidence of selection (Table S7).^81^ Therefore, 7 of the 18 genes previously associated with skin pigmentation by Martin et al.^81^ had some evidence of selection in this study.

Nilo-Saharans have some of the darkest skin tones in the world^82^ and the Lugbara generally have a darker skin compared to the Basoga.^83^ Skin reflectance is correlated with UV radiation^84^ and the dark skin tones of the Nilo-Saharans could be an adaptation to the open savannah conditions of the Sahel where there is limited tree and cloud cover and which is predicted by models to be one of the regions of the world with darkest skin pigmentation.^84^ *UVRAG* may be an important contributor to the exceptionally dark skin tones of the Nilo-Saharans in conjunction with *SNX13* and *TYROBP* in particular and possibly also *IRF4, TYRP1, HERC2, SLC24A5,* and *OPRM1.*

Hair form is probably related to thermoregulation by helping keep the head cool during exercise.^85^ 6 keratin and 16 keratin-associated proteins, which are involved in hair formation, were in 3 regions with evidence of selection on chromosomes 12, 17, and 21 (Table S7) and selection for hair form as well as skin color could be part of a suite of traits for adaptation to the harsh conditions of the Sahel where the majority of Nilo-Saharan populations are found.

In conclusion, the Nilo-Saharan language speakers are an under-represented source for discovery of genetic variation. They are more genetically differentiated than the neighboring Afro-Asiatic and Niger-Congo groups but have been much less studied. They have contributed a large component to the genome of Afro-Asiatic speakers^26^ and a smaller proportion of the genomes of East African Niger-Congo-B speakers. There is evidence for selection for skin color and hair form, which could be adaptive for the semi-arid Sahel where the majority of Nilo-Saharan populations live. Linguistic evidence suggests that substantial further genetic diversity remains to be discovered within the Nilo-Saharan group, which should be a priority for further genome analysis studies.

## Declaration of Interests

The authors declare no competing interests.

## Data and Code Availability

The datasets generated and/or analyzed during the current study are available from the corresponding author on reasonable request. The sequenced data have been submitted to the EGA by H3ABionet under the study accession number EGA: EGAS00001002602.

## Web Resources

European Genome-phenome Archive (EGA), https://www.ebi.ac.uk/ega
QGIS, https://qgis.org/en/site/

## Supplemental Data

Data S1. Tables S6–S11

Document S2. Article plus Supplemental Information
