# Supplementary material for: High Levels of Genetic Diversity within Nilo-Saharan Populations: Implications for Human Adaptation
Source: Am J Hum Genet. 2020 Aug 10;107(3):473–86. doi: 10.1016/j.ajhg.2020.07.007 (PMC7477016; doi:10.1016/j.ajhg.2020.07.007)
Supplement: Document S1. Figures S1–S10 and Tables S1–S5 [file mmc1.pdf]

The American Journal of Human Genetics, Volume *107*

## Supplemental Data

# High Levels of Genetic Diversity within Nilo-Saharan Populations: Implications for Human Adaptation

**Julius Mulindwa, Harry Noyes, Hamidou Ilboudo, Luca Pagani, Oscar Nyangiri, Magambo Phillip Kimuda, Bernardin Ahouty, Olivier Fataki Asina, Elvis Ofon, Kelita Kamoto, Justin Windingoudi Kabore, Mathurin Koffi, Dieudonne Mumba Ngoyi, Gustave Simo, John Chisi, Issa Sidibe, John Enyaru, Martin Simuunza, Pius Alibu, Vincent Jamonneau, Mamadou Camara, Andy Tait, Neil Hall, Bruno Bucheton, Annette MacLeod, Christiane Hertz-Fowler, Enock Matovu, and the TrypanoGEN Research Group of the H3Africa Consortium**

## Supplemental Data

### Sequence Quality

Samples from five populations (CIV, GAS, UNL, UBB, DRC) were sequenced to 10X coverage and the remaining two populations (CAM and ZAM) were sequenced to 30X coverage.

There are two strategies within GATK for calling SNP from sequence data. 1) Combine the data from all samples and call SNP jointly and output just variant loci into a vcf file; 2) Call SNP on individual samples, output all loci into a gvcf file and combine the gvcf files later. The first strategy has the advantage of having more data to work with to assess quality metrics and cut-offs for SNP calling, however it is difficult to combine data that has been sequenced to different depths as different criteria need to be applied to each sample depending on depth of coverage. The second strategy is not affected by differences in sequence coverage and has the added advantage of making it easy to add data from additional samples as they become available without having to repeat the complete joint SNP calling on all samples. The second strategy was used in this project.

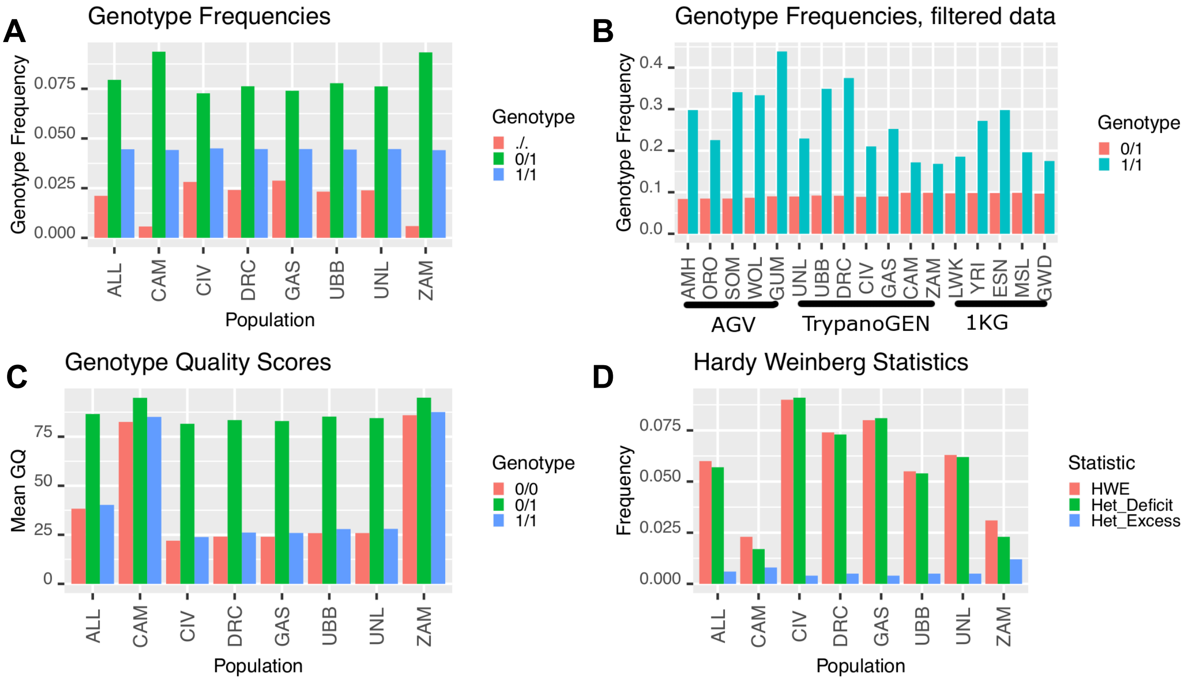


**Figure S1**. **Sequence Quality Metrics by Sample Population**. (**A**) **Genotype frequencies before filtering**, null genotypes are shown as (./.), heterozygotes (0/1) and homozygote alternate allele genotype (1/1). Homozygous reference genotypes (0/0) were the largest class (>80%) and are not shown for clarity. The small numbers of genotypes at multiallelic loci are also not shown. Note the lower frequency of null genotypes and higher frequency of heterozygotes in the Cameroon and Zambian (CAM and ZAM) populations, which were sequenced at 30X coverage whilst the other populations were sequenced at 10X. (**B**) **Genotype frequencies after filtering and merging**, heterozygotes (0/1) and homozygote minor (not alternate) genotypes (1/1), homozygous major genotypes (0/0) are not shown. Note that the frequency of heterozygotes is now very consistent across all populations irrespective of sequence depth, however the frequency of homozygous minor alleles is very variable across all data sources **(C) Mean Genotype Quality scores before filtering and phasing**. In the Cameroon and Zambian populations the Genotype quality scores were similar irrespective of genotype whilst in the populations sequenced at lower coverage the homozygote quality scores were substantially lower than the heterozygote scores. (**D**) **Hardy Weinberg Statistics before filtering and phasing.** The frequencies of loci with $p < 0.05$ are shown for three statistics: HWE, the Hardy Weinberg P value; Het_Deficit, $H_0$ the number of heterozygotes is not less than expected; Het_Excess, $H_0$ the number of heterozygotes is not greater than expected. The Cameroon

and Zambian populations had about a third of the number of loci that were not in hardy Weinberg Equilibrium as the other populations. In these two populations a higher proportion of loci that were not in Hardy Weinberg Equilibrium had an excess of heterozygotes and a lower proportion had a deficit of heterozygotes.

Figure S1 shows some descriptive statistics for sequence quality for each population and shows that there are clear differences between the samples sequenced at 30X (CAM and ZAM) and those sequenced at 10X before filtering and phasing however after filtering there were no differences that correlated with data source. Fig S1A shows that the frequency of null calls was much higher in the 10X-sequenced samples with a call rate of 97.4% in the 10X samples and 99.4% in the 30X samples. The 30X-sequenced samples also had higher proportions of heterozygotes (9.3%) compared with the 10X sequenced samples (7.5%). Therefore about 1.8% of homozygous calls are likely to be false and should have been called as heterozygotes. This is a known problem with low coverage data and is reflected in the Gentotype Quality (GQ) Scores for the different genotypes (Fig S1C). All samples had high GQ scores for heterozygote loci (Mean 10X = 84; Mean 30X=95), but the homozygotes had much lower scores in the 10X data (Mean 10X = 25; Mean 30X=85) reflecting the lower confidence that a heterozygote has not been missed with 10X data. After filtering and phasing (including imputation of missing data) (Fig S1B) all populations had very similar heterozygote frequencies irrespective of data source. Although the homozygous minor genotype frequency was very variable, it did not correlate with batch suggesting that this was genuine population variation rather than batch effect.
The higher frequency of missing heterozygotes in the 10X data before filtering and phasing is also reflected in the Hardy Weinberg statistics (Fig S1D); 2.7% of loci had $p<0.05$ for Hardy Weinberg equilibrium in the 30X data and 7.3% in the 10X data. Almost all of these loci in all datasets had a deficiency of heterozygotes (Fig S1C). Whilst it is expected that some loci will not be in Hardy-Weinberg equilibrium due to random sampling and also due to selection at some loci for particular alleles, the much higher frequency of loci with low Hardy-Weinberg P values in the 10X data reflects the rate of missing heterozygotes in the unfiltered data.

Despite the evidence that the 10X data quality was generally worse than the 30X quality there was very little evidence of this having a impact on the conclusions. The unfiltered data was only used for describing novel variants and their potential impacts. The filtering and phasing strategy generated a dataset with very similar heterozygote frequencies. The population analyses showed that geographically close populations from the same major linguistic group clustered tightly together irrespective of data source, demonstrating the success of the filtering strategy. In the multidimensional scaling analyses our West African samples (GAS, CIV) with 10X coverage clustered tightly with 1000 Genomes samples from West Africa (MSL, GWD) as expected and our UBB population from Uganda clustered tightly with the 1000 Genomes LWK samples from neighbouring Kenya. Furthermore in the Admixture analysis the number and size of ancestral components were very similar from adjacent 1000 Genomes and our data.

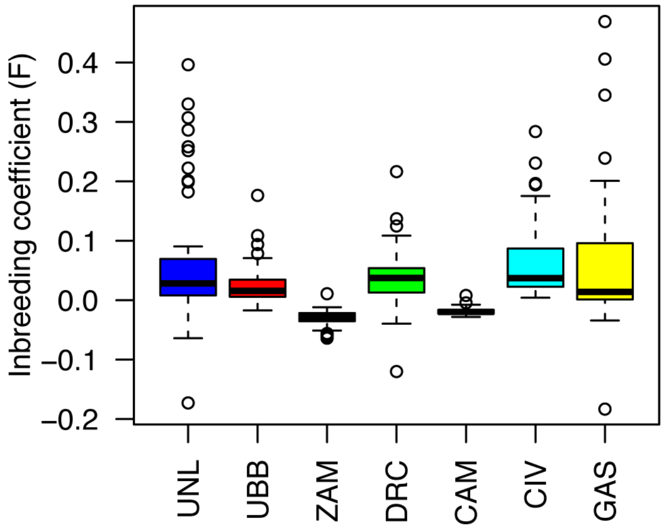


**Figure S2.** Heterozygosity analysis of the inbreeding coefficient within populations.

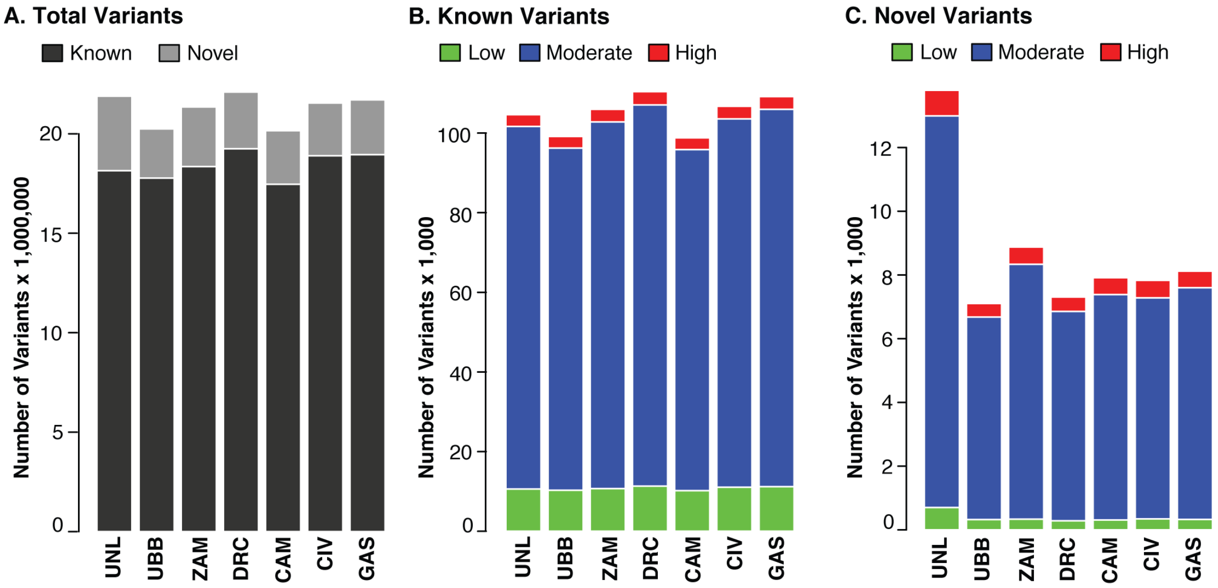


**Figure S3. Classification of the genetic variation in the sequenced populations. A**. The total number of both the Known (with dbSNP rsID) and Unknown/Novel (without dbSNP rsID) variants; The degree of impact on genome function, predicted by SnpEff, is shown for the Known (**B**) and Unknown/Novel (**C**) variants (see Table S6 for definitions of impacts). SNPs that were classified as "modifier" were mainly in intergenic regions and are excluded from the plot. Variants with multiple impact annotations were assigned to the highest impact annotation. (See Table S4 for the underlying data)

A B

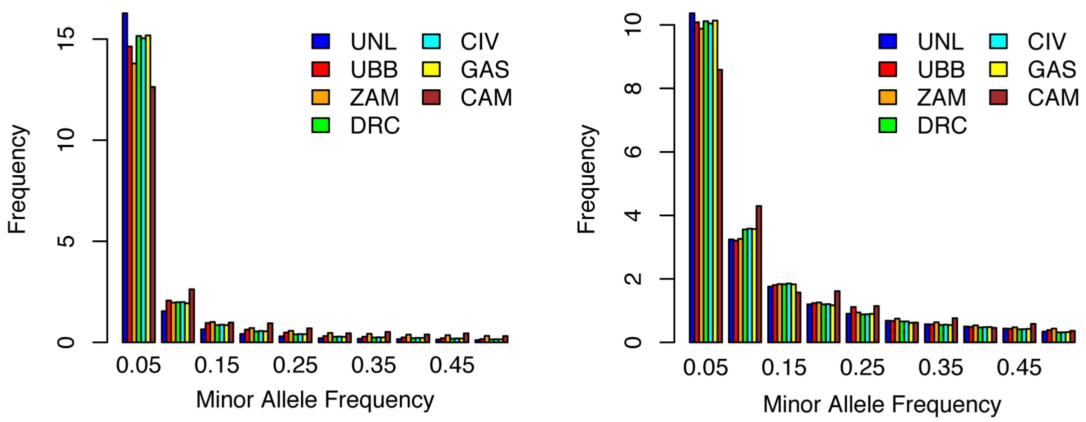


**Figure S4**. **A**. Minor allele frequency (MAF) distribution of Novel variants, **B**. MAF distribution of known variants.

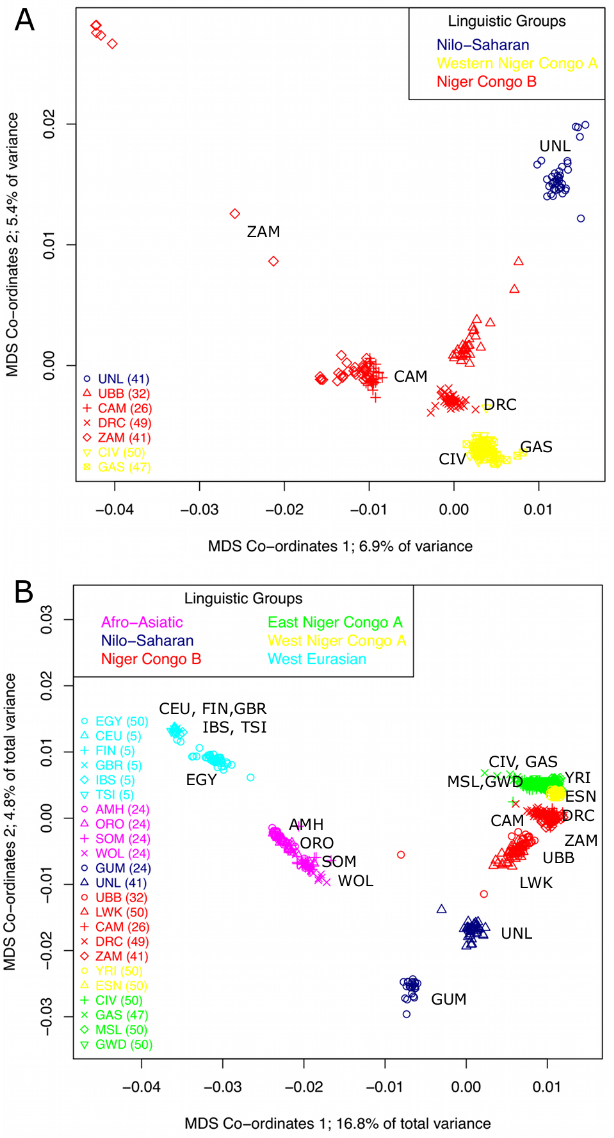


**Figure S5**. Multidimensional scaling analysis on (**A**) TrypanoGEN populations (**B**) African and European populations**.** Both plots include the 7 Zambian outlier samples that were excluded from Fig2A (but not Fig2B). In (A) the 7 outliers are widely dispersed but in (B) in the much larger context they cluster tightly with the remaining Zambian samples. The numbers in brackets beside each population indicate the number of individuals whose genomes were analysed.

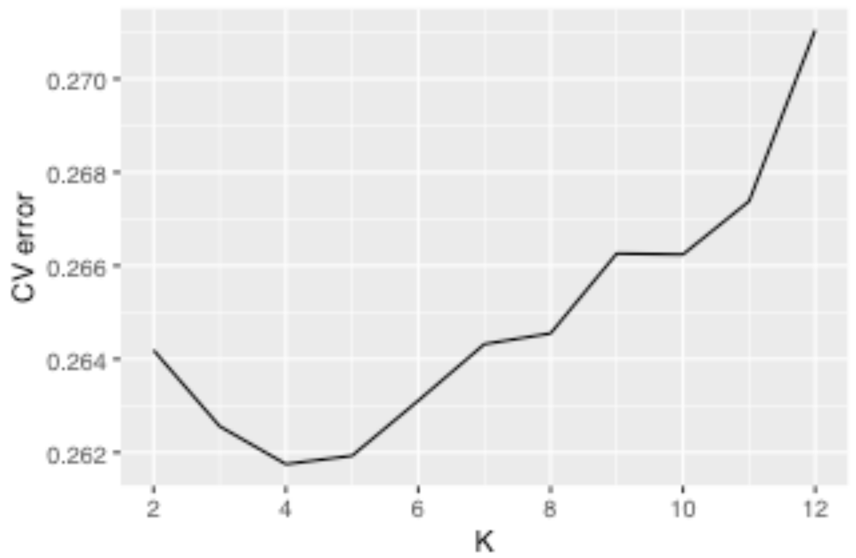


**Figure S6.** Admixture analysis cross validation (CV) errors**.** Plot of the admixture cross validation error verses the number of clusters (K) for the TrypanoGEN, 1000 Genomes and AGVP dataset.

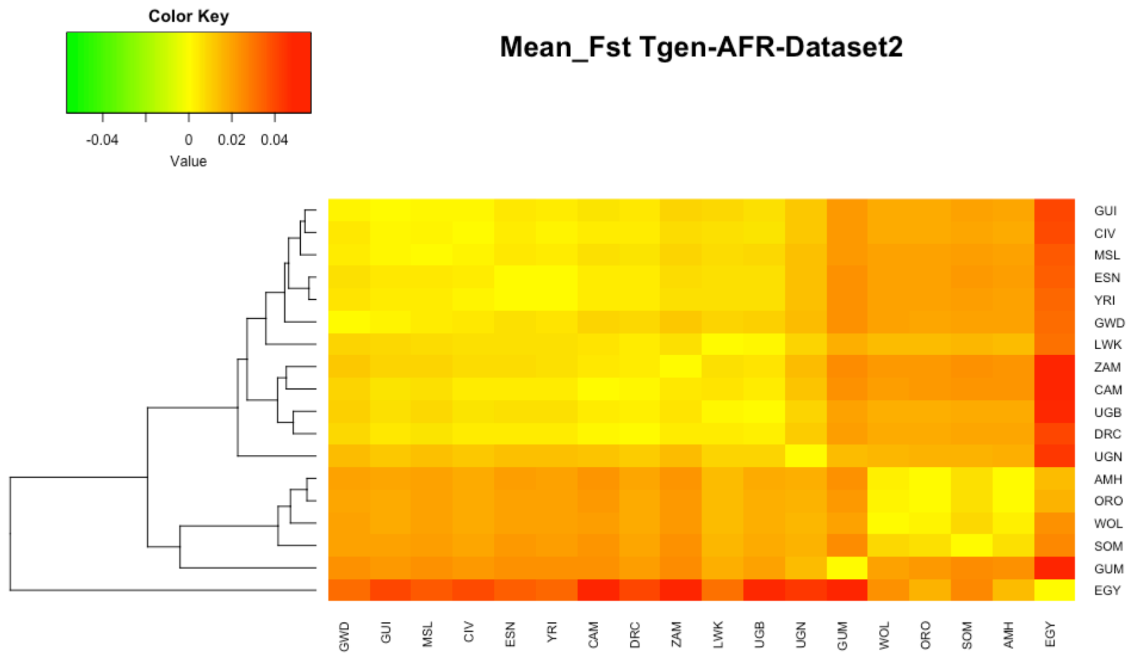


**Figure S7**. Heatmap of mean Fst between TrypanoGEN and 1000 genome African populations.

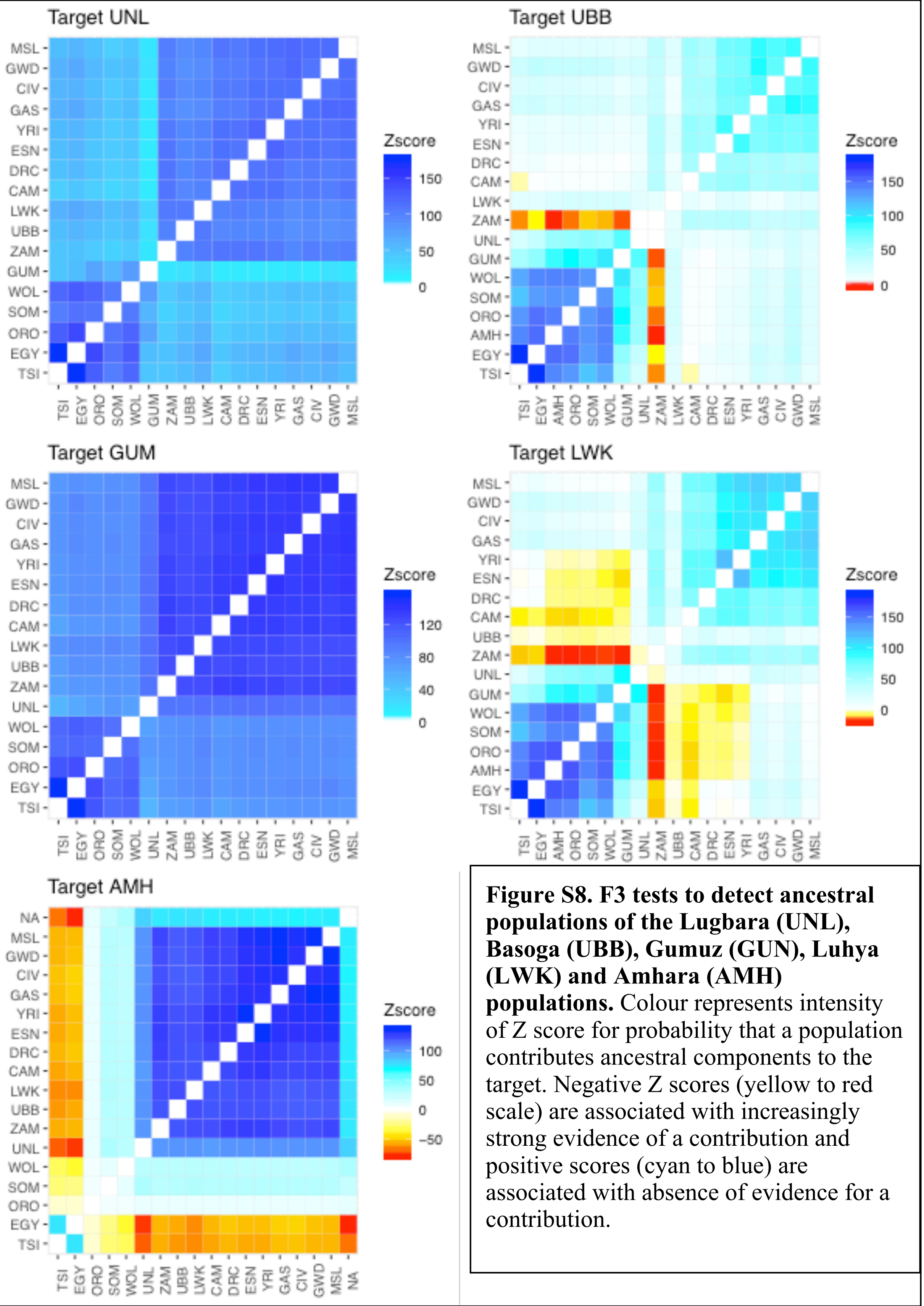


**Figure S8. F3 tests to detect ancestral populations of the Lugbara (UNL), Basoga (UBB), Gumuz (GUN), Luhya (LWK) and Amhara (AMH) populations.** Colour represents intensity of Z score for probability that a population contributes ancestral components to the target. Negative Z scores (yellow to red scale) are associated with increasingly strong evidence of a contribution and positive scores (cyan to blue) are associated with absence of evidence for a contribution.

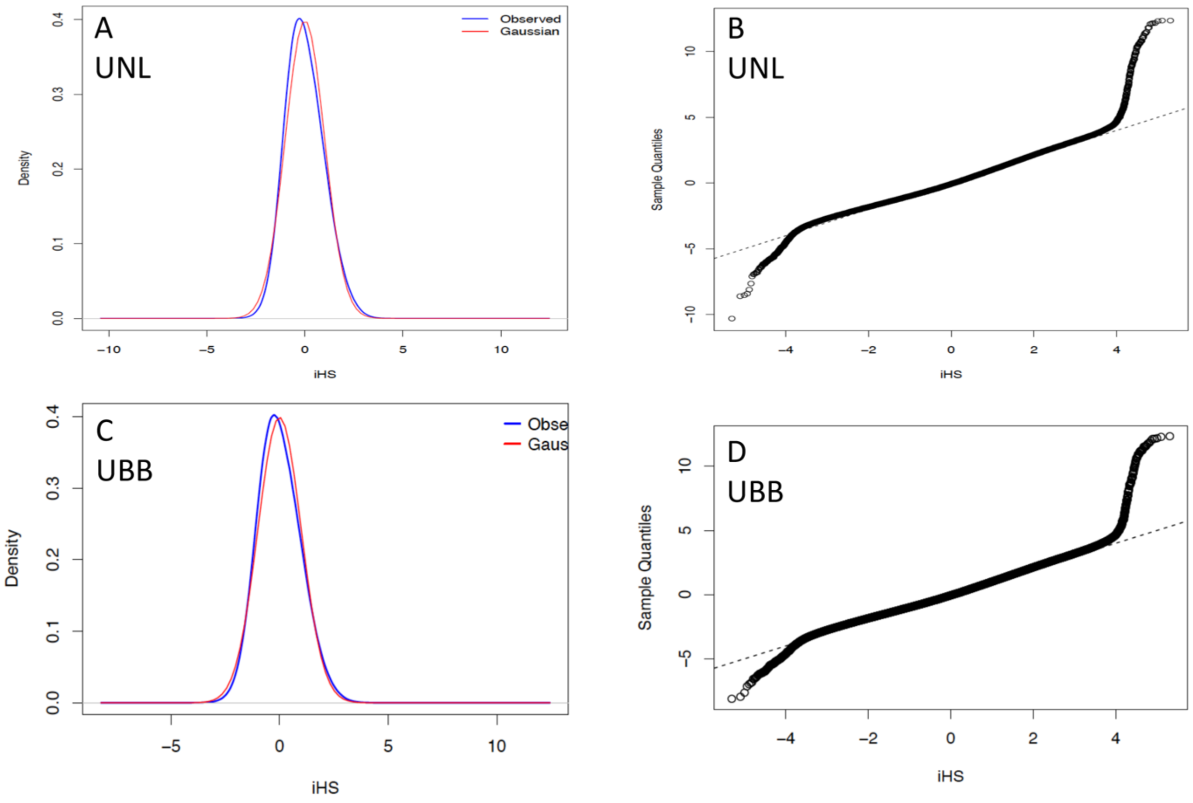


**Figure S9**. Analysis for signatures of selection in the Uganda Lugbara and Basoga populations. The UNL population **A.** genome wide density distribution histogram of the observed iHS values with respect to the Gaussian model and **B.** Q-Q plot of the genome wide iHS distribution for which the top iHS>3.0 were considered for selection analysis. **C** and **D** are the genome wide distribution and Q-Q plots respectively for the UBB population.

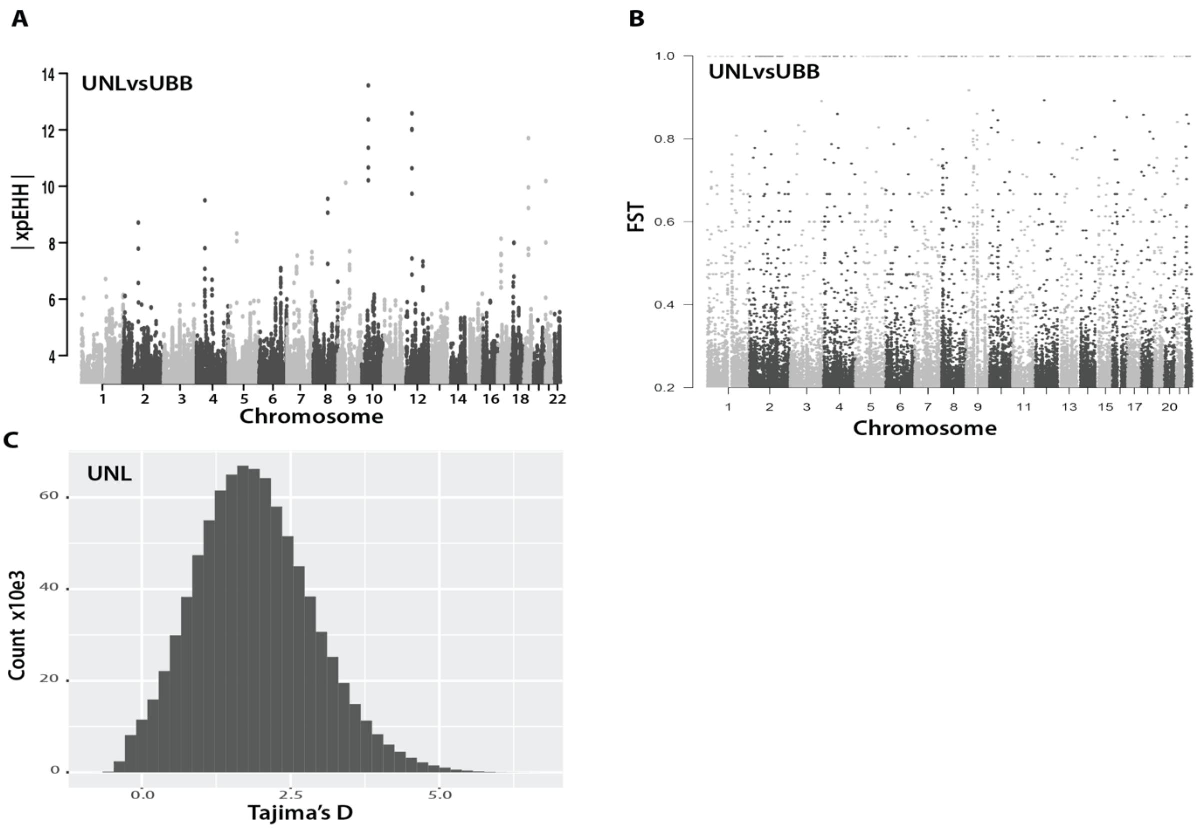


**Figure S10**. Genome wide signatures of selection that are differentiated between the UNL and UBB populations. **A.** The cross population extended haplotype analysis showing the xpEHH > 3.0. **B.** Genome wide distribution of FST>0.2 between the UNL and UBB populations. **C.** Normal distribution of the Tajima's D scores within the UNL population.

## Supplemental tables

| Country | Code | Ethnicity | Language Family and Major Branch | Ethnologue code | No. of Samples | Sample Source |
|---|---|---|---|---|---|---|
| Uganda | UNL | Lugbara | Nilo-Saharan, Central Sudanic | lgg | 50 | Tgen |
| | UBB | Basoga | Atlantic-Congo, Benue-Congo | xog | 33 | Tgen |
| Zambia | ZAM | Soli/Chikunda | Atlantic-Congo, Benue-Congo | sby; kdn | 25 | Tgen |
| | | Tumbuka | Atlantic-Congo, Benue-Congo | tum | 13 | Tgen |
| | | Bemba | Atlantic-Congo, Benue-Congo | bem | 3 | Tgen |
| Congo | DRC | Kimbala | Atlantic-Congo, Benue-Congo | mdp | 20 | Tgen |
| | | Kingongo | Atlantic-Congo, Benue-Congo | noq | 30 | Tgen |
| Cameroon | CAM | Bamilike | Atlantic-Congo, Benue-Congo | fmp | 6 | Tgen |
| | | Mundani | Atlantic-Congo, Benue-Congo | mnf | 8 | Tgen |
| | | Ngoumba | Atlantic-Congo, Benue-Congo | nmg | 12 | Tgen |
| Ivory Coast | CIV | Baoule | Atlantic-Congo, Kwa | bci | 11 | Tgen |
| | | Gouro | Mande | goa | 21 | Tgen |
| | | More | Mande | Moa | 12 | Tgen |
| | | Senoufo | Atlantic-Congo, Senufo | sef | 4 | Tgen |
| | | Malinke | Mande | loi | 1 | Tgen |
| | | Koyaka | Mande | kga | 1 | Tgen |
| Guinea | GAS | Soussou | Mande | sus | 50 | Tgen |
| Ethiopia | GUM | Gumuz | Nilo-Saharan, Kumuz | guk | 24 | AGVP |
| | AMH | Amharic | Afro-Asiatic, Semitic | amh | 24 | AGVP |
| | ORO | Oromo | Afro-Asiatic, Cushitic | hae | 24 | AGVP |
| | WOL | Wolaytta | Afro-Asiatic, Omotic | wal | 24 | AGVP |
| | SOM | Somali | Afro-Asiatic, Cushitic | som | 24 | AGVP |
| Egypt | EGY | Arabic | Afro-Asiatic, Semitic | arz | 50 | AGVP |
| Gambia | GWD | Mandika | Mande | mnk | 50 | 1000G |
| Sierra Leone | MSL | Mende | Mande | men | 50 | 1000G |
| Nigeria | ESN | Esan | Atlantic-Congo, Volta-Niger | ish | 50 | 1000G |
| | YRI | Yoruba | Atlantic-Congo, Volta-Niger | yor | 50 | 1000G |
| Kenya | LWK | Luhya | Atlantic-Congo, Benue-Congo | luy | 50 | 1000G |

**Table S1.** Ethno-linguistic classification of samples used for analysis. The Code is the abbreviation used for the group in the text and legends. Codes were assigned as follows: 1) name in original publication if previously published, 2) TrypanoGEN samples from a single country that clustered together on the MDS plot were designated as a population and assigned an abbreviation. Where there was a single linguistic group in a cluster from a country we referred to the samples from that cluster by a three letter code that consisted of, 1. Country/ geographical localisation, 2. Major Ethnic group and 3. Linguistic group. Eg GAS for Guinea, Niger-Congo-A, Soussou. For other clusters from a country where there were samples from multiple linguistic groups we referred to those samples by a three letter code for the country.

| Cameroon (CAM) | | | Ivory Coast (CIV) | | | Republic of Congo (DRC) | | | Uganda, Basoga (UBB) | | | Uganda Lugbara (UNL) | | | Zambia (ZAM) | | | Guinea (GAS) | | |
|---|---|---|---|---|---|---|---|---|---|---|---|---|---|---|---|---|---|---|---|---|
| **Seq_ID** | **Ethnicity** | **Sex** | **Seq_ID** | **Ethnicity** | **Sex** | **Seq_ID** | **Ethnicity** | **Sex** | **Seq_ID** | **Ethnicity** | **Sex** | **Seq_ID** | **Ethnicity** | **Sex** | **Seq_ID** | **Ethnicity** | **Sex** | **Seq_ID** | **Ethnicity** | **Sex** |
| CB12 | Ngoumba | M | CIV_1 | Gouro | F | DRC_1 | Kingongo | M | UGB013C | Basoga | F | UGN005T | Lugbara | F | ZC08 | Tumbuka | M | GUI_1 | Soussou | F |
| CB14 | Ngoumba | F | CIV_10 | BaoulÈ | M | DRC_10 | Kingongo | F | UGB015C | Basoga | M | UGN006C | Lugbara | F | ZC09 | Tumbuka | M | GUI_10 | Soussou | M |
| CB15 | Ngoumba | M | CIV_11 | MorÈ | M | DRC_11 | Kingongo | F | UGB020C | Basoga | M | UGN044T | Lugbara | M | ZC10 | Tumbuka | M | GUI_11 | Soussou | F |
| CB16 | Ngoumba | M | CIV_12 | MorÈ | F | DRC_12 | Kingongo | F | UGB022C | Basoga | F | UGN045C | Lugbara | M | ZC11 | Tumbuka | F | GUI_12 | Soussou | F |
| CB17 | Ngoumba | M | CIV_13 | MorÈ | M | DRC_13 | Kingongo | F | UGB029C | Basoga | F | UGN046T | Lugbara | M | ZC13 | Tumbuka | M | GUI_13 | Soussou | F |
| CB24 | Ngoumba | M | CIV_14 | MorÈ | F | DRC_14 | Kimbala | F | UGB038C | Basoga | F | UGN048T | Lugbara | M | ZC14 | Tumbuka | M | GUI_14 | Soussou | M |
| CB29 | Ngoumba | M | CIV_15 | Gouro | M | DRC_15 | Kingongo | F | UGB039C | Basoga | F | UGN063T | Lugbara | F | ZC22 | Tumbuka | F | GUI_15 | Soussou | F |
| CB31 | Ngoumba | M | CIV_16 | Gouro | M | DRC_16 | Kingongo | M | UGB040C | Basoga | F | UGN064C | Lugbara | F | ZC23 | Tumbuka | M | GUI_16 | Soussou | F |
| CB32 | Ngoumba | F | CIV_17 | Gouro | M | DRC_17 | Kingongo | M | UGB044C | Basoga | M | UGN065C | Lugbara | F | ZC24 | Tumbuka | M | GUI_18 | Soussou | F |
| CB33 | Ngoumba | F | CIV_18 | Gouro | M | DRC_18 | Kingongo | F | UGB046C | Basoga | F | UGN068C | Lugbara | M | ZC25 | Tumbuka | M | GUI_2 | Soussou | M |
| CB7 | Ngoumba | F | CIV_19 | MorÈ | F | DRC_19 | Kingongo | M | UGB047C | Basoga | F | UGN069T | Lugbara | M | ZC26 | Tumbuka | M | GUI_20 | Soussou | M |
| CF24 | Mundani | M | CIV_2 | BaoulÈ | M | DRC_2 | Kimbala | F | UGB049C | Basoga | F | UGN070C | Lugbara | M | ZC27 | Tumbuka | F | GUI_21 | Soussou | F |
| CF25 | Mundani | M | CIV_20 | BaoulÈ | F | DRC_20 | Kingongo | F | UGB050C | Basoga | F | UGN071T | Lugbara | M | ZC28 | Tumbuka | F | GUI_23 | Soussou | M |
| CF26 | Mundani | F | CIV_21 | MorÈ | F | DRC_21 | Kingongo | M | UGB051C | Basoga | F | UGN072C | Lugbara | M | ZM18 | Bemba | M | GUI_24 | Soussou | M |
| CF27 | Mundani | M | CIV_22 | Gouro | F | DRC_22 | Kingongo | F | UGB056C | Basoga | F | UGN073C | Lugbara | M | ZM20 | Bemba | M | GUI_25 | Soussou | F |
| CF30 | Mundani | M | CIV_23 | Gouro | M | DRC_23 | Kingongo | M | UGB059C | Basoga | F | UGN074T | Lugbara | F | ZM21 | Bemba | F | GUI_26 | Soussou | M |
| CF37 | Mundani | M | CIV_24 | Gouro | F | DRC_24 | Kingongo | M | UGB062C | Basoga | M | UGN075C | Lugbara | F | ZR01 | Soli/Chikunda | M | GUI_29 | Soussou | M |
| CF46 | Mundani | F | CIV_25 | Gouro | M | DRC_25 | Kingongo | M | UGB066C | Basoga | M | UGN076T | Lugbara | F | ZR02 | Soli/Chikunda | M | GUI_3 | Soussou | M |
| CF49 | Mundani | F | CIV_26 | Gouro | M | DRC_26 | Kingongo | F | UGB067C | Basoga | M | UGN077C | Lugbara | F | ZR04 | Soli/Chikunda | M | GUI_30 | Soussou | M |
| CP17 | Bamilike | F | CIV_27 | Gouro | M | DRC_27 | Kimbala | M | UGB068C | Basoga | F | UGN079C | Lugbara | F | ZR05 | Soli/Chikunda | M | GUI_31 | Soussou | M |
| CP28 | Ngoumba | M | CIV_28 | Gouro | M | DRC_28 | Kingongo | F | UGB071C | Basoga | M | UGN080C | Lugbara | F | ZR06 | Soli/Chikunda | M | GUI_32 | Soussou | F |
| CP36 | Bamilike | F | CIV_29 | Gouro | M | DRC_29 | Kingongo | M | UGB072C | Basoga | F | UGN082C | Lugbara | M | ZR07 | Soli/Chikunda | M | GUI_33 | Soussou | F |
| CP38 | Bamilike | M | CIV_3 | BaoulÈ | M | DRC_3 | Kimbala | F | UGB073C | Basoga | F | UGN088C | Lugbara | M | ZR29 | Soli/Chikunda | F | GUI_34 | Soussou | M |
| CP40 | Bamilike | F | CIV_30 | MorÈ | M | DRC_30 | Kimbala | F | UGB074C | Basoga | F | UGN090C | Lugbara | M | ZR30 | Soli/Chikunda | F | GUI_35 | Soussou | F |
| CP48 | Bamilike | F | CIV_35 | BaoulÈ | F | DRC_31 | Kimbala | F | UGB077C | Basoga | F | UGN091C | Lugbara | M | ZR31 | Soli/Chikunda | F | GUI_36 | Soussou | F |
| CP9 | Bamilike | M | CIV_36 | Koyaka | F | DRC_32 | Kimbala | F | UGB079C | Basoga | F | UGN092C | Lugbara | M | ZR32 | Soli/Chikunda | M | GUI_37 | Soussou | M |

| | | | | | | | | | | | | | | | | | | |
|---|---|---|---|---|---|---|---|---|---|---|---|---|---|---|---|---|---|---|
| | CIV_37 | BaoulÈ | F | DRC_33 | Kimbala | M | UGB105C | Basoga | F | UGN093C | Lugbara | M | ZR33 | Soli/Chikunda | F | GUI_38 | Soussou | F |
| | CIV_38 | BaoulÈ | M | DRC_34 | Kingongo | M | UGB350C | Basoga | F | UGN098C | Lugbara | M | ZR34 | Soli/Chikunda | M | GUI_39 | Soussou | F |
| | CIV_39 | SÈnoufo | M | DRC_35 | Kingongo | M | UGB351C | Basoga | M | UGN099C | Lugbara | F | ZR35 | Soli/Chikunda | F | GUI_4 | Soussou | M |
| | CIV_4 | Gouro | M | DRC_36 | Kingongo | F | UGB369C | Basoga | M | UGN100C | Lugbara | F | ZR36 | Soli/Chikunda | M | GUI_40 | Soussou | M |
| | CIV_40 | Gouro | F | DRC_37 | Kingongo | F | UGB371C | Basoga | F | UGN105C | Lugbara | M | ZR37 | Soli/Chikunda | M | GUI_41 | Soussou | F |
| | CIV_41 | More | F | DRC_38 | Kingongo | M | UGB383C | Basoga | M | UGN106C | Lugbara | M | ZR38 | Soli/Chikunda | M | GUI_42 | Soussou | F |
| | CIV_42 | BaoulÈ | M | DRC_39 | Kimbala | M | UGB386C | Basoga | M | UGN107C | Lugbara | M | ZR39 | Soli/Chikunda | F | GUI_43 | Soussou | M |
| | CIV_43 | MorÈ | M | DRC_4 | Kimbala | F | | | | UGN109C | Lugbara | M | ZR40 | Soli/Chikunda | F | GUI_44 | Soussou | F |
| | CIV_44 | BaoulÈ | F | DRC_40 | Kimbala | M | | | | UGN113T | Lugbara | F | ZR41 | Soli/Chikunda | M | GUI_45 | Soussou | M |
| | CIV_45 | Gouro | M | DRC_41 | Kimbala | F | | | | UGN114C | Lugbara | F | ZR42 | Soli/Chikunda | M | GUI_46 | Soussou | F |
| | CIV_48 | BaoulÈ | M | DRC_42 | Kimbala | M | | | | UGN115C | Lugbara | F | ZR43 | Soli/Chikunda | F | GUI_47 | Soussou | M |
| | CIV_49 | SÈnoufo | F | DRC_43 | Kimbala | F | | | | UGN124T | Lugbara | M | ZR44 | Soli/Chikunda | F | GUI_48 | Soussou | M |
| | CIV_5 | Gouro | M | DRC_44 | Kimbala | M | | | | UGN125T | Lugbara | M | ZR46 | Soli/Chikunda | M | GUI_49 | Soussou | F |
| | CIV_50 | Gouro | M | DRC_45 | Kingongo | M | | | | UGN127C | Lugbara | F | ZR47 | Soli/Chikunda | F | GUI_5 | Soussou | M |
| | CIV_51 | Gouro | F | DRC_46 | Kimbala | F | | | | UGN134T | Lugbara | M | ZR49 | Soli/Chikunda | M | GUI_50 | Soussou | F |
| | CIV_52 | ND | M | DRC_47 | Kimbala | M | | | | UGN136T | Lugbara | F | | | | GUI_51 | Soussou | F |
| | CIV_53 | Gouro | M | DRC_48 | Kingongo | F | | | | UGN137C | Lugbara | F | | | | GUI_52 | Soussou | F |
| | CIV_54 | SÈnoufo | M | DRC_49 | Kingongo | M | | | | UGN140T | Lugbara | F | | | | GUI_53 | Soussou | F |
| | CIV_55 | Gouro | M | DRC_5 | Kimbala | M | | | | UGN142T | Lugbara | F | | | | GUI_54 | Soussou | F |
| | CIV_56 | MorÈ | M | DRC_50 | Kingongo | F | | | | UGN144T | Lugbara | M | | | | GUI_55 | Soussou | M |
| | CIV_6 | Gouro | M | DRC_6 | Kimbala | F | | | | UGN148T | Lugbara | F | | | | GUI_6 | Soussou | M |
| | CIV_7 | MalinkÈ | M | DRC_7 | Kingongo | F | | | | UGN153T | Lugbara | M | | | | GUI_7 | Soussou | F |
| | CIV_8 | MorÈ | F | DRC_8 | Kingongo | F | | | | UGN157T | Lugbara | F | | | | GUI_8 | Soussou | F |
| | CIV_9 | BaoulÈ | F | DRC_9 | Kimbala | F | | | | UGN185C | Lugbara | M | | | | GUI_9 | Soussou | M |

**Table S2.** Sample sequence identifier, ethnicity and sex of each participant whose DNA was sequenced

| **Filter** | **Count Loci** |
|---|---|
| Total Loci Discovered | 38,963,563 |
| Minor allele count < 3 | 16,840,310 |
| MAF < 0.01 | 4,764,259 |
| pHWE < 0.001 | 1,106,883 |
| Missing genotype data > 0.1 | 306,271 |
| Total loci passing QC | 15,945,840 |

**Table S3** Number of loci discovered and number removed by each filter**.** Note that the number of loci removed by a given filter will depend on the order in which filters are applied. We have listed filters in order of effect size.

| Variants | ZAM* | UBB | CIV | CAM* | DRC | GAS | UNL |
|---|---|---|---|---|---|---|---|
| **Total variants** | **21,346,657** | **20,244,883** | **21,546,091** | **20,154,485** | **22,100,090** | **21,703,906** | **21,891,961** |
| **Known_variants** | 18,348,704 | 17,773,731 | 18,895,407 | 17,466,021 | 19,245,113 | 18,948,607 | 18,145,718 |
| **Novel_variants** | 2,997,953 | 2,471,152 | 2,650,684 | 2,688,464 | 2,854,977 | 2,755,299 | 3,746,243 |
| **Known_low** | 10,705 | 10,316 | 11,046 | 10,187 | 11,337 | 11,207 | 10,626 |
| **Known_modifier** | 18,242,755 | 17,674,567 | 18,788,695 | 17,367,207 | 19,134,729 | 18,839,449 | 18,040,733 |
| **Known_moderate** | 92,096 | 85,916 | 92,517 | 85,651 | 95,710 | 94,746 | 91,028 |
| **Known_high** | 3,147 | 2,931 | 3,148 | 2,975 | 3,336 | 3,204 | 2,981 |
| **Novel_low** | 339 | 323 | 347 | 312 | 285 | 330 | 702 |
| **Novel_modifier** | 2,989,075 | 2,464,046 | 2,642,851 | 2,680,549 | 2,847,670 | 2,747,181 | 3,732,449 |
| **Novel_moderate** | 7,996 | 6,357 | 6,933 | 7,073 | 6,569 | 7,271 | 12,290 |
| **Novel_high** | 543 | 426 | 553 | 530 | 453 | 517 | 802 |

**Table S4**. The number of variants obtained from the mapping and variant calling pipeline for each population. All samples were sequenced at 10X coverage except those from *Zambia and *Cameroon, which were at 30X coverage. The variants that were annotated with a dbSNP rsID were termed 'Known_variants' whereas those without were termed 'Novel_variants'. The impact of the genomic variant as annotated by SnpEff were classified as 'Low', 'Modifier', 'Moderate' or 'High' based on their effect on transcription and/or translation. The Low impact variant features result in changes/mutations in the start & stop codons, splice site regions; Modifier variants affected mainly intergenic regions; Moderate impact variants features result in codon change, 3' & 5' UTR truncation exon loss, splice site branch region for U12 splicing machinery; High impact variant features occur in and affect chromosome deletion, exon deletion, frame shift, rare amino acid, splice site acceptor and donor, loss or gain of stop & start codons. Details of the classification are in table S6.

| | AMH | CAM | CIV | DRC | EGY | ESN | GAS | GUM | GWD | LWK | MSL | ORO | SOM | UBB | UNL | WOL | YRI | ZAM |
|---|---|---|---|---|---|---|---|---|---|---|---|---|---|---|---|---|---|---|
| **AMH** | 0.00000 | 0.04689 | 0.04462 | 0.04450 | 0.01703 | 0.04737 | 0.04450 | 0.04232 | 0.04470 | 0.03643 | 0.04676 | 0.00047 | 0.00981 | 0.03977 | 0.03659 | 0.00471 | 0.04624 | 0.04941 |
| **CAM** | 0.04689 | 0.00000 | 0.00585 | 0.00265 | 0.08740 | 0.00490 | 0.00732 | 0.03969 | 0.00908 | 0.00593 | 0.00680 | 0.04349 | 0.04284 | 0.00521 | 0.01585 | 0.03909 | 0.00454 | 0.00618 |
| **CIV** | 0.04462 | 0.00585 | 0.00000 | 0.00473 | 0.08127 | 0.00508 | 0.00242 | 0.04155 | 0.00604 | 0.00913 | 0.00366 | 0.04173 | 0.04163 | 0.00709 | 0.01734 | 0.03841 | 0.00375 | 0.01023 |
| **DRC** | 0.04450 | 0.00265 | 0.00473 | 0.00000 | 0.08183 | 0.00541 | 0.00644 | 0.03993 | 0.00949 | 0.00582 | 0.00769 | 0.04151 | 0.04112 | 0.00380 | 0.01581 | 0.03783 | 0.00497 | 0.00599 |
| **EGY** | 0.01703 | 0.08740 | 0.08127 | 0.08183 | 0.00000 | 0.08210 | 0.08120 | 0.08816 | 0.07802 | 0.07190 | 0.08220 | 0.01992 | 0.03472 | 0.07931 | 0.07969 | 0.03098 | 0.08066 | 0.08793 |
| **ESN** | 0.04737 | 0.00490 | 0.00508 | 0.00541 | 0.08210 | 0.00000 | 0.00716 | 0.04328 | 0.00710 | 0.00750 | 0.00513 | 0.04448 | 0.04397 | 0.00841 | 0.01980 | 0.04116 | 0.00080 | 0.00903 |
| **GAS** | 0.04450 | 0.00732 | 0.00242 | 0.00644 | 0.08120 | 0.00716 | 0.00000 | 0.04155 | 0.00343 | 0.01024 | 0.00278 | 0.04159 | 0.04154 | 0.00844 | 0.01790 | 0.03816 | 0.00581 | 0.01155 |
| **GUM** | 0.04232 | 0.03969 | 0.04155 | 0.03993 | 0.08816 | 0.04328 | 0.04155 | 0.00000 | 0.04254 | 0.03244 | 0.04236 | 0.03827 | 0.04041 | 0.03446 | 0.02525 | 0.03203 | 0.04235 | 0.04339 |
| **GWD** | 0.04470 | 0.00908 | 0.00604 | 0.00949 | 0.07802 | 0.00710 | 0.00343 | 0.04254 | 0.00000 | 0.01032 | 0.00360 | 0.04191 | 0.04166 | 0.01136 | 0.02030 | 0.03902 | 0.00589 | 0.01293 |
| **LWK** | 0.03643 | 0.00593 | 0.00913 | 0.00582 | 0.07190 | 0.00750 | 0.01024 | 0.03244 | 0.01032 | 0.00000 | 0.00912 | 0.03361 | 0.03336 | 0.00210 | 0.01258 | 0.03016 | 0.00690 | 0.00773 |
| **MSL** | 0.04676 | 0.00680 | 0.00366 | 0.00769 | 0.08220 | 0.00513 | 0.00278 | 0.04236 | 0.00360 | 0.00912 | 0.00000 | 0.04384 | 0.04350 | 0.00990 | 0.01993 | 0.04017 | 0.00395 | 0.01096 |
| **ORO** | 0.00047 | 0.04349 | 0.04173 | 0.04151 | 0.01992 | 0.04448 | 0.04159 | 0.03827 | 0.04191 | 0.03361 | 0.04384 | 0.00000 | 0.00885 | 0.03676 | 0.03335 | 0.00354 | 0.04344 | 0.04621 |
| **SOM** | 0.00981 | 0.04284 | 0.04163 | 0.04112 | 0.03472 | 0.04397 | 0.04154 | 0.04041 | 0.04166 | 0.03336 | 0.04350 | 0.00885 | 0.00000 | 0.03644 | 0.03191 | 0.01122 | 0.04289 | 0.04566 |
| **UBB** | 0.03977 | 0.00521 | 0.00709 | 0.00380 | 0.07931 | 0.00841 | 0.00844 | 0.03446 | 0.01136 | 0.00210 | 0.00990 | 0.03676 | 0.03644 | 0.00000 | 0.01191 | 0.03279 | 0.00778 | 0.00723 |
| **UNL** | 0.03659 | 0.01585 | 0.01734 | 0.01581 | 0.07969 | 0.01980 | 0.01790 | 0.02525 | 0.02030 | 0.01258 | 0.01993 | 0.03335 | 0.03191 | 0.01191 | 0.00000 | 0.02929 | 0.01890 | 0.02013 |
| **WOL** | 0.00471 | 0.03909 | 0.03841 | 0.03783 | 0.03098 | 0.04116 | 0.03816 | 0.03203 | 0.03902 | 0.03016 | 0.04017 | 0.00354 | 0.01122 | 0.03279 | 0.02929 | 0.00000 | 0.04014 | 0.04206 |
| **YRI** | 0.04624 | 0.00454 | 0.00375 | 0.00497 | 0.08066 | 0.00080 | 0.00581 | 0.04235 | 0.00589 | 0.00690 | 0.00395 | 0.04344 | 0.04289 | 0.00778 | 0.01890 | 0.04014 | 0.00000 | 0.00861 |
| **ZAM** | 0.04941 | 0.00618 | 0.01023 | 0.00599 | 0.08793 | 0.00903 | 0.01155 | 0.04339 | 0.01293 | 0.00773 | 0.01096 | 0.04621 | 0.04566 | 0.00723 | 0.02013 | 0.04206 | 0.00861 | 0.00000 |

**Table S5A**. Matrix of the weighted $F_{ST}$ statistic values between the TrypanoGEN, 1000 genomes and AGVP data sets

| | | AFR | AFR | AFR | AFR | AFR | AFR | AFR | AMR | AMR | AMR | AMR | EAS | EAS | EAS | EAS | EAS | EUR | EUR | EUR | EUR | EUR | SAS | SAS | SAS | SAS | SAS |
|---|---|---|---|---|---|---|---|---|---|---|---|---|---|---|---|---|---|---|---|---|---|---|---|---|---|---|---|
| Super | pop | ACB | ASW | ESN | GWD | LWK | MSL | YRI | CLM | MXL | PEL | PUR | CDX | CHB | CHS | JPT | KHV | CEU | FIN | GBR | IBS | TSI | BEB | GIH | ITU | PJL | STU |
| AFR | ACB | | 0.002 | 0.003 | 0.006 | 0.006 | 0.004 | 0.002 | 0.082 | 0.095 | 0.127 | 0.071 | 0.129 | 0.130 | 0.131 | 0.131 | 0.127 | 0.103 | 0.107 | 0.103 | 0.099 | 0.101 | 0.094 | 0.097 | 0.096 | 0.093 | 0.095 |
| AFR | ASW | 0.002 | | 0.009 | 0.010 | 0.009 | 0.010 | 0.008 | 0.064 | 0.078 | 0.110 | 0.053 | 0.116 | 0.116 | 0.117 | 0.117 | 0.113 | 0.085 | 0.088 | 0.085 | 0.081 | 0.083 | 0.077 | 0.080 | 0.079 | 0.076 | 0.079 |
| AFR | ESN | 0.003 | 0.009 | | 0.007 | 0.008 | 0.005 | 0.001 | 0.106 | 0.119 | 0.149 | 0.094 | 0.150 | 0.151 | 0.152 | 0.152 | 0.148 | 0.130 | 0.133 | 0.130 | 0.126 | 0.127 | 0.116 | 0.120 | 0.119 | 0.117 | 0.118 |
| AFR | GWD | 0.006 | 0.010 | 0.007 | | 0.011 | 0.004 | 0.006 | 0.101 | 0.114 | 0.143 | 0.089 | 0.145 | 0.146 | 0.147 | 0.147 | 0.143 | 0.124 | 0.127 | 0.124 | 0.120 | 0.121 | 0.112 | 0.115 | 0.114 | 0.112 | 0.113 |
| AFR | LWK | 0.006 | 0.009 | 0.008 | 0.011 | | 0.009 | 0.007 | 0.096 | 0.109 | 0.139 | 0.084 | 0.140 | 0.141 | 0.142 | 0.142 | 0.138 | 0.119 | 0.122 | 0.119 | 0.115 | 0.116 | 0.106 | 0.110 | 0.109 | 0.106 | 0.108 |
| AFR | MSL | 0.004 | 0.010 | 0.005 | 0.004 | 0.009 | | 0.004 | 0.106 | 0.119 | 0.149 | 0.094 | 0.151 | 0.152 | 0.154 | 0.153 | 0.149 | 0.130 | 0.134 | 0.130 | 0.126 | 0.128 | 0.117 | 0.121 | 0.119 | 0.117 | 0.119 |
| AFR | YRI | 0.002 | 0.008 | 0.001 | 0.006 | 0.007 | 0.004 | | 0.104 | 0.117 | 0.146 | 0.092 | 0.148 | 0.149 | 0.150 | 0.150 | 0.146 | 0.128 | 0.131 | 0.128 | 0.124 | 0.125 | 0.115 | 0.119 | 0.117 | 0.115 | 0.117 |
| AMR | CLM | 0.082 | 0.064 | 0.106 | 0.101 | 0.096 | 0.106 | 0.104 | | 0.009 | 0.035 | 0.005 | 0.068 | 0.064 | 0.067 | 0.066 | 0.064 | 0.014 | 0.017 | 0.014 | 0.013 | 0.014 | 0.026 | 0.026 | 0.029 | 0.022 | 0.029 |
| AMR | MXL | 0.095 | 0.078 | 0.119 | 0.114 | 0.109 | 0.119 | 0.117 | 0.009 | | 0.016 | 0.017 | 0.064 | 0.058 | 0.061 | 0.059 | 0.060 | 0.032 | 0.033 | 0.033 | 0.032 | 0.033 | 0.033 | 0.035 | 0.037 | 0.031 | 0.037 |
| AMR | PEL | 0.127 | 0.110 | 0.149 | 0.143 | 0.139 | 0.149 | 0.146 | 0.035 | 0.016 | | 0.051 | 0.079 | 0.072 | 0.075 | 0.073 | 0.075 | 0.077 | 0.074 | 0.077 | 0.078 | 0.078 | 0.063 | 0.068 | 0.068 | 0.065 | 0.068 |
| AMR | PUR | 0.071 | 0.053 | 0.094 | 0.089 | 0.084 | 0.094 | 0.092 | 0.005 | 0.017 | 0.051 | | 0.073 | 0.070 | 0.072 | 0.072 | 0.069 | 0.010 | 0.015 | 0.010 | 0.008 | 0.009 | 0.026 | 0.025 | 0.028 | 0.021 | 0.028 |
| EAS | CDX | 0.129 | 0.116 | 0.150 | 0.145 | 0.140 | 0.151 | 0.148 | 0.068 | 0.064 | 0.079 | 0.073 | | 0.008 | 0.005 | 0.016 | 0.002 | 0.094 | 0.089 | 0.094 | 0.093 | 0.093 | 0.050 | 0.066 | 0.062 | 0.063 | 0.060 |
| EAS | CHB | 0.130 | 0.116 | 0.151 | 0.146 | 0.141 | 0.152 | 0.149 | 0.064 | 0.058 | 0.072 | 0.070 | 0.008 | | 0.001 | 0.007 | 0.006 | 0.091 | 0.085 | 0.092 | 0.091 | 0.091 | 0.049 | 0.064 | 0.060 | 0.062 | 0.059 |
| EAS | CHS | 0.131 | 0.117 | 0.152 | 0.147 | 0.142 | 0.154 | 0.150 | 0.067 | 0.061 | 0.075 | 0.072 | 0.005 | 0.001 | | 0.008 | 0.003 | 0.093 | 0.088 | 0.094 | 0.093 | 0.093 | 0.050 | 0.065 | 0.062 | 0.063 | 0.060 |
| EAS | JPT | 0.131 | 0.117 | 0.152 | 0.147 | 0.142 | 0.153 | 0.150 | 0.066 | 0.059 | 0.073 | 0.072 | 0.016 | 0.007 | 0.008 | | 0.013 | 0.093 | 0.087 | 0.093 | 0.092 | 0.093 | 0.051 | 0.065 | 0.062 | 0.063 | 0.060 |
| EAS | KHV | 0.127 | 0.113 | 0.148 | 0.143 | 0.138 | 0.149 | 0.146 | 0.064 | 0.060 | 0.075 | 0.069 | 0.002 | 0.006 | 0.003 | 0.013 | | 0.090 | 0.085 | 0.090 | 0.089 | 0.089 | 0.047 | 0.062 | 0.058 | 0.059 | 0.056 |
| EUR | CEU | 0.103 | 0.085 | 0.130 | 0.124 | 0.119 | 0.130 | 0.128 | 0.014 | 0.032 | 0.077 | 0.010 | 0.094 | 0.091 | 0.093 | 0.093 | 0.090 | | 0.006 | 0.000 | 0.002 | 0.003 | 0.035 | 0.031 | 0.036 | 0.025 | 0.036 |
| EUR | FIN | 0.107 | 0.088 | 0.133 | 0.127 | 0.122 | 0.134 | 0.131 | 0.017 | 0.033 | 0.074 | 0.015 | 0.089 | 0.085 | 0.088 | 0.087 | 0.085 | 0.006 | | 0.007 | 0.010 | 0.011 | 0.035 | 0.032 | 0.037 | 0.027 | 0.037 |
| EUR | GBR | 0.103 | 0.085 | 0.130 | 0.124 | 0.119 | 0.130 | 0.128 | 0.014 | 0.033 | 0.077 | 0.010 | 0.094 | 0.092 | 0.094 | 0.093 | 0.090 | 0.000 | 0.007 | | 0.002 | 0.004 | 0.035 | 0.031 | 0.036 | 0.026 | 0.037 |
| EUR | IBS | 0.099 | 0.081 | 0.126 | 0.120 | 0.115 | 0.126 | 0.124 | 0.013 | 0.032 | 0.078 | 0.008 | 0.093 | 0.091 | 0.093 | 0.092 | 0.089 | 0.002 | 0.010 | 0.002 | | 0.002 | 0.035 | 0.031 | 0.036 | 0.026 | 0.036 |
| EUR | TSI | 0.101 | 0.083 | 0.127 | 0.121 | 0.116 | 0.128 | 0.125 | 0.014 | 0.033 | 0.078 | 0.009 | 0.093 | 0.091 | 0.093 | 0.093 | 0.089 | 0.003 | 0.011 | 0.004 | 0.002 | | 0.034 | 0.030 | 0.034 | 0.025 | 0.035 |
| SAS | BEB | 0.094 | 0.077 | 0.116 | 0.112 | 0.106 | 0.117 | 0.115 | 0.026 | 0.033 | 0.063 | 0.026 | 0.050 | 0.049 | 0.050 | 0.051 | 0.047 | 0.035 | 0.035 | 0.035 | 0.035 | 0.034 | | 0.004 | 0.002 | 0.003 | 0.002 |
| SAS | GIH | 0.097 | 0.080 | 0.120 | 0.115 | 0.110 | 0.121 | 0.119 | 0.026 | 0.035 | 0.068 | 0.025 | 0.066 | 0.064 | 0.065 | 0.065 | 0.062 | 0.031 | 0.032 | 0.031 | 0.031 | 0.030 | 0.004 | | 0.004 | 0.004 | 0.004 |
| SAS | ITU | 0.096 | 0.079 | 0.119 | 0.114 | 0.109 | 0.119 | 0.117 | 0.029 | 0.037 | 0.068 | 0.028 | 0.062 | 0.060 | 0.062 | 0.062 | 0.058 | 0.036 | 0.037 | 0.036 | 0.036 | 0.034 | 0.002 | 0.004 | | 0.003 | 0.001 |
| SAS | PJL | 0.093 | 0.076 | 0.117 | 0.112 | 0.106 | 0.117 | 0.115 | 0.022 | 0.031 | 0.065 | 0.021 | 0.063 | 0.062 | 0.063 | 0.063 | 0.059 | 0.025 | 0.027 | 0.026 | 0.026 | 0.025 | 0.003 | 0.004 | 0.003 | | 0.003 |
| SAS | STU | 0.095 | 0.079 | 0.118 | 0.113 | 0.108 | 0.119 | 0.117 | 0.029 | 0.037 | 0.068 | 0.028 | 0.060 | 0.059 | 0.060 | 0.060 | 0.056 | 0.036 | 0.037 | 0.037 | 0.036 | 0.035 | 0.002 | 0.004 | 0.001 | 0.003 | |

**Table S5B**. Matrix of the weighted $F_{ST}$ statistic values between the global 1000 genomes populations for comparison with African distances. The comparisons within super populations are highlighted in yellow and summarised in Table S5C below

| | Max Fst | Mean Fst |
|---|---|---|
| **Africa** | 0.0105 | 0.0063 |
| **Americas** | 0.0509 | 0.0222 |
| **East Asia** | 0.0160 | 0.0069 |
| **West Eurasia** | 0.0112 | 0.0047 |
| **South Asia** | 0.0043 | 0.0032 |

**Table S5C. Summary of weighted $F_{ST}$ statistic values within super populations of 1000 Genomes samples.** Note the high values for $F_{ST}$ within the Americas, which are presumably due to high levels of admixture. Although the values for Africa are similar to the values for the major Eurasian groups it should be remembered that the 1000 Genomes project only included samples from the Niger-Congo linguistic group and the other major linguistic groups were not represented.

## Supplemental Excel Spreadsheets

**Table S6.** snpEff classification of effect of SNP and its impact.

**Table S7. Genome wide distribution of extreme signatures of selection in the UNL**. Ensembl annotations of the coding and non-coding regions of the genome harbouring extreme iHS scores (positive iHS > +3.0, negative iHS < -3).

**Table S8. Protein coding genes under positive selection in the UNL**. A list of unique genes having extreme iHS scores (> +3.0) including those that intersect with the UBB population.

**Table S9. Top hits of significant genes in UNL**. Top hits of significant genes in UNL. Genes in the 1% of 100kb bins with highest frequencies of SNP with absolute iHS > 2.

**Table S10**. **Top hits of significant genes unique to the UNL**. Genes in top 1% of 100kb bins from table S9 that are only present in the UNL and not found in the UBB population.

**Table S11**. **Top hits of significant genes highly differentiated between the UNL and UBB**. Genes were ranked individually on the parameters of xpEHH [UNL-UBB], high Fst [UNL-UBB], and Tajima's D [UNL], and then a combined rank was obtained by summation of the individual ranks.
