# Supplementary material for: High Levels of Genetic Diversity within Nilo-Saharan Populations: Implications for Human Adaptation
Source: Am J Hum Genet. 2020 Aug 10;107(3):473–86. doi: 10.1016/j.ajhg.2020.07.007 (PMC7477016; doi:10.1016/j.ajhg.2020.07.007)
Supplement: Document S2. Article plus Supplemental Information [file mmc3.pdf]

High Levels of Genetic Diversity within Nilo-Saharan Populations: Implications for Human Adaptation

Julius Mulindwa,[1,2] Harry Noyes,[3] Hamidou Ilboudo,[4] Luca Pagani,[5,6] Oscar Nyangiri,[1] Magambo Phillip Kimuda,[1] Bernardin Ahouty,[7] Olivier Fataki Asina,[8] Elvis Ofon,[9] Kelita Kamoto,[10] Justin Windingoudi Kabore,[11,15] Mathurin Koffi,[7] Dieudonne Mumba Ngoyi,[8] Gustave Simo,[9] John Chisi,[10] Issa Sidibe,[11] John Enyaru,[2] Martin Simuunza,[12] Pius Alibu,[2] Vincent Jamonneau,[14] Mamadou Camara,[15] Andy Tait,[16] Neil Hall,[17] Bruno Bucheton,[14,15] Annette MacLeod,[16] Christiane Hertz-Fowler,[3] Enock Matovu,[1,*] and the TrypanoGEN Research Group of the H3Africa Consortium

Summary

Africa contains more human genetic variation than any other continent, but the majority of the population-scale analyses of the African peoples have focused on just two of the four major linguistic groups, the Niger-Congo and Afro-Asiatic, leaving the Nilo-Saharan and Khoisan populations under-represented. In order to assess genetic variation and signatures of selection within a Nilo-Saharan population and between the Nilo-Saharan and Niger-Congo and Afro-Asiatic, we sequenced 50 genomes from the Nilo-Saharan Lugbara population of North-West Uganda and 250 genomes from 6 previously unsequenced Niger-Congo populations. We compared these data to data from a further 16 Eurasian and African populations including the Gumuz, another putative Nilo-Saharan population from Ethiopia. Of the 21 million variants identified in the Nilo-Saharan population, 3.57 million (17%) were not represented in dbSNP and included predicted non-synonymous mutations with possible phenotypic effects. We found greater genetic differentiation between the Nilo-Saharan Lugbara and Gumuz populations than between any two Afro-Asiatic or Niger-Congo populations. F3 tests showed that Gumuz contributed a genetic component to most Niger-Congo B populations whereas Lugabara did not. We scanned the genomes of the Lugbara for evidence of selective sweeps. We found selective sweeps at four loci (*SLC24A5*, *SNX13*, *TYRP1*, and *UVRAG*) associated with skin pigmentation, three of which already have been reported to be under selection. These selective sweeps point toward adaptations to the intense UV radiation of the Sahel.

Introduction

The modern humans who migrated out of Africa in the last 100 ka came from only a subset of all African populations. The peoples who remained were more genetically diverse and have continued to diversify in response to changing environmental and disease pressures and admixture events.[1–6] African populations have also migrated and intermixed to create the rich mosaic of genetic and cultural variation that is found today.[7] The paucity of genetic, historical, and archaeological records has led to a heavy dependence on linguistic analysis for classification of African populations, and this strategy has identified four major African language families (Afro-Asiatic, Niger-Congo, Nilo-Saharan, and Khoisan) (Figure 1) and provided evidence for the migration of Bantu speakers out of the Nigeria-Cameroon border region into South and East Africa.[4] The advent of genetic analysis has generally supported the main population groups identified by linguistic analysis but has also revealed admixture between speakers of different language groups and language acquisitions from genetically unrelated groups.[4,6,9]

The Nilo-Saharan family comprises 206 languages spoken by 34 million people (1996 estimate) and is divided into approximately 12 subgroups.[10,11] This family is particularly problematic for linguists because there is only weak evidence for establishing the relationships between the subgroups and some authors treat Nilo-Saharan as a collection of isolated language groups rather than a single family.[11] Some smaller Nilo-Saharan groups (Gumuz, Koman,

[1]College of Veterinary Medicine, Animal Resources and Biosecurity, Makerere University, P.O. Box 7062, Kampala, Uganda; [2]College of Natural Sciences, Makerere University, P.O. Box 7062, Kampala, Uganda; [3]Centre for Genomic Research, University of Liverpool, Liverpool L69 7ZB, UK; [4]Institut de Recherche en Sciences de la Santeì (IRSS) - Uniteì de Recherche Clinique de Nanoro (URCN), Nanoro, Burkina-Faso; [5]Institute of Genomics, University of Tartu, 51010 Tartu, Estonia; [6]Department of Biology, University of Padova, Via U. Bassi, 58/B - 35121 Padova, Italy; [7]Université Jean Lorougnon Guédé (UJLoG) de Daloa, Côte d'Ivoire; [8]Institut National de Recherche Biomedicale, Avenue de la Democratie, Kinshasa Gombe, P.O. Box 1197 Kinshasa, Democratic Republic of Congo; [9]Faculty of Science, University of Dschang, P.O. Box 67, Dschang, Cameroon; [10]University of Malawi, College of Medicine, Department of Basic Medical Sciences, Private Bag 360, Chichiri, Blantyre 3, Malawi; [11]Institute, Centre International de Recherche-Développement sur l'Elevage en zones Subhumides (CIRDES), 01 BP 454 Bobo-Dioulasso 01, Burkina Faso; [12]Department of Disease Control, School of Veterinary Medicine, University of Zambia, P.O. Box 32379, Lusaka, Zambia; [14]Institut de Recherche pour le Développement (IRD), IRD-CIRAD 177, TA A-17/G, Campus International de Baillarguet, 34398 Montpellier, France; [15]Programme National de Lutte contre la Trypanosomose Humaine Africaine, BP 851, Conakry, Guinée; [16]Wellcome Centre for Integrative Parasitology, Biodiversity Animal Health and Comparative Medicine, Glasgow G61 1QH, UK; [17]Earlham Institute Norwich Research Park Innovation Centre, Colney Ln, Norwich NR4 7UZ, UK
*Correspondence: matovue@covab.mak.ac.ug
https://doi.org/10.1016/j.ajhg.2020.07.007.
© 2020 The Authors. This is an open access article under the CC BY license (http://creativecommons.org/licenses/by/4.0/).

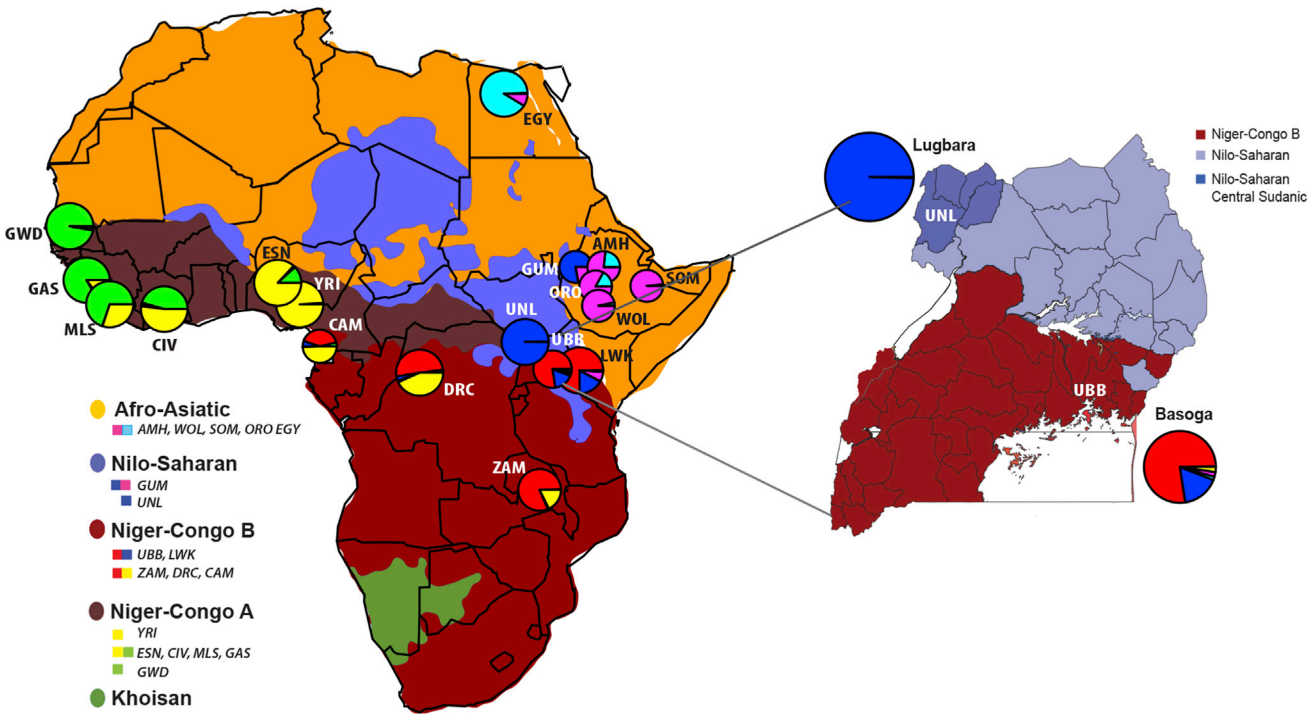

Figure 1. Map of Africa Showing the Distribution of Five Major African Linguistic Families, the Locations Where Samples Were Collected, and the Proportions of Different Genetic Components

The pie chart size is proportional to the sample size and pie chart proportions and colors correspond to the proportions and colors of ADMIXTURE components within that population for K = 6 (Figure 3). Note that the map colors for languages are not associated with pie chart colors. The legend shows first the map color for each major linguistic group and second the major colors (>25% admixture component) of the admixture pie charts for each population in that linguistic group. The linguistic distribution map was compiled from data in Ethnologue and used under the Creative Commons Attribution-ShareAlike 4.0 International License. Our populations were sampled from Guinea (GUI), Côte d'Ivoire (CIV), Cameroon (CAM), Democratic Republic of Congo (DRC), Zambia (ZAM), and Uganda (UNL & UBB), the 1000 Genomes project (Gambia [GWD], Sierra-Leone [MSL], Nigeria [ESN, YRI], Kenya [LWK], Egypt [EGY]), and the African Genome Variation project (Ethiopia [AMH, GUM, ORO, SOM, WOL]). The inset map shows sampling sites in Uganda. The Lugbara (UNL) were from West Nile region that is predominantly occupied by Nilo-Saharan speakers and the Basoga (UBB) were from the southern region, which is occupied by Bantu speaking people. This map was overlaid with pie charts derived from the admixture plot using R tools. The Ugandan map was generated using QGIS3.6 (see Web Resources) with regional ethnicity classification traced with inference from "Ethnologue languages of Uganda."[8]

Kadu, Chabu) have been excluded from the Nilo-Saharan family by some authors or treated as early branching distantly related groups by others.[10,12] Genetic data can be used to show how linguistic groups map onto genetically defined human populations.[4] However, genomes have been sequenced from fewer than 100 of the 2,139 African linguistic groups recognized by Ethnologue.[6,13–16] Here we have sequenced the genomes of 50 individuals from the Nilo-Saharan Lugbara population of Northwestern Uganda. The Gumuz is the only other Nilo-Saharan population to be sequenced at this scale and the linguistic evidence for its inclusion in the Nilo-Saharan family is debated.[10,12] For comparison we also sequenced the genomes of 250 individuals from 6 new Niger-Congo populations from Guinea, Côte d'Ivoire, Cameroon, Democratic Republic of Congo, Zambia, and Uganda and also included published data from 13 additional African populations from the 1000 Genomes and African Genome Variation Projects.[2,17] We show that the Lugbara are genetically distinct from all Niger-Congo and Afro-Asiatic populations and from the Gumuz.[2,5] Through this level of sequencing, we have been able to use the major methods for identification of loci under selection, iHS and xpEHH, which require at least 15 genomes to achieve 80% power.[18] To date, this number of samples has only been sequenced from 7 Niger-Congo, 6 Afro-Asiatic, and a single putative Nilo-Saharan population (Gumuz).[2,16,19] Analyses of Niger-Congo genomes have already identified loci associated with resistance to malaria and human african trypanosomiasis (HAT).[20,21] In the Lugbara we found loci under selection associated with skin pigmentation and hair formation.

Subjects and Methods

Study Samples

The samples used for this study were obtained from the TrypanoGEN biobank,[22] the numbers and ethnic groups of the samples from each country are shown in Table S1. Groups of samples that cluster together on the MDS plot and appear similar on the Admixture plots are referred to by the name of the linguistic group unless there were multiple linguistic groups within a cluster, in which case

they are referred to by the country name or abbreviation (Table S1). Ethical approval for the study was provided by the ethics committees of each TrypanoGEN consortium member: Uganda (Vector Control Division Research Ethics Committee (Ministry of Health), Uganda National Council for Science and TechnologyHS 1344), Zambia (The University of Zambia Biomedical Research Ethics Committee: 011-09-13), Democratic Republic of Congo (Minister de la Sante Publique: No 1/2013), Cameroon (Le Comite National d'Ethique de la Recherche pour la Sante Humain: 2013/364/L/CNERSH/SP), Côte d'Ivoıre (Ministere de la Sante et de la Lutte Contre le SIDA, Comite National D'Ethique et de la Recherche 2014/No 38/MSLS/CNER-dkn), and Guinea (Comite Consultatif de Deontologie et d'Ethique [CCDE] de l'Institut de Recherche pour le Developpement: 1-22/04/2013). All the participants in the study were guided through the consent forms, and written consent was obtained to collect biological specimens. Study participants provided informed consent for sharing and publishing their anonymized data.

Peripheral blood was collected from the participants at the field sites, frozen, and transported to reference laboratories. DNA was extracted using the whole blood MidiKit (QIAGEN). The DNA was quantified using the Qubit (QIAGEN) and approximately 1 μg was used for sequencing at the University of Liverpool, UK. DNA from Cameroon and Zambia was sequenced at Baylor College, USA.

Sequencing and SNP calling

300 participants' DNA samples (Lugbara [UNL], 50; Basoga [UBB], 33; Zambia [ZAM], 41; Democratic Republic of Congo [DRC], 50; Cameroon [CAM], 26; Côte d'Ivoire [CIV], 50; Guinea [GAS], 50) were selected and subjected to whole-genome sequencing (Table S1). The whole-genome sequencing libraries of samples from Guinea, Côte d'Ivoire, Uganda, and DRC were prepared using the Illumina Truseq PCR-free kit and sequenced on the Illumina Hiseq2500 to 10× coverage at the Centre for Genomic Research (University of Liverpool). The samples from Zambia and Cameroon were sequenced on an Illumina X Ten system to 30× at the Baylor College of Medicine Human Genome Sequencing Centre. The sequenced reads were mapped onto the human_g1k_v37_decoy reference genome using BWA.[23] The SNP calling on all the samples was carried out using the genome analysis tool kit GATK v3.4[24] to create a GVCF file for each individual. GVCF files were then merged to create a combined VCF file also using GATK. SnpEff was used for variant annotation.[24] An analysis of copy number variation has been published separately.[25]

From the 1000 Genomes project[16] we obtained variant call files of 50 samples from each of the Esan and Yoruba from Nigeria; Mende from Sierra Leone; Gambian from Western Division of The Gambia; Luhya from Western Kenya; five samples from each of five populations of West Eurasian origin: Utah residents with northern and western European ancestry, Finnish from Finland, British in England and Scotland, Iberian from Spain, Toscani from Italy.

From the African Genome Variation Project[2,26] we extracted 50 Egyptian genome sequences and 24 from each of the following Ethiopian populations: Amhara, Ethiopian Somali, Oromo, Wolayta, and Gumuz. The African Genome Variation datasets were obtained from European Genome-Phenome Archive,[27] EGA: EGAD00001000598, EGA: EGAD00001003296, EGA: EGAD00010001221, under the terms of the Wellcome Sanger Institute (WSI) data access agreement.

Data Quality Control and Filtering

The data were filtered to minimize batch effects potentially introduced by the presence of samples sequenced at different depths by different labs. For descriptive statistics of the TrypanoGEN dataset all loci were retained. For all other analyses, sites that met any of the following criteria were removed; missing data $>$ 10%, loci with $<$ 3 SNP calls, minor allele frequency (MAF) $<$ 0.01, Hardy-Weinberg equilibrium $p < 0.001$. For population analyses, the remaining SNP loci were thinned in order to retain only loci with $r^2 < 0.1$. Individuals with $>$10% missing data were also removed. Data were phased with Shapeit2 v2.r837,[28] which also imputed missing data, prior to combining our data with genomes from the 1000 genomes and African Genome Variation projects using BCFtools (v.1.6),[27] retaining only loci that were present in all datasets.

For signatures of selection, the filtered and phased variant call format files were further filtered using VCFtools v.0.1.16[29] to remove loci with MAF $<$ 0.05.

Multidimensional Scaling Analysis

To infer the population structure based on the underlying genetic variation among the populations, we carried out multidimensional scaling (MDS) using PLINK 1.9[30] and plotted MDS coordinates using R v.3.2.1.[31] The MDS was carried out on our sequence data, which was merged with a maximum of 50 samples from each of the 13 additional populations from Africa and Europe from the 1000 Genomes project[16] and the African Genome Variation project.[2,26]

Population Admixture

Admixture was tested for 1 to 9 genetic components (K) using ADMIXTURE 1.23[32] with 3 replicate runs for each value of K.

All plausible pairs of available populations that might be sources of the selected East African Populations (UNL, UBB, LWK, GUM, AMH) were tested for evidence of contribution to those populations using the F3 test in AdmixTools[33] and implemented in R using *admixr*.[34]

Allele Frequency Statistics: In-breeding Coefficient, Tajima D, F_{ST}

We followed the workflow of Cadzow et al. for allele frequency statistics.[35] To determine the extent of inbreeding within each of our populations, we measured the inbreeding coefficient, F,[36] using VCFtools (v.0.01.14).[29] The Tajima D statistic[37] was used to identify regions that did not fit the neutral model of genetic drift and mutation in bins of 3 kb also in VCFtools. The level of population differentiation was estimated with Wright's F_{ST}[38] in PLINK v.1.9. The pairwise F_{ST} matrix was generated between our sequence data, 1000 Genome project,[16] and the African Genome Variation Project populations.[2,26]

Signatures of Selection

The sequence data were scanned for regions that might be under selection using the Extended Haplotype Homozygosity (EHH) test within and between populations.[39] The SNP were phased using SHAPEIT v.2.2,[28] and the R software package *rehh3*[40] was used to calculate two EHH derived statistics: the intra-population integrated Haplotype Score (iHS)[41] and inter-population xpEHH score,[42] that identify SNPs that are under selection in one population but not in another. Only SNPs with a MAF $>$ 0.05 were included in the analysis. We used the method of Voight et al. to

identify the regions of the genome under the strongest selection pressure;[41] the genome was divided into 100 kb bins and the fraction of SNP with iHS > 2 in each bin was obtained. Bins with <20 SNP were disregarded. The 1% of bins with the highest fraction of SNP with absolute iHS > 2 were considered to be significant.[41] Bins were annotated with the lists of genes that they contained using Biomart. Different types of evidence for signatures of selection were combined using Bedtools v.2.26.0[43] to identify the intersection of the iHS, with xpEHH and the allele frequency-based statistics of F_{ST} and Tajima D.

Results

We sequenced the genomes of 50 individuals from the Nilo-Saharan Lugbara population and 250 from 17 linguistic groups from Guinea, Côte d'Ivoire, Cameroon, Democratic Republic of Congo, Uganda, and Zambia (Tables S1 and S2).

The samples from Zambia and Cameroon were sequenced to 30× coverage while other populations were sequenced to 10× coverage. The call rate was 97.4% in the 10× samples and 99.4% in the 30× samples. The 30×-sequenced samples had higher proportions of heterozygotes (9.3%) compared with the 10× sequenced samples (7.5%) and there was a concomitant higher frequency of low Hardy-Weinberg p values in the 10× data (Figure S1). There were 38,963,563 raw variants, filtering removed fourteen individuals and 23,017,723 loci leaving 286 samples and 15,945,844 variant loci that were available for population and signatures of selection analyses. Table S3 shows the number of loci removed by each filtering step, most variants were removed from the analysis because of low count or frequency of minor alleles (21,604,569 MAF < 1% or minor allele count ≤ 2). The mean call rate after filtering was 99.2% for the 10× samples and 99.95% for the 30× samples. The data were phased with Shapeit2, which imputed genotypes at the small number of remaining missing loci. The commonest form of bias in low-coverage data is an excess of singleton variant loci[44] and these were removed by the filtering strategy (Figure S1).

The Nilo-Saharan Lugbara Population Has a High Proportion of Novel Variation

We observed little evidence of inbreeding within the populations; the majority of the individuals had an inbreeding coefficient (F) of less than 0.1 (Figure S2). We classified variants as known if they were present in dbSNP build 150 (20/11/2019) and novel if not. We identified approximately 22 million variant loci in the Lugbara population (Table S4, Figure S3). The frequencies of known and novel variants were similar in all the six Niger-Congo populations (12.9% novel, SE 0.003); however, the Nilo-Saharan Lugbara population from North West Uganda had significantly more novel SNPs (17.1% $p < 0.001$) (Figure S3C), presumably due to an under-representation of Nilo-Saharan populations in previous genomic studies. We assessed the impacts of the variants on function using snpEff; 99% of SNP were classified as "modifier," and these were mainly intergenic; the remaining 1% of SNPs had more informative classifications: low, moderate, or high impact (Table S4, Figures S3B and S3C). Of the 1% of SNP with informative classifications (low, moderate, or high impact), nearly 90% were predicted to have moderate impact in both known and novel variants. The frequency of high-impact variants was twice as high in the novel variants as it was among the known variants (6.3% *cf.* 3.0%). There was a larger proportion of rare alleles (MAF < 5%) in the set of novel SNPs than in the known SNPs (Figure S4), as expected for SNPs that are unique to a specific population or geographic region.

The Nilo-Saharan Lugbara Population Is Distinct from Other African Populations

Bi-allelic loci from the 286 TrypanoGEN samples were merged with 1,000 Genomes and African Genome Variation Project data to obtain 10,857,449 loci that were present in all three datasets for population analysis. These were filtered to remove linked loci ($r^2 > 0.1$) yielding a final dataset of 1,465,578 SNP and 731 samples that were used for MDS, Admixture, and F3 analysis.

Multidimensional scaling analysis (Figure 2) showed that samples formed tight geographic groups irrespective of data source or sequence coverage. The exception was the Nilo-Saharan Lugbara population from North West Uganda, which was distinct from both the Nilo-Saharan Gumuz of Ethiopia and the Basoga from southeast Uganda. The two Nilo-Saharan populations were well separated from each other and from the East African Niger-Congo B and the Ethiopian Afro-Asiatic populations. Even when combined with a West Eurasian dataset (Figure S5B), the two putative Nilo-Saharan populations (Lugbara and Gumuz) appeared as divergent from each other as Niger-Congo-A and Niger-Congo-B populations from East and West Africa. This demonstrates that the focus on genetics of Niger-Congo and Afro-Asiatic populations has led to the neglect of the greater diversity within other African populations.

The Nilo-Saharan Lugbara Show Low Genetic Admixture and High Genetic Distance from Other African Populations

We then used Admixture to analyze the population structure of the same 731 samples used for the MDS analysis. The admixture coefficients of variation were very similar (0.262–0.271) for all numbers of genetic components (K3-9) (Figure S6). Although caution should be used when interpreting Admixture clusters as broad genetic components,[45] remarkably at all values of K except $K = 7$ Gumuz and Lugbara shared a single large component, which was also important in Afro-Asiatic samples (at $K \leq 5$) and to a lesser extent in East African Niger Congo B samples (LWK, UBB) (Figure 3).

With $K > 5$ the Niger-Congo populations separated into an east African cluster of the Ugandan Basoga and Kenyan

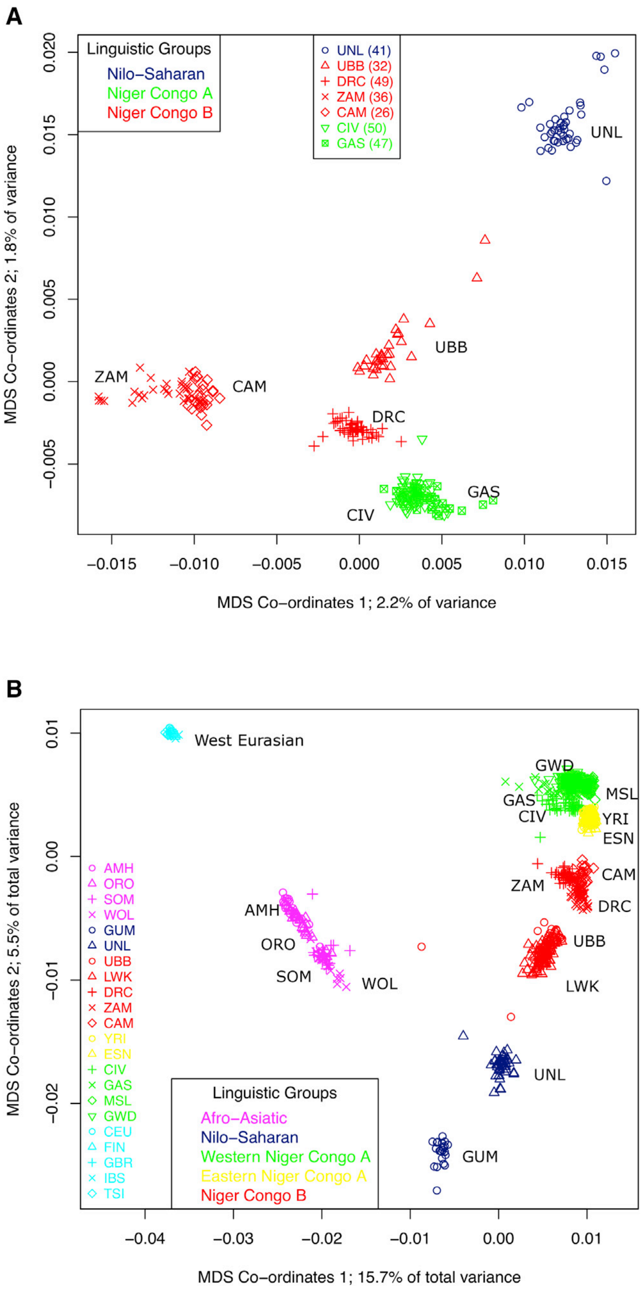

Figure 2. Multidimensional Scaling Analysis of Sequenced Populations

(A) This study: Guinea (GAS), Côte d'Ivoire (CIV), Cameroon (CAM), Democratic Republic of Congo (DRC), Uganda (Nilotics, UNL, Niger Congo B, UBB), and Zambia (ZAM); seven Soli/Chikunda (Niger-Congo B)-speaking individuals were outliers by MDS and are not shown in this plot but are shown in Figure S5A.
(B) This study and African Genome Variation Project Ethiopian samples Amhara (AMH), Welayta (WOL), Oromo (ORO), Ethiopian Somali (SOM), and Gumuz (GUM) and 50 samples from each 1000 Genomes African population Nigeria (ESN, YRI), Gambia (GWD), Mende Sierra Leone (MSL), Kenya (LWK). Colors for each cluster are taken from the color for the dominant genetic component for that cluster in the admixture plot at K = 6.

Luhya, a central African cluster of the Zambia, Cameroon, and Democratic Republic of Congo, a Nigerian cluster of the Esan and Yoruba, and a far west-African cluster of the Côte d'Ivoire, Sierra Leone, Guinea, and Gambia populations. We also observed at $K \geq 8$ a homogeneous group of seven Soli/Chikunda (Niger-Congo B)-speaking individuals within the Zambia population with no admixture with other populations and who were also outliers on the MDS coordinates plot (Figure S5A), the source of this divergent ancestry is unknown.

F3 Tests of Admixture Hypotheses

The admixture hypotheses generated by Admixture were tested with the three populations (F3) test implemented with AdmixTools.[33] All possible pairs of 2 West Eurasian (TSI, EGY) and 17 African populations (AMH, ORO, SOM, WOL, DRC, CAM, ZAM, ESN, YRI, GWD, MSL, GUI, CIV, LWK, UBB, GUM, UNL) were tested as possible sources of five East African populations (Afro-Asiatic AMH; Nilo-Saharan GUM and UNL; East African Niger-Congo B UBB and LWK) (Figures 4 and S8).

Pairs of each African population and each West Eurasian population were plausible sources to the Amhara (AMH) population consistent with the Admixture plot which suggests that the Afro-Asiatic populations have a large West Eurasian admixture component as previously reported (Figure S8).

No pairs of populations were jointly source to either of the Nilo-Saharan populations (UNL and GUM) (Figure S8). However, the Gumuz and Lugbara had very different contributions to the ancestry of the Kenyan Luhya (Figure 4), despite sharing apparently similar ancestral components in the Admixture plot (Figure 3). There was evidence that both the Gumuz and Afro-Asiatic populations were plausible sources to the Luhya when paired with most African populations (Zscore < -16 for pairings with Zambia). In contrast there was very little evidence of ancestry from the Lugbara, which were only compatible with the Zambian population as plausible admixture sources, and even there the signal was much weaker (Z score $= -2.7$). The Gumuz but not the Lugbara also contributed to the Ugandan Basoga ancestry (Figure 4) but only when paired with the Zambian population.

These observations are most consistent with the population structure indicated in the Admixture plot at $K = 6$. At $K = 6$ the dominant ancestry component in Lugbara and Gumuz (dark blue in Figure 3) is also shared with the Luhya and Basoga, but this is not consistent with the F3 data. However, a minor component of the Gumuz (pink at $K = 6$), which is not observed in the Lugbara, is also shared with Luhya and Basoga and this is consistent with F3 data, which shows a Gumuz but not Lugbara contribution to these populations. The pink perhaps represents a pre-Bantu expansion East African population that has contributed to the Gumuz, Luhya and Basoga genomes but not the Lugbara.

We obtained pairwise F_{ST} distances between the Ugandan Lugbara and the other African populations to determine the genetic distance between them (Table S5, Figure S7). F_{ST} was relatively high (mean $F_{ST} > 0.015$)

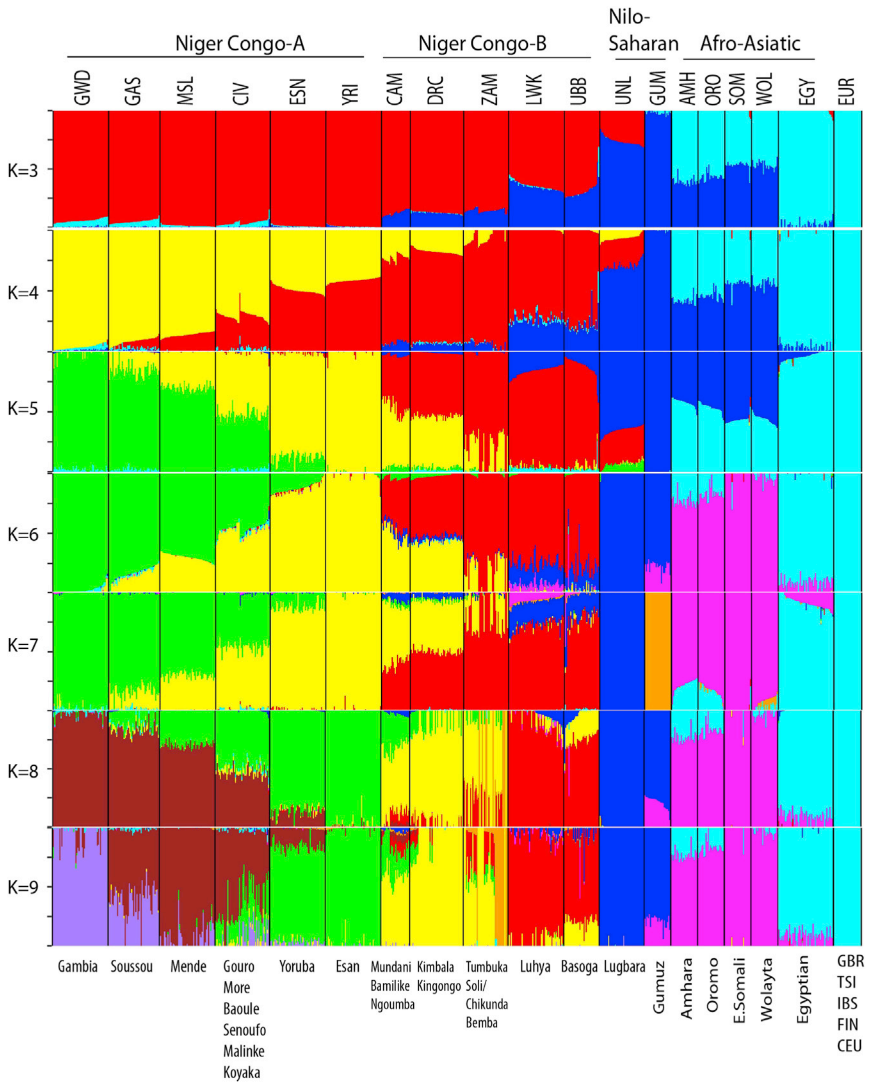

Figure 3. Genetic Admixture and Differentiation in Our Data, Selected 1000 Genomes, and AGVP Populations
Admixture plot (731 samples) for K = 3 to K = 9. Genome sequences from this study, 1000 Genomes African samples, AGVP Egyptian, Ethiopian, and European populations (GBR, British from England and Scotland; TSI, Toscani in Italy; IBS, Iberian in Spain; FIN, Finnish in Finland; CEU, Utah residents with Northern and Western European ancestry). Three replicates were carried out for each value of K.

between the Nilo-Saharan Lugbara samples and the Niger-Congo populations, except for the Uganda Basoga population (mean $F_{ST} = 0.011$) and Kenyan Luhya population (mean $F_{ST} = 0.012$). The Lugbara and Gumuz populations are about 1,000 km apart compared with the approximately 4,000 km, which separates the West and East African Niger-Congo A and B populations. However, F_{ST} between Niger-Congo A and B (0.008) was lower than between Lugbara and Gumuz ($F_{ST} = 0.025$, Table S5), indicating that Lugbara and Gumuz populations have very different histories.

Signatures of Selection in Nilo-Saharan Lugbara

Given the relative genetic isolation of the Nilo-Saharan Lugbara, we hypothesized that they could have unique genetic adaptations to their environment. We sought to identify those regions of the genomes that were under selection, using the linkage disequilibrium-based models of extended haplotype homozygosity (EHH). Those alleles with extreme EHH were then validated using the allele frequency-based F_{ST} statistic and Tajima's D. Of the 15,945,844 variant loci that passed QC, only those with MAF > 5% were retained for these analyses, a total of 8,882,525 in the Lugbara and 9,107,514 in the Basoga.

Signatures of Selection in the Lugbara and Basoga Populations

We compared the regions under selection within the Lugbara and Basoga populations. The Basoga population was selected due to their geographic proximity to the Lugbara (500 km) (Figure 1), the minimally shared genetic ancestry between these two Ugandan populations (Figure 3), and because the Ugandan Basoga can act as representatives of Niger-Congo B populations. Using the phased haplotype dataset of the Lugbara and Basoga populations, the EHH derived integrated haplotype score (iHS) values were calculated using the *rehh3* software for which we observed a normal distribution of the absolute iHS values (Figure S9). The Manhattan plot (Figure 5) shows 12 regions with extreme iHS ($|iHS| > 6$). However, there were protein-coding genes within 100 kb of only two of these peaks (*ROCK1*, *DCUN1D4*). Both genes are involved in diverse ranges of intracellular activities making it difficult to predict a specific effect on phenotype.[46,47] We therefore calculated the frequency of SNP with $|iHS| > 2$ in 100 kb bins[41] to identify the regions with greatest evidence of selection and that might contain genes associated with known phenotypes (Table S9). The *HLA* region had some of the highest frequencies of SNP with $|iHS| > 2$ as well as some of the highest values of iHS (> 6) and has been found to have signatures of selection previously.[48] A list of genes that are under selection and are also shared between the UNL and UBB populations is shown in Table 1.

Signatures of Selection in the Lugbara but Not Basoga Populations

In order to identify SNPs associated with adaptation in the Lugbara population, we identified those selective sweeps in

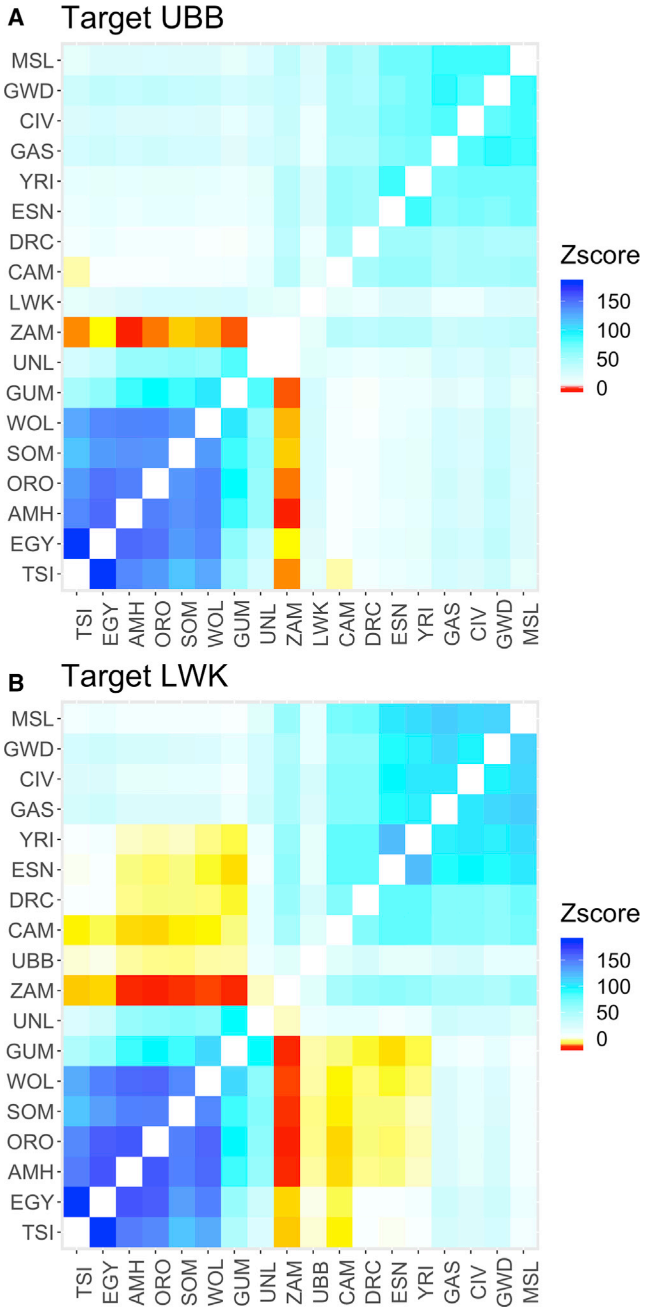

Figure 4. F3 Tests of Admixture
(A) Target UBB; Z scores for probability that a pair of populations contributed ancestry to the Uganda Niger Congo B Basoga.
(B) Target LWK; Z scores for probability that a pair of populations contributed ancestry to Kenyan Luhya.
Heatmap color represents intensity of Z score for probability that a population contributes genetic components to the target. Negative Z scores (yellow to red) are associated with increasingly strong evidence of a contribution and positive scores (cyan to blue) are associated with increasingly strong evidence against a contribution. White squares are inconclusive.

which the signature allele has achieved fixation in the Lugbara population but remains polymorphic in the Basoga population.[69] We first identified loci within the Lugbara population that had extreme iHS values and occurred at a high frequency within a 100 kb window (SNPs having iHS > 2.0

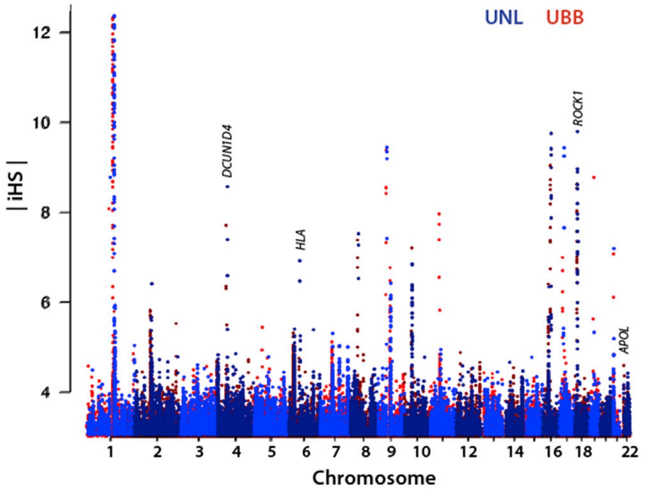

Figure 5. Genome-wide Signatures of Selection in the Lugbara and Basoga
Manhattan plot showing SNPs with extreme absolute iHS values (|iHS| > 3.0) that occur in the Lugbara (UNL blue) and Basoga (UBB red) populations.

and count > 20, Table S9). We then identified those that occur only in the UNL population (Table S10). Finally, we identified those genes with extreme iHS that are highly differentiated between the Lugbara and Basoga populations using high F_{ST} (top 5% quantile), high Tajima's D, and high cross population EHH (xpEHH > 2.5). The three different metrics were combined by ranking genes on each individual metric and then obtaining the sum of the ranks for each gene (Table S11). From this we identified a set of top ranked genes (Table 2) which were highly differentiated between the Lugbara (UNL) and Basoga (UBB) populations. The three highest ranked genes were *NEK4,* which is associated with schizophrenia,[70] *COLQ*, which is most highly expressed in CD8 T cells and CD56 NK cells,[71,72] and *UVRAG*, which is involved in melanosome biogenesis and skin pigmentation[73] and protection against UV radiation (Figure 6).

Discussion

SNP Discovery

Africa has the most genetically diverse populations on earth but while there are projects to sequence in excess of 100,000 genomes from populations in Europe,[74] Asia,[75] and the Americas[76] the 1000 Genomes Project is still the single largest dataset for Africa with 661 genome sequences. Not only do African genomes have a greater density of polymorphisms than genomes elsewhere, they also frequently have shorter haplotypes, which require a greater density of markers to phase accurately.[77] To date, most African genome-wide association studies (GWASs) have been undertaken using chips designed for West Eurasian populations. This can severely limit researchers' power to discover loci controlling disease. For example, a GWAS to identify loci regulating severe malaria failed to recapture the sickle cell locus because of limited linkage

Table 1. The Top 20% of Protein-Coding Genes with Strongest Signatures of Selection in the Lugbara Population

Chr	Associated Protein-Coding Gene	Associated Effect	Ref.
1	BX842679.1, **LYPD8, SDHC, C1orf192, NBPF20, PRDM2, SLC9A1, FAM46B**, GFI1[a], GPR89A, **PRPF3, ITLN2, F11R**, NBPF14, DESI2, **PRMT6, FLG[b], XCL2**, CENPL, **FGGY, PRAMEF10, NR0B2, C1orf172**, RIMKLA, **PPIAL4G**, C1orf159, **CD48**	[a]myeloid leukemia, [b]atopic dermatitis	49,50
2	**IRS1[C]**, RGPD5, **PARD3B**, PFN4, TP53I3, DYNC1I2, **CH17-132F21.1, C2orf47, SPATS2L, ZNF2**, ARHGAP15, VPS54, **AC017081.1, RAB3GAP1, MAP3K19**, ST3GAL5, **RFTN2**, ASXL2, GALNT14, **AMER3, PROKR1**	[c]diabetes	51
3	HACL1, **C3orf67**, LRRIQ4, **FXR1, TMEM45A, TOP2B**, ALCAM, **IQCB1, GOLGB1**, TF[d], FAM162A, WDR5B, ABCF3, VWA5B2, RPL24, IQCF3, HTR3E, ACTRT3, FILIP1L, SPSB4, MYNN, COLQ, **ABHD14A-ACY1**, NEK4, EIF5A2, RPL22L1, **CAMK2N2, PSMD2, KCNH8**, SFMBT1, TMEM110	[d]anemia	52
4	**ABCG2**, DCAF4L1, TMEM33, KLHL8, USP46, **ERVMER34-1**, PAICS, **C4orf33**, STATH, RXFP1, TECRL, ENPP6, STOX2, ANTXR2, **KLHL2**, HTN1, HTN3, **SCLT1, EIF4E, NDST3[e]**, C4orf46	[e]schizophrenia	53
5	NR2F1, PARP8, **TMEM232, PRELID2, JAKMIP2, PJA2, RP11-1026M7.2**, IL9, SLC25A48, **TIMD4[f], FAM153B**, NNT, **RBM27, PLAC8L1**, SDHA, **MYO10, TTC1, SKP1**, MED7, FAM71B, ITK[g], TGFBI	[f]tuberculosis, [g]HIV	54,55
6	**SAMD3, TMEM200A, UNC5CL, IPCEF1, OPRM1, EPHA7**, PKIB, DDO, METTL24, **TULP4**, ID4, **HLA-DQB1[h], HLA-DQA1**, BAI3, COX6A1P2, FGD2, SOX4, MYLK4, WRNIP1, **GRIK2**	[h]HIV, [h]tuberculosis, [h]diabetes	56–58
7	IGF2BP3, **MUC12**, MUC3A, **NAMPT, AOC1, KCNH2, C7orf62, AC006967.1, RBM48, GATS, PVRIG, GNA12, POM121L12, OR9A2[i], KEL, CARD11**, TRPV5, **AZGP1**, THSD7A, ZNF680, AGR2, **CDK6**, SERPINE1, ISPD	[i]odor perception	59
8	**FAM83A, PRR23D1, LRLE1, ZNF696, STC1**, SFRP1, ADCY8, **CSMD1, SDR16C5**, ZNF705G, **DDHD2, PPAPDC1B**, PBK, CLN8, **COPS5**		
9	**AL953854.2, BX255923.1, CR769776.1, TPRN[j], SSNA1, CBWD5, AL591479.1, CBWD7**, PHF2, **C9orf85, BX649567.1**, TRMT10B, GRIN1, **BRINP1, RP11-195B21.3**, AL365202.1, **INPP5E**	[j]deafness	60
10	BLNK, **ZNF37A, FAM21C, AL591684.1, PLEKHS1, CDNF[K]**, SORCS1, **A1CF, ASAH2B, DNAJB12, LARP4B**, MALRD1, BLOC1S2, PKD2L1, ANKRD2, UBTD1, **ADAM12, AFAP1L2, FANK1, KNDC1**, UTF1, **MTRNR2L7, C10ORF68**	[k]stroke	61
11	**SPATA19**, MRVI1, **DPP3, CTD-3074O7.11, MOGAT2, ANO3, FAM86C1, TREH**, DDX6, PGAP2, FADS3, AL356215.1, **UBASH3B**, UVRAG[l], **IFT46**	[l]autophagy	62
12	**SDR9C7, GALNT9[m]**, MGAT4C, **NTS, SCYL2[n]**, KCNJ8, **AC073528.1**, PRPH, TROAP, CLEC6A, **LRIG3, TMTC2**, HECTD4, SMCO2, AEBP2, **LGR5**, GAS2L3, CIT, C12orf56, ANO6, **CCDC59**	[m]neuralblastoma [n]arthrogryposis	63,64

(*Continued on next page*)

Table 1. *Continued*

Chr	Associated Protein-Coding Gene	Associated Effect	Ref.
13	***SLC15A1, DOCK9, THSD1, GPC5, HNRNPA1L2, C1QTNF9B, SPRY2, CKAP2***, *RFC3*, ***RGCC, VWA8***, *DZIP1*		
14	***PPP2R5C***, *DCAF5*, ***SERPINA6, RP11-796G6.2***, *TEX22*, ***EGLN3, NPAS3***		
15	***NDNL2, LMAN1L, FAM219B, MPI***, *PGPEP1L, CERS3*[o], *CKMT1A, CSK*[p], *CYP1A2*, ***CORO2B, ITGA11***, *RAB11A*, ***NEDD4, C2CD4A, FGF7, HDC, C15orf60***, *DUOX2*, ***CPLX3***, *BLM*, ***HCN4***	[o]ichthyosis, [p]SLE	65,66
16	***OTOA***, *METTL22, TMEM114, CBLN1*, ***USP10, KLHL36, PDILT, UMOD***[q], *RP11-20I23.1, GCSH*, ***CTD-2144E22.5***, *NKD1*	[q]kidney disease	67
17	***KRTAP4-4, PIK3R5, PIK3R6, MEOX1, MAP2K3, KCNJ12, SLC47A2, LGALS3BP, FLJ45079***, *NLK, KRT37, KRT38, C17orf82, TBX4*, ***NARF, CLEC10A, ASGR2, IKZF3***, *AC132872.2*, ***ZNF18, ENGASE***, *C1QTNF1, FAM211A, ZNF287*		
18	*ARHGAP28*, ***SLC14A2, MAPRE2***, *DSEL*, ***KIAA1468, PIGN***		
19	***TRPM4, RFX1, RLN3***, *PSG1*, ***ZNF600***, *ZNF28*, ***NOSIP, RCN3***, *NFKBID*, ***ARRDC2***, *DNMT1, EIF3G*, ***CATSPERG***, *AP3D1, DOT1L*, ***ECSIT***, *MIER2, AC018755.1, PLEKHJ1, TSHZ3*		
20	***RIMS4, CPNE1, RP1-309K20.6, WFDC12, FAM182B, ROMO1, NFS1, SPINT4***, *C20orf166, KCNB1, PTGIS*, ***DLGAP4***, *AAR2*, ***CST7, SLPI, MATN4***, *ARFGEF2*, ***ZSWIM3, ZSWIM1***, *PANK2*		
21	*TPTE*		
22	*KIAA1644, RP1-32I10.10*, ***CHEK2, TTC38***, *FAM118A, SMC1B, LDOC1L*, ***USP41, APOL4***[r], ***APOL2***[r], *TUBA8, USP18, POLR2F*, ***MICALL1, EIF3L***	[r]pathogen immunity	68

Genes are extracted from the protein coding genes in the top 1% of 100 kb iHS Windows (Table S8) with each gene having a mean iHS > 3.0 in the Lugbara population. The genes in bold are those that also have evidence of selection in the Basoga population. Genes with superscripts are those that are associated with the phenotype in the "Associated Effect" Column.

between markers and the functional SNP.[78] Our sequence data from six Niger-Congo populations and the Nilo-Saharan Lugbara have already contributed to the development of an Illumina Omni chip that is enriched for African SNPs and should reduce the number of important loci missed by GWASs in African populations.[79]

Demographic Inference

In this study, we carried out whole-genome sequencing on populations from six different sub-Saharan African countries, and combined our data with genome sequences from the 1000 Genomes and African Genome Variation projects to better understand the relationship of the Lugbara to neighboring populations. The great diversity of Nilo-Saharan languages meant that they were recognized as belonging to a single family only in 1966 and there is still a debate about whether all these languages share a common root.[80] The Lugbara belong to the large Central Sudanic group of languages, while the Gumuz language has been hard to classify within the Nilo-Saharan family; the language may be an early branch from the family or it may be a language isolate and not related to Nilo-Saharan languages at all.[12] Genetic evidence has shown that Gumuz speakers are closely related to other Nilo-Saharan speaking groups from West Ethiopia, Sudan, and Sud-Sudan[5] and are well differentiated from neighboring Afro-Asiatic populations (Figure 2 and Table S5A). Our data show that F_{ST} between the Lugbara and the Gumuz (0.025) exceeds that between African Niger-Congo A and Niger Congo B populations (mean = 0.008, SE 0.0005) and also exceeded that within European, East Asian, and South Asian populations but not the American population in the 1000 Genomes data (Tables S5B and S5C). This is consistent with the relatively large F_{ST} between the Lugbara and the Gumuz being caused by differences in admixture history as well as isolation.

The two Nilo-Saharan populations also appeared very different in the F3 analyses (Figures 4 and S8). The Gumuz

Table 2. Top-Ranked Extreme Signatures that Are Highly Differentiated between the Lugbara and Basoga Populations

Chr	Gene	|iHS| Max	|iHS| Mean	Frequency iHS > 2	No.of SNPs iHS > 2	TajimaD_mean [UNL]	FST_Mean [UNL-UBB]	xpEHH_Max [UNL-UBB]	Rank Score
3	*NEK4*	3.21	3.35	0.24	48/199	2.05	0.06	4.38	61
3	*COLQ*	4.15	3.37	0.23	43/189	1.92	0.02	3.58	62
11	*UVRAG*	4.14	3.31	0.23	72/312	1.73	0.03	3.88	68
7	*FAM3C*	4.87	3.10	0.19	51/265	2.40	0.04	2.94	70
12	*MGAT4C*	3.63	3.65	0.23	66/283	1.95	0.02	3.02	77
5	*ATP10B*	4.31	3.08	0.21	61/291	1.84	0.02	4.60	88
5	*TENM2*	3.44	3.19	0.34	104/305	1.73	0.01	4.23	90
3	*SMIM4*	4.04	3.07	0.27	57/208	0.36	0.05	3.57	91
11	*DGAT2*	4.14	3.26	0.23	72/312	1.45	0.02	2.32	95
5	*C5orf30*	3.50	3.04	0.17	38/218	2.34	0.05	3.42	101
3	*HACL1*	4.15	3.98	0.23	43/189	1.03	0.01	1.69	105
3	*GNL3*	3.21	3.00	0.24	48/199	2.05	0.08	2.67	106
10	*CYP2C8*	4.43	3.04	0.17	68/404	2.50	0.02	1.19	108
2	*ATP5G3*	3.70	3.21	0.17	48/279	1.82	0.01	3.32	111
10	*PDLIM1*	3.68	3.15	0.16	55/337	1.76	0.02	3.03	111
1	*WDR3*	3.80	3.18	0.15	21/136	1.61	0.01	4.17	113
22	*POLR2F*	4.99	3.35	0.23	45/200	0.88	0.00	1.26	115
14	*TEX22*	3.23	3.34	0.15	38/262	2.30	0.02	2.53	117
10	*C10orf129*	3.68	3.03	0.16	55/337	3.46	0.04	1.86	119
3	*DUSP7*	3.57	3.17	0.26	43/165	0.12	0.03	1.79	122

Genes were ranked separately for xpEHH, F_{ST}, and Tajima D. The rank score was obtained by ranking genes separately by Tajima D, F_{ST}, and xpEHH and then an overall score was obtained by summing the ranks of the three metrics.

was most similar to the Afro-Asiatics with respect to their African component, in that there was evidence of shared ancestry to the Luhya (Figure 4A) when paired with any Niger-Congo B or Nigerian population and to the Basoga (Figure 4B) when paired with the Zambian population. The Lugbara, in contrast, appeared as a source population for the Basoga and Luhya only when paired with the Zambian population. This difference is surprising given the similarity of the two Nilo-Saharan populations in the admixture plots at most values of K. The patterns of genetic contribution from the Lugbara and Gumuz to the Luhya and Basoga in the F3 data are most consistent with the Admixture data at K = 6 where Gumuz but not Lugbara share a small ancestry component with the Afro-Asiatics. This component (pink) is also present in the Luhya but is marginal in the Basoga (Figure 3; K = 6). This component shared between the Gumuz, Basoga, and Luhya may represent an ancient East African population that was present before the Bantu Expansion.

The data are consistent with the Gumuz being genetically members of the Nilo-Saharan family and not an isolate, as some linguists have suggested.[10,12] The large genetic distance between the Lugbara and Gumuz may be indicative of the deep splits within the Nilo-Saharan family, which merit much greater efforts to capture. A recent study included 2–4 samples from each of 9 lineages, supports the large genetic diversity within this family, and indicates that this family is a rich source of novel genetic variation.[6] With sequence information from further Nilo-Saharan populations, the genetic relationship of the Lugbara and Gumuz to other members of the family will also be resolved.

Signatures of Selection

We identified signatures of selection in multiple genes associated with immune responses and other conditions. However, the multiple and diverse functions of individual genes make it hard to predict the specific adaptations or phenotypes that might have driven selection at these loci. Nevertheless, there was a group of genes associated with skin tone and hair form which are plausibly associated with the particularly dark color of the skin of Nilo-Saharans and the intense UV radiation they experience. *UVRAG* showed the third greatest combined evidence for selection in Lugbara but not Basoga (Table 2). This gene, which is involved in melanine deposition in response to ultraviolet (UV) radiation,[73] has not previously been found under selection. Two other genes involved in skin

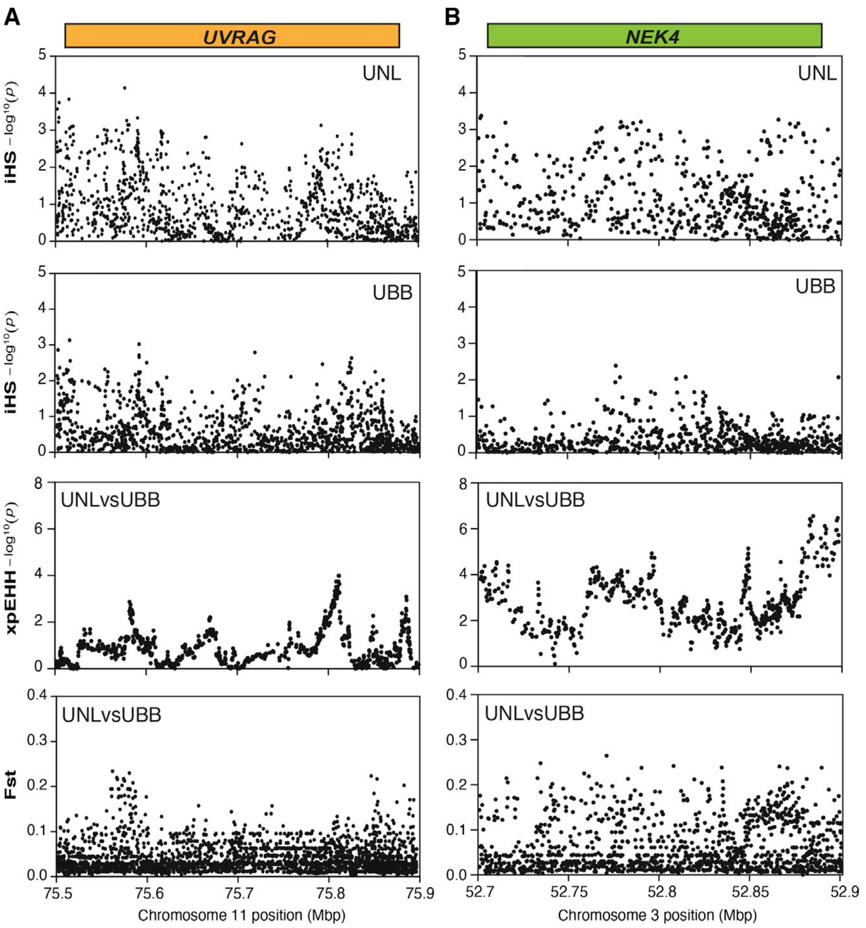

Figure 6. Signatures of Selection Unique to the Uganda Nilotic Lugbara Population
Evidence (iHS, xpEHH, and Tajima D) for differential selection signatures between Lugbara (UNL) and Basoga (UBB) at the *UVRAG* locus on chromosome 11 (A) and the *NEK4* locus on chromosome 1 (B).

pigmentation (*SNX13* and *TYROBP*) were in the top 1% of gene regions under selection in Lugbara and were also under selection in Basoga (Table S8) and a further five genes involved in skin pigmentation (*IRF4, TYRP1, HERC2, SLC24A5, OPRM1*) had some evidence of selection (Table S7).[81] Therefore, 7 of the 18 genes previously associated with skin pigmentation by Martin et al.[81] had some evidence of selection in this study.

Nilo-Saharans have some of the darkest skin tones in the world[82] and the Lugbara generally have a darker skin compared to the Basoga.[83] Skin reflectance is correlated with UV radiation[84] and the dark skin tones of the Nilo-Saharans could be an adaptation to the open savannah conditions of the Sahel where there is limited tree and cloud cover and which is predicted by models to be one of the regions of the world with darkest skin pigmentation.[84] *UVRAG* may be an important contributor to the exceptionally dark skin tones of the Nilo-Saharans in conjunction with *SNX13* and *TYROBP* in particular and possibly also *IRF4, TYRP1, HERC2, SLC24A5,* and *OPRM1.*

Hair form is probably related to thermoregulation by helping keep the head cool during exercise.[85] 6 keratin and 16 keratin-associated proteins, which are involved in hair formation, were in 3 regions with evidence of selection on chromosomes 12, 17, and 21 (Table S7) and selection for hair form as well as skin color could be part of a suite of traits for adaptation to the harsh conditions of the Sahel where the majority of Nilo-Saharan populations are found.

In conclusion, the Nilo-Saharan language speakers are an under-represented source for discovery of genetic variation. They are more genetically differentiated than the neighboring Afro-Asiatic and Niger-Congo groups but have been much less studied. They have contributed a large component to the genome of Afro-Asiatic speakers[26] and a smaller proportion of the genomes of East African Niger-Congo-B speakers. There is evidence for selection for skin color and hair form, which could be adaptive for the semi-arid Sahel where the majority of Nilo-Saharan populations live. Linguistic evidence suggests that substantial further genetic diversity remains to be discovered within the Nilo-Saharan group, which should be a priority for further genome analysis studies.

Data and Code Availability

The datasets generated and/or analyzed during the current study are available from the corresponding author on reasonable request. The sequenced data have been submitted to the EGA by H3ABionet under the study accession number EGA: EGAS00001002602.

Supplemental Data

Supplemental Data can be found online at https://doi.org/10.1016/j.ajhg.2020.07.007.

Acknowledgments

The authors would like to acknowledge the study participants who donated their specimens, the personnel involved in the community engagement and coordinating sample collection and processing, and the national sleeping sickness control programs of the participating countries. We thank Dr. Neil Hall and Dr. Andy Tait for their expert advice on the study strategy. We thank Dr. Zane Lombard (University of Witwatersrand) and Dr. Adebowale Adeyemo (NHGRI) for facilitating sequencing of samples from Zambia and Cameroon at Baylor College of Medicine as well as the H3ABionet for training and support on data analysis. Fiona Marshall and Rebecca Grollemund

are acknowledged for their helpful discussions on African history. This study was funded by the African Academy of Sciences/Wellcome project ID H3A/18/004 as part of the H3Africa consortia.

Declaration of Interests

The authors declare no competing interests.

Received: July 3, 2020
Accepted: July 13, 2020
Published: August 10, 2020

Web Resources

European Genome-phenome Archive (EGA), https://www.ebi.ac.uk/ega

QGIS, https://qgis.org/en/site/

References

1. Campbell, M.C., Hirbo, J.B., Townsend, J.P., and Tishkoff, S.A. (2014). The peopling of the African continent and the diaspora into the new world. Curr. Opin. Genet. Dev. *29*, 120–132.
2. Gurdasani, D., Carstensen, T., Tekola-Ayele, F., Pagani, L., Tachmazidou, I., Hatzikotoulas, K., Karthikeyan, S., Iles, L., Pollard, M.O., Choudhury, A., et al. (2015). The African Genome Variation Project shapes medical genetics in Africa. Nature *517*, 327–332.
3. Molinaro, L., Montinaro, F., Yelmen, B., Marnetto, D., Behar, D.M., Kivisild, T., and Pagani, L. (2019). West Asian sources of the Eurasian component in Ethiopians: a reassessment. Sci. Rep. *9*, 18811.
4. Tishkoff, S.A., Reed, F.A., Friedlaender, F.R., Ehret, C., Ranciaro, A., Froment, A., Hirbo, J.B., Awomoyi, A.A., Bodo, J.-M., Doumbo, O., et al. (2009). The genetic structure and history of Africans and African Americans. Science *324*, 1035–1044.
5. Pagani, L., Kivisild, T., Tarekegn, A., Ekong, R., Plaster, C., Gallego Romero, I., Ayub, Q., Mehdi, S.Q., Thomas, M.G., Luiselli, D., et al. (2012). Ethiopian genetic diversity reveals linguistic stratification and complex influences on the Ethiopian gene pool. Am. J. Hum. Genet. *91*, 83–96.
6. Fan, S., Kelly, D.E., Beltrame, M.H., Hansen, M.E.B., Mallick, S., Ranciaro, A., Hirbo, J., Thompson, S., Beggs, W., Nyambo, T., et al. (2019). African evolutionary history inferred from whole genome sequence data of 44 indigenous African populations. Genome Biol. *20*, 82.
7. Li, S., Schlebusch, C., and Jakobsson, M. (2014). Genetic variation reveals large-scale population expansion and migration during the expansion of Bantu-speaking peoples. Proc. Biol. Sci. *281*, 20141448.
8. Lewis, M.P., Simons, F.G., and Fenning, D.C. (2016). Ethnologue: Languages of the World, Nineteenth Edition (SIL International).
9. Hollfelder, N., Schlebusch, C.M., Günther, T., Babiker, H., Hassan, H.Y., and Jakobsson, M. (2017). Northeast African genomic variation shaped by the continuity of indigenous groups and Eurasian migrations. PLoS Genet. *13*, e1006976.
10. Bender, M.L. (2000). Nilo-Saharan. In African Languages, B. Heine and D. Nurse, eds. (Cambridge University Press).
11. T. Güldemann, ed. (2018). The Languages and Linguistics of Africa (Berlin, Boston: De Gruyter).
12. Dimmendaal, G.J. (2008). Language Ecology and Linguistic Diversity on the African Continent. Lang. Linguist. Compass *2*, 840–858.
13. Mallick, S., Li, H., Lipson, M., Mathieson, I., Gymrek, M., Racimo, F., Zhao, M., Chennagiri, N., Nordenfelt, S., Tandon, A., et al. (2016). The Simons Genome Diversity Project: 300 genomes from 142 diverse populations. Nature *538*, 201–206.
14. Hsieh, P., Veeramah, K.R., Lachance, J., Tishkoff, S.A., Wall, J.D., Hammer, M.F., and Gutenkunst, R.N. (2016). Whole-genome sequence analyses of Western Central African Pygmy hunter-gatherers reveal a complex demographic history and identify candidate genes under positive natural selection. Genome Res. *26*, 279–290.
15. Lachance, J., Vernot, B., Elbers, C.C., Ferwerda, B., Froment, A., Bodo, J.-M., Lema, G., Fu, W., Nyambo, T.B., Rebbeck, T.R., et al. (2012). Evolutionary history and adaptation from high-coverage whole-genome sequences of diverse African hunter-gatherers. Cell *150*, 457–469.
16. Auton, A., Brooks, L.D., Durbin, R.M., Garrison, E.P., Kang, H.M., Korbel, J.O., Marchini, J.L., McCarthy, S., McVean, G.A., Abecasis, G.R.; and 1000 Genomes Project Consortium (2015). A global reference for human genetic variation. Nature *526*, 68–74.
17. Abecasis, G.R., Altshuler, D., Auton, A., Brooks, L.D., Durbin, R.M., Gibbs, R.A., Hurles, M.E., McVean, G.A.; and 1000 Genomes Project Consortium (2010). A map of human genome variation from population-scale sequencing. Nature *467*, 1061–1073.
18. Ma, Y., Ding, X., Qanbari, S., Weigend, S., Zhang, Q., and Simianer, H. (2015). Properties of different selection signature statistics and a new strategy for combining them. Heredity *115*, 426–436.
19. Shriner, D., and Keita, S.O.Y. (2016). Migration Route Out of Africa Unresolved by 225 Egyptian and Ethiopian Whole Genome Sequences. Front. Genet. *7*, 98.
20. Cooper, A., Ilboudo, H., Alibu, V.P., Ravel, S., Enyaru, J., Weir, W., Noyes, H., Capewell, P., Camara, M., Milet, J., et al. (2017). *APOL1* renal risk variants have contrasting resistance and susceptibility associations with African trypanosomiasis. eLife *6*, 56.
21. Shriner, D., and Rotimi, C.N. (2018). Whole-Genome-Sequence-Based Haplotypes Reveal Single Origin of the Sickle Allele during the Holocene Wet Phase. Am. J. Hum. Genet. *102*, 547–556.
22. Ilboudo, H., Noyes, H., Mulindwa, J., Kimuda, M.P., Koffi, M., Kaboré, J.W., Ahouty, B., Ngoyi, D.M., Fataki, O., Simo, G., et al.; TrypanoGEN Research Group as members of The H3Africa Consortium (2017). Introducing the TrypanoGEN biobank: A valuable resource for the elimination of human African trypanosomiasis. PLoS Negl. Trop. Dis. *11*, e0005438.
23. Li, H., and Durbin, R. (2009). Fast and accurate short read alignment with Burrows-Wheeler transform. Bioinformatics *25*, 1754–1760.
24. McKenna, A., Hanna, M., Banks, E., Sivachenko, A., Cibulskis, K., Kernytsky, A., Garimella, K., Altshuler, D., Gabriel, S., Daly, M., and DePristo, M.A. (2010). The Genome Analysis Toolkit: a MapReduce framework for analyzing next-generation DNA sequencing data. Genome Res. *20*, 1297–1303.
25. Nyangiri, O.A., Noyes, H., Mulindwa, J., Ilboudo, H., Kabore, J.W., Ahouty, B., Koffi, M., Asina, O.F., Mumba, D., Ofon, E., et al.; TrypanoGEN Research Group, as members of The H3Africa Consortium (2020). Copy number variation in human genomes from three major ethno-linguistic groups in Africa. BMC Genomics *21*, 289.

26. Pagani, L., Schiffels, S., Gurdasani, D., Danecek, P., Scally, A., Chen, Y., Xue, Y., Haber, M., Ekong, R., Oljira, T., et al. (2015). Tracing the route of modern humans out of Africa by using 225 human genome sequences from Ethiopians and Egyptians. Am. J. Hum. Genet. *96*, 986–991.
27. Li, H. (2011). A statistical framework for SNP calling, mutation discovery, association mapping and population genetical parameter estimation from sequencing data. Bioinformatics *27*, 2987–2993.
28. Delaneau, O., Zagury, J.-F., and Marchini, J. (2013). Improved whole-chromosome phasing for disease and population genetic studies. Nat. Methods *10*, 5–6.
29. Danecek, P., Auton, A., Abecasis, G., Albers, C.A., Banks, E., DePristo, M.A., Handsaker, R.E., Lunter, G., Marth, G.T., Sherry, S.T., et al.; 1000 Genomes Project Analysis Group (2011). The variant call format and VCFtools. Bioinformatics *27*, 2156–2158.
30. Purcell, S., Neale, B., Todd-Brown, K., Thomas, L., Ferreira, M.A.R., Bender, D., Maller, J., Sklar, P., de Bakker, P.I.W., Daly, M.J., and Sham, P.C. (2007). PLINK: a tool set for whole-genome association and population-based linkage analyses. Am. J. Hum. Genet. *81*, 559–575.
31. R Core Team (2008). R: A language and environment for statistical computing (Vienna, Austria).
32. Alexander, D.H., Novembre, J., and Lange, K. (2009). Fast model-based estimation of ancestry in unrelated individuals. Genome Res. *19*, 1655–1664.
33. Patterson, N., Moorjani, P., Luo, Y., Mallick, S., Rohland, N., Zhan, Y., Genschoreck, T., Webster, T., and Reich, D. (2012). Ancient admixture in human history. Genetics *192*, 1065–1093.
34. Petr, M., Vernot, B., and Kelso, J. (2019). admixr-R package for reproducible analyses using ADMIXTOOLS. Bioinformatics *35*, 3194–3195.
35. Cadzow, M., Boocock, J., Nguyen, H.T., Wilcox, P., Merriman, T.R., and Black, M.A. (2014). A bioinformatics workflow for detecting signatures of selection in genomic data. Front. Genet. *5*, 293.
36. Wright, S. (1922). Coefficient of inbreeding and relationship. Am. Naturalist *56*, 330–338.
37. Tajima, F. (1989). Statistical method for testing the neutral mutation hypothesis by DNA polymorphism. Genetics *123*, 585–595.
38. Wright, S. (1950). Genetical structure of populations. Nature *166*, 247–249.
39. Sabeti, P.C., Reich, D.E., Higgins, J.M., Levine, H.Z.P., Richter, D.J., Schaffner, S.F., Gabriel, S.B., Platko, J.V., Patterson, N.J., McDonald, G.J., et al. (2002). Detecting recent positive selection in the human genome from haplotype structure. Nature *419*, 832–837.
40. Gautier, M., and Vitalis, R. (2012). rehh: an R package to detect footprints of selection in genome-wide SNP data from haplotype structure. Bioinformatics *28*, 1176–1177.
41. Voight, B.F., Kudaravalli, S., Wen, X., and Pritchard, J.K. (2006). A map of recent positive selection in the human genome. PLoS Biol. *4*, e72.
42. Tang, K., Thornton, K.R., and Stoneking, M. (2007). A new approach for using genome scans to detect recent positive selection in the human genome. PLoS Biol. *5*, e171.
43. Quinlan, A.R., and Hall, I.M. (2010). BEDTools: a flexible suite of utilities for comparing genomic features. Bioinformatics *26*, 841–842.
44. Korneliussen, T.S., Moltke, I., Albrechtsen, A., and Nielsen, R. (2013). Calculation of Tajima's D and other neutrality test statistics from low depth next-generation sequencing data. BMC Bioinformatics *14*, 289.
45. van Dorp, L., Balding, D., Myers, S., Pagani, L., Tyler-Smith, C., Bekele, E., Tarekegn, A., Thomas, M.G., Bradman, N., and Hellenthal, G. (2015). Evidence for a Common Origin of Blacksmiths and Cultivators in the Ethiopian Ari within the Last 4500 Years: Lessons for Clustering-Based Inference. PLoS Genet. *11*, e1005397.
46. Hartmann, S., Ridley, A.J., and Lutz, S. (2015). The Function of Rho-Associated Kinases ROCK1 and ROCK2 in the Pathogenesis of Cardiovascular Disease. Front. Pharmacol. *6*, 276.
47. Kim, A.Y., Bommeljé, C.C., Lee, B.E., Yonekawa, Y., Choi, L., Morris, L.G., Huang, G., Kaufman, A., Ryan, R.J.H., Hao, B., et al. (2008). SCCRO (DCUN1D1) is an essential component of the E3 complex for neddylation. J. Biol. Chem. *283*, 33211–33220.
48. Meyer, D., and Thomson, G. (2001). How selection shapes variation of the human major histocompatibility complex: a review. Ann. Hum. Genet. *65*, 1–26.
49. Möröy, T., and Khandanpour, C. (2019). Role of GFI1 in Epigenetic Regulation of MDS and AML Pathogenesis: Mechanisms and Therapeutic Implications. Front. Oncol. *9*, 824.
50. Weidinger, S., Illig, T., Baurecht, H., Irvine, A.D., Rodriguez, E., Diaz-Lacava, A., Klopp, N., Wagenpfeil, S., Zhao, Y., Liao, H., et al. (2006). Loss-of-function variations within the filaggrin gene predispose for atopic dermatitis with allergic sensitizations. J. Allergy Clin. Immunol. *118*, 214–219.
51. Rung, J., Cauchi, S., Albrechtsen, A., Shen, L., Rocheleau, G., Cavalcanti-Proença, C., Bacot, F., Balkau, B., Belisle, A., Borch-Johnsen, K., et al. (2009). Genetic variant near IRS1 is associated with type 2 diabetes, insulin resistance and hyperinsulinemia. Nat. Genet. *41*, 1110–1115.
52. Miller, J.L. (2013). Iron deficiency anemia: a common and curable disease. Cold Spring Harb. Perspect. Med. *3*, a011866–a011866.
53. Lencz, T., Guha, S., Liu, C., Rosenfeld, J., Mukherjee, S., DeRosse, P., John, M., Cheng, L., Zhang, C., Badner, J.A., et al. (2013). Genome-wide association study implicates NDST3 in schizophrenia and bipolar disorder. Nat. Commun. *4*, 2739.
54. Huang, L., Ye, K., McGee, M.C., Nidetz, N.F., Elmore, J.P., Limper, C.B., Southard, T.L., Russell, D.G., August, A., and Huang, W. (2020). Interleukin-2-Inducible T-Cell Kinase Deficiency Impairs Early Pulmonary Protection Against *Mycobacterium tuberculosis* Infection. Front. Immunol. *10*, 3103.
55. Sims, B., Farrow, A.L., Williams, S.D., Bansal, A., Krendelchtchikov, A., Gu, L., and Matthews, Q.L. (2017). Role of TIM-4 in exosome-dependent entry of HIV-1 into human immune cells. Int. J. Nanomedicine *12*, 4823–4833.
56. Vyakarnam, A., Sidebottom, D., Murad, S., Underhill, J.A., Easterbrook, P.J., Dalgleish, A.G., and Peakman, M. (2004). Possession of human leucocyte antigen DQ6 alleles and the rate of CD4 T-cell decline in human immunodeficiency virus-1 infection. Immunology *112*, 136–142.
57. Delgado, J.C., Baena, A., Thim, S., and Goldfeld, A.E. (2006). Aspartic acid homozygosity at codon 57 of HLA-DQ beta is associated with susceptibility to pulmonary tuberculosis in Cambodia. J. Immunol. *176*, 1090–1097.
58. Singh, G.C., Ahmed, M., Zaid, M., and Hasnain, S. (2020). Biochemical, serological, and genetic aspects related to gene

HLA-DQB1 and its association with type 1 diabetes mellitus (T1DM). Mol. Genet. Genomic Med. *8*, e1147.
59. Malnic, B., Godfrey, P.A., and Buck, L.B. (2004). The human olfactory receptor gene family. Proc. Natl. Acad. Sci. USA *101*, 2584–2589.
60. Li, Y., Pohl, E., Boulouiz, R., Schraders, M., Nürnberg, G., Charif, M., Admiraal, R.J.C., von Ameln, S., Baessmann, I., Kandil, M., et al. (2010). Mutations in TPRN cause a progressive form of autosomal-recessive nonsyndromic hearing loss. Am. J. Hum. Genet. *86*, 479–484.
61. Joshi, H., McIntyre, W.B., Kooner, S., Rathbone, M., Gabriele, S., Gabriele, J., Baranowski, D., Frey, B.N., and Mishra, R.K. (2020). Decreased Expression of Cerebral Dopamine Neurotrophic Factor in Platelets of Stroke Patients. J. Stroke Cerebrovasc. Dis. *29*, 104502.
62. Yang, Y., Quach, C., and Liang, C. (2016). Autophagy modulator plays a part in UV protection. Autophagy *12*, 1677–1678.
63. Berois, N., Gattolliat, C.-H., Barrios, E., Capandeguy, L., Douc-Rasy, S., Valteau-Couanet, D., Bénard, J., and Osinaga, E. (2013). GALNT9 gene expression is a prognostic marker in neuroblastoma patients. Clin. Chem. *59*, 225–233.
64. Seidahmed, M.Z., Al-Kindi, A., Alsaif, H.S., Miqdad, A., Alabbad, N., Alfifi, A., Abdelbasit, O.B., Alhussein, K., Alsamadi, A., Ibrahim, N., et al. (2020). Recessive mutations in SCYL2 cause a novel syndromic form of arthrogryposis in humans. Hum. Genet. *139*, 513–519.
65. Youssefian, L., Vahidnezhad, H., Saeidian, A.H., Sotoudeh, S., Mahmoudi, H., Daneshpazhooh, M., Aghazadeh, N., Adams, R., Ghanadan, A., Zeinali, S., et al. (2017). Autosomal recessive congenital ichthyosis: CERS3 mutations identified by a next generation sequencing panel targeting ichthyosis genes. Eur. J. Hum. Genet. *25*, 1282–1285.
66. Manjarrez-Orduño, N., Marasco, E., Chung, S.A., Katz, M.S., Kiridly, J.F., Simpfendorfer, K.R., Freudenberg, J., Ballard, D.H., Nashi, E., Hopkins, T.J., et al. (2012). CSK regulatory polymorphism is associated with systemic lupus erythematosus and influences B-cell signaling and activation. Nat. Genet. *44*, 1227–1230.
67. Lv, L., Wang, J., Gao, B., Wu, L., Wang, F., Cui, Z., He, K., Zhang, L., Chen, M., and Zhao, M.-H. (2018). Serum uromodulin and progression of kidney disease in patients with chronic kidney disease. J. Transl. Med. *16*, 316.
68. Smith, E.E., and Malik, H.S. (2009). The apolipoprotein L family of programmed cell death and immunity genes rapidly evolved in primates at discrete sites of host-pathogen interactions. Genome Res. *19*, 850–858.
69. Sabeti, P.C., Varilly, P., Fry, B., Lohmueller, J., Hostetter, E., Cotsapas, C., Xie, X., Byrne, E.H., McCarroll, S.A., Gaudet, R., et al.; International HapMap Consortium (2007). Genome-wide detection and characterization of positive selection in human populations. Nature *449*, 913–918.
70. Takata, A., Matsumoto, N., and Kato, T. (2017). Genome-wide identification of splicing QTLs in the human brain and their enrichment among schizophrenia-associated loci. Nat. Commun. *8*, 14519.
71. Wu, C., Jin, X., Tsueng, G., Afrasiabi, C., and Su, A.I. (2016). BioGPS: building your own mash-up of gene annotations and expression profiles. Nucleic Acids Res. *44* (D1), D313–D316.
72. Campbell, A.R., Regan, K., Bhave, N., Pattanayak, A., Parihar, R., Stiff, A.R., Trikha, P., Scoville, S.D., Liyanarachchi, S., Kondadasula, S.V., et al. (2015). Gene expression profiling of the human natural killer cell response to Fc receptor activation: unique enhancement in the presence of interleukin-12. BMC Med. Genomics *8*, 66.
73. Li, S., Jang, G.-B., Quach, C., and Liang, C. (2019). Darkening with UVRAG. Autophagy *15*, 366–367.
74. Sosinsky, A., Ambrose, J., Zarowiecki, M., Mitchell, J., Henderson, S., Murugaesu, N., Hamblin, A., Turnbull, C., Walker, S., Perez-Gil, D., et al. (2019). 100,000 genomes project: Integrating whole genome sequencing (WGS) data into clinical practice. Ann. Oncol. *30*, vii1.
75. Gao, Y., Zhang, C., Yuan, L., Ling, Y., Wang, X., Liu, C., Pan, Y., Zhang, X., Ma, X., Wang, Y., et al.; Han100K Initiative (2020). PGG.Han: the Han Chinese genome database and analysis platform. Nucleic Acids Res. *48* (D1), D971–D976.
76. Rutter, J.L., Goldstein, D.B., Denny, J.C., Philip-pakis, A., Smoller, J.W., and Jenkins, G. (2019). The "All of Us" Research Program. N. Engl. J. Med. *381*, 1–9.
77. Teo, Y.-Y., Small, K.S., and Kwiatkowski, D.P. (2010). Methodological challenges of genome-wide association analysis in Africa. Nat. Rev. Genet. *11*, 149–160.
78. Jallow, M., Teo, Y.-Y., Small, K.S., Rockett, K.A., Deloukas, P., Clark, T.G., Kivinen, K., Bojang, K.A., Conway, D.J., Pinder, M., et al.; Wellcome Trust Case Control Consortium; and Malaria Genomic Epidemiology Network (2009). Genome-wide and fine-resolution association analysis of malaria in West Africa. Nat. Genet. *41*, 657–665.
79. Rotimi, C., Abayomi, A., Abimiku, A., Adabayeri, V.M., Adebamowo, C., Adebiyi, E., Ademola, A.D., Adeyemo, A., Adu, D., Affolabi, D., et al.; H3Africa Consortium (2014). Research capacity. Enabling the genomic revolution in Africa. Science *344*, 1346–1348.
80. Greenberg, J.H. (1966). The languages of Africa (Cambridge University Press).
81. Martin, A.R., Lin, M., Granka, J.M., Myrick, J.W., Liu, X., Sockell, A., Atkinson, E.G., Werely, C.J., Möller, M., Sandhu, M.S., et al. (2017). An Unexpectedly Complex Architecture for Skin Pigmentation in Africans. Cell *171*, 1340–1353.e14.
82. Barsh, G.S. (2003). What controls variation in human skin color? PLoS Biol. *1*, E27.
83. Roberts, D.F., and Bainbridge, D.R. (1963). Nilotic Physique. Am. J. Phys. Anthropol. *21*, 341–370.
84. Chaplin, G. (2004). Geographic distribution of environmental factors influencing human skin coloration. Am. J. Phys. Anthropol. *125*, 292–302.
85. Jablonski, N.G., and Chaplin, G. (2014). The evolution of skin pigmentation and hair texture in people of African ancestry. Dermatol. Clin. *32*, 113–121.

The American Journal of Human Genetics, Volume *107*

Supplemental Data

High Levels of Genetic Diversity within Nilo-Saharan Populations: Implications for Human Adaptation

Julius Mulindwa, Harry Noyes, Hamidou Ilboudo, Luca Pagani, Oscar Nyangiri, Magambo Phillip Kimuda, Bernardin Ahouty, Olivier Fataki Asina, Elvis Ofon, Kelita Kamoto, Justin Windingoudi Kabore, Mathurin Koffi, Dieudonne Mumba Ngoyi, Gustave Simo, John Chisi, Issa Sidibe, John Enyaru, Martin Simuunza, Pius Alibu, Vincent Jamonneau, Mamadou Camara, Andy Tait, Neil Hall, Bruno Bucheton, Annette MacLeod, Christiane Hertz-Fowler, Enock Matovu, and the TrypanoGEN Research Group of the H3Africa Consortium

Supplemental Data

Sequence Quality

Samples from five populations (CIV, GAS, UNL, UBB, DRC) were sequenced to 10X coverage and the remaining two populations (CAM and ZAM) were sequenced to 30X coverage.

There are two strategies within GATK for calling SNP from sequence data. 1) Combine the data from all samples and call SNP jointly and output just variant loci into a vcf file; 2) Call SNP on individual samples, output all loci into a gvcf file and combine the gvcf files later. The first strategy has the advantage of having more data to work with to assess quality metrics and cut-offs for SNP calling, however it is difficult to combine data that has been sequenced to different depths as different criteria need to be applied to each sample depending on depth of coverage. The second strategy is not affected by differences in sequence coverage and has the added advantage of making it easy to add data from additional samples as they become available without having to repeat the complete joint SNP calling on all samples. The second strategy was used in this project.

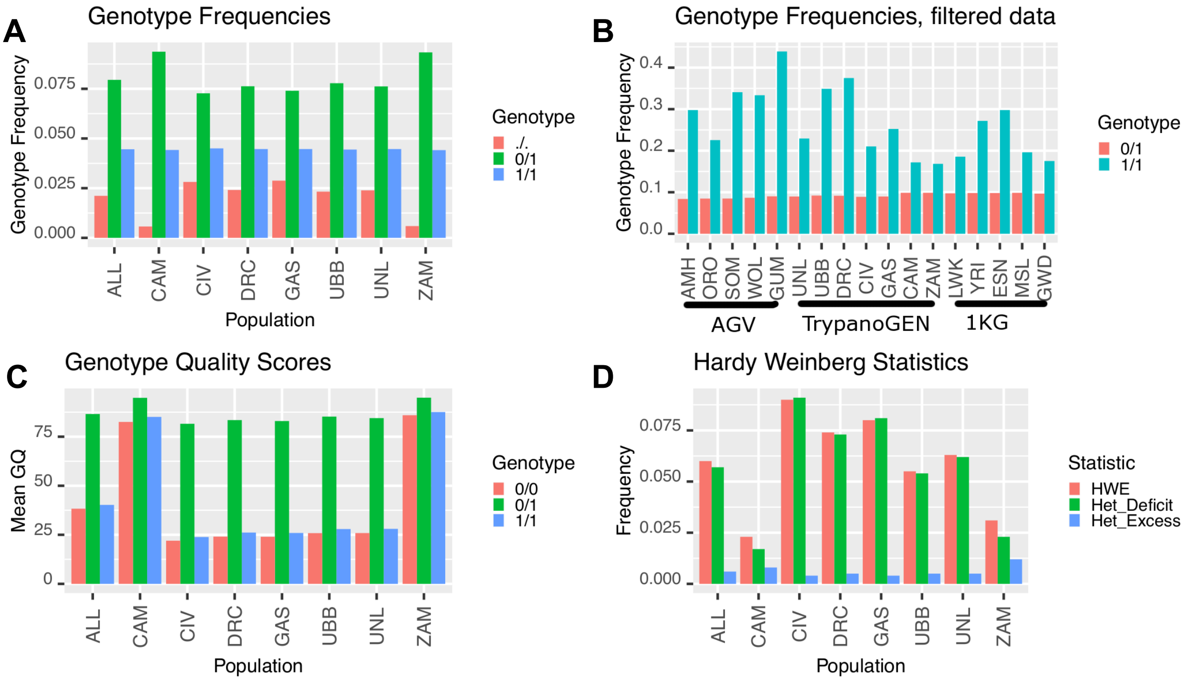

Figure S1. **Sequence Quality Metrics by Sample Population**. (**A**) **Genotype frequencies before filtering**, null genotypes are shown as (./.), heterozygotes (0/1) and homozygote alternate allele genotype (1/1). Homozygous reference genotypes (0/0) were the largest class (>80%) and are not shown for clarity. The small numbers of genotypes at multiallelic loci are also not shown. Note the lower frequency of null genotypes and higher frequency of heterozygotes in the Cameroon and Zambian (CAM and ZAM) populations, which were sequenced at 30X coverage whilst the other populations were sequenced at 10X. (**B**) **Genotype frequencies after filtering and merging**, heterozygotes (0/1) and homozygote minor (not alternate) genotypes (1/1), homozygous major genotypes (0/0) are not shown. Note that the frequency of heterozygotes is now very consistent across all populations irrespective of sequence depth, however the frequency of homozygous minor alleles is very variable across all data sources **(C) Mean Genotype Quality scores before filtering and phasing**. In the Cameroon and Zambian populations the Genotype quality scores were similar irrespective of genotype whilst in the populations sequenced at lower coverage the homozygote quality scores were substantially lower than the heterozygote scores. (**D**) **Hardy Weinberg Statistics before filtering and phasing.** The frequencies of loci with $p < 0.05$ are shown for three statistics: HWE, the Hardy Weinberg P value; Het_Deficit, H_0 the number of heterozygotes is not less than expected; Het_Excess, H_0 the number of heterozygotes is not greater than expected. The Cameroon

and Zambian populations had about a third of the number of loci that were not in hardy Weinberg Equilibrium as the other populations. In these two populations a higher proportion of loci that were not in Hardy Weinberg Equilibrium had an excess of heterozygotes and a lower proportion had a deficit of heterozygotes.

Figure S1 shows some descriptive statistics for sequence quality for each population and shows that there are clear differences between the samples sequenced at 30X (CAM and ZAM) and those sequenced at 10X before filtering and phasing however after filtering there were no differences that correlated with data source. Fig S1A shows that the frequency of null calls was much higher in the 10X-sequenced samples with a call rate of 97.4% in the 10X samples and 99.4% in the 30X samples. The 30X-sequenced samples also had higher proportions of heterozygotes (9.3%) compared with the 10X sequenced samples (7.5%). Therefore about 1.8% of homozygous calls are likely to be false and should have been called as heterozygotes. This is a known problem with low coverage data and is reflected in the Gentotype Quality (GQ) Scores for the different genotypes (Fig S1C). All samples had high GQ scores for heterozygote loci (Mean 10X = 84; Mean 30X=95), but the homozygotes had much lower scores in the 10X data (Mean 10X = 25; Mean 30X=85) reflecting the lower confidence that a heterozygote has not been missed with 10X data. After filtering and phasing (including imputation of missing data) (Fig S1B) all populations had very similar heterozygote frequencies irrespective of data source. Although the homozygous minor genotype frequency was very variable, it did not correlate with batch suggesting that this was genuine population variation rather than batch effect.
The higher frequency of missing heterozygotes in the 10X data before filtering and phasing is also reflected in the Hardy Weinberg statistics (Fig S1D); 2.7% of loci had $p<0.05$ for Hardy Weinberg equilibrium in the 30X data and 7.3% in the 10X data. Almost all of these loci in all datasets had a deficiency of heterozygotes (Fig S1C). Whilst it is expected that some loci will not be in Hardy-Weinberg equilibrium due to random sampling and also due to selection at some loci for particular alleles, the much higher frequency of loci with low Hardy-Weinberg P values in the 10X data reflects the rate of missing heterozygotes in the unfiltered data.

Despite the evidence that the 10X data quality was generally worse than the 30X quality there was very little evidence of this having a impact on the conclusions. The unfiltered data was only used for describing novel variants and their potential impacts. The filtering and phasing strategy generated a dataset with very similar heterozygote frequencies. The population analyses showed that geographically close populations from the same major linguistic group clustered tightly together irrespective of data source, demonstrating the success of the filtering strategy. In the multidimensional scaling analyses our West African samples (GAS, CIV) with 10X coverage clustered tightly with 1000 Genomes samples from West Africa (MSL, GWD) as expected and our UBB population from Uganda clustered tightly with the 1000 Genomes LWK samples from neighbouring Kenya. Furthermore in the Admixture analysis the number and size of ancestral components were very similar from adjacent 1000 Genomes and our data.

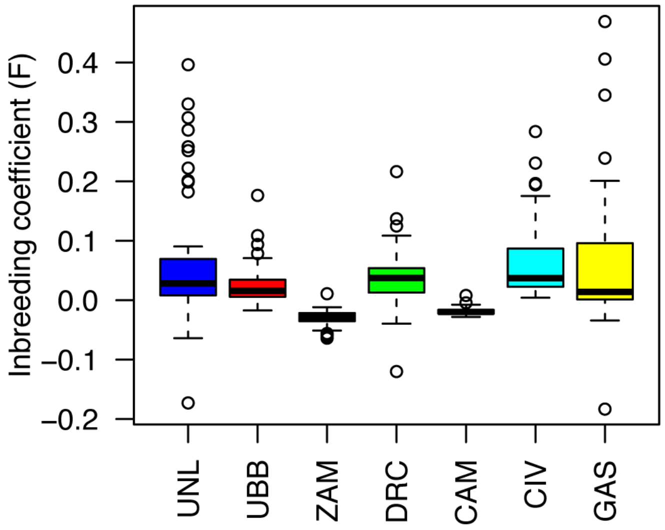

Figure S2. Heterozygosity analysis of the inbreeding coefficient within populations.

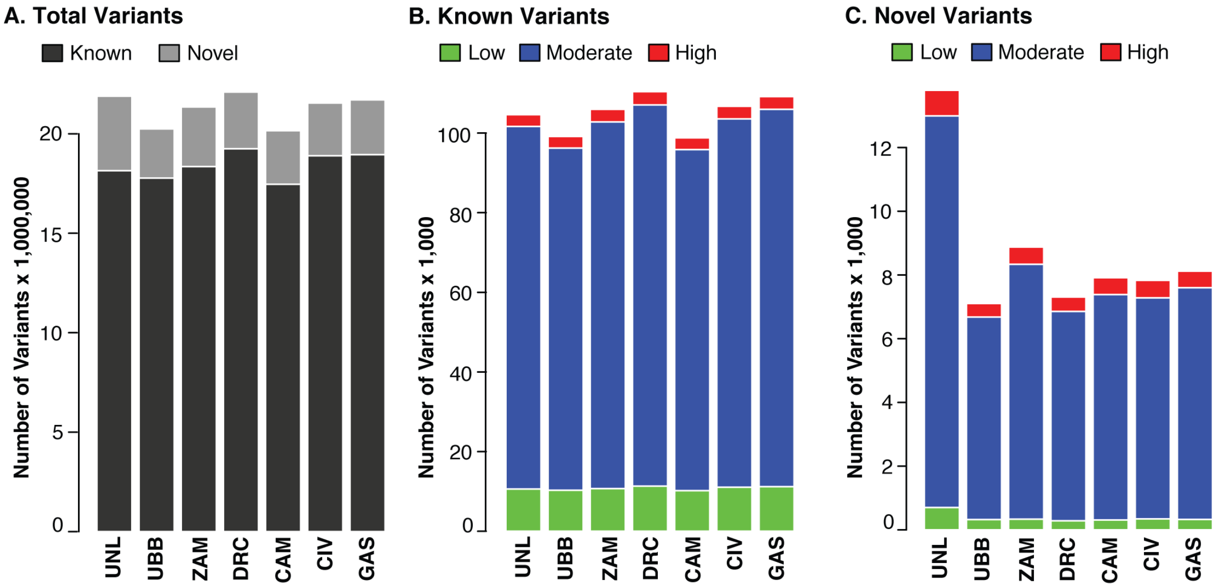

Figure S3. Classification of the genetic variation in the sequenced populations. A. The total number of both the Known (with dbSNP rsID) and Unknown/Novel (without dbSNP rsID) variants; The degree of impact on genome function, predicted by SnpEff, is shown for the Known (**B**) and Unknown/Novel (**C**) variants (see Table S6 for definitions of impacts). SNPs that were classified as "modifier" were mainly in intergenic regions and are excluded from the plot. Variants with multiple impact annotations were assigned to the highest impact annotation. (See Table S4 for the underlying data)

A

B

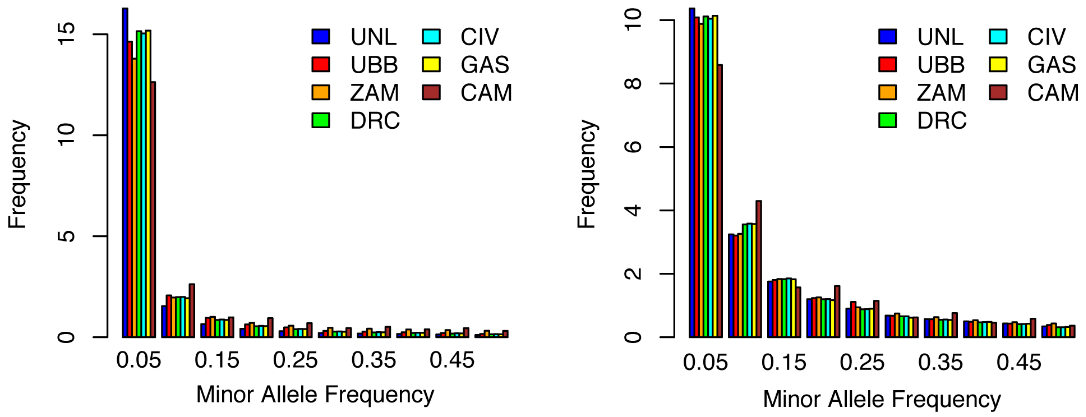

Figure S4. **A**. Minor allele frequency (MAF) distribution of Novel variants, **B**. MAF distribution of known variants.

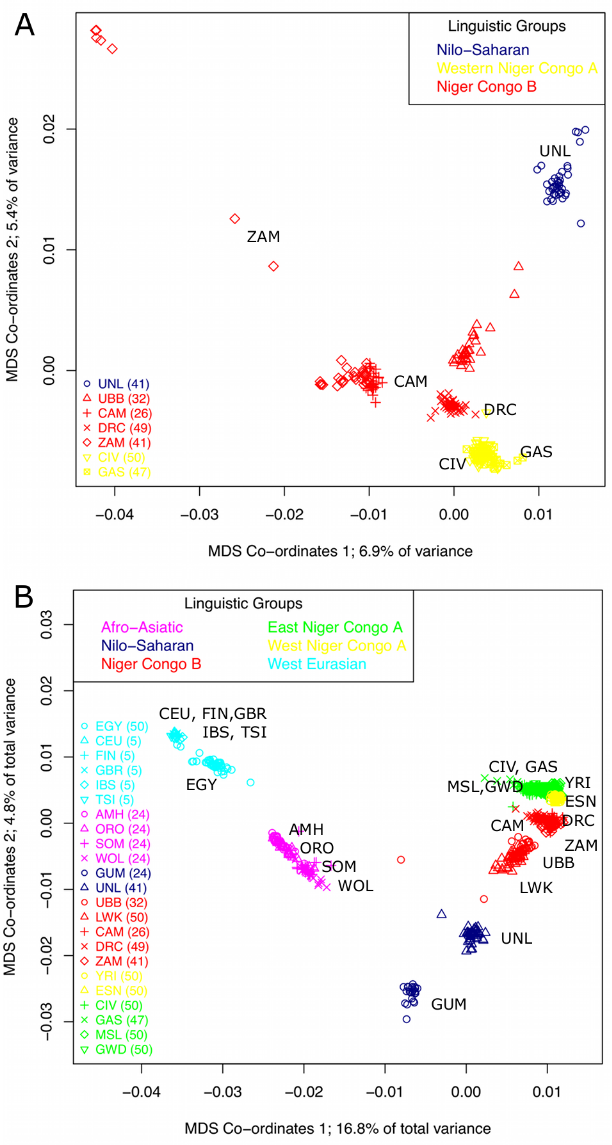

Figure S5. Multidimensional scaling analysis on (**A**) TrypanoGEN populations (**B**) African and European populations**.** Both plots include the 7 Zambian outlier samples that were excluded from Fig2A (but not Fig2B). In (A) the 7 outliers are widely dispersed but in (B) in the much larger context they cluster tightly with the remaining Zambian samples. The numbers in brackets beside each population indicate the number of individuals whose genomes were analysed.

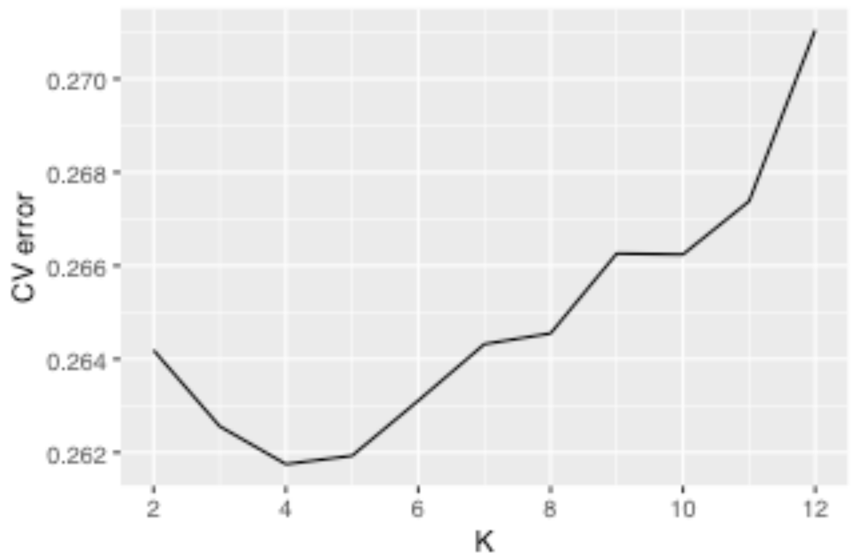

Figure S6. Admixture analysis cross validation (CV) errors**.** Plot of the admixture cross validation error verses the number of clusters (K) for the TrypanoGEN, 1000 Genomes and AGVP dataset.

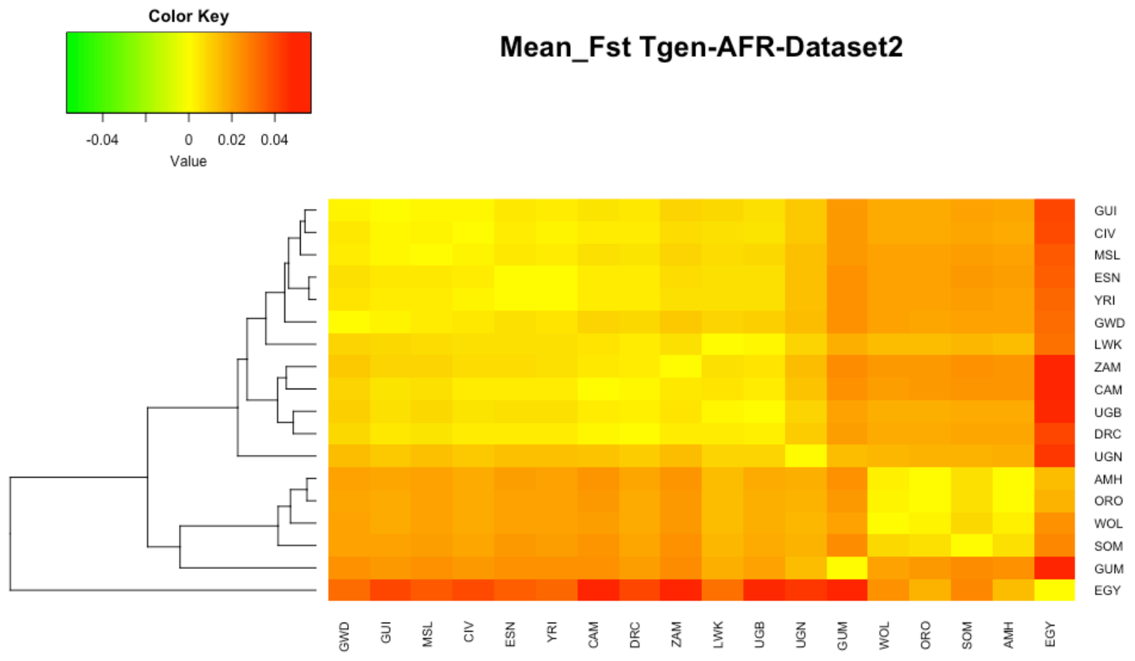

Figure S7. Heatmap of mean Fst between TrypanoGEN and 1000 genome African populations.

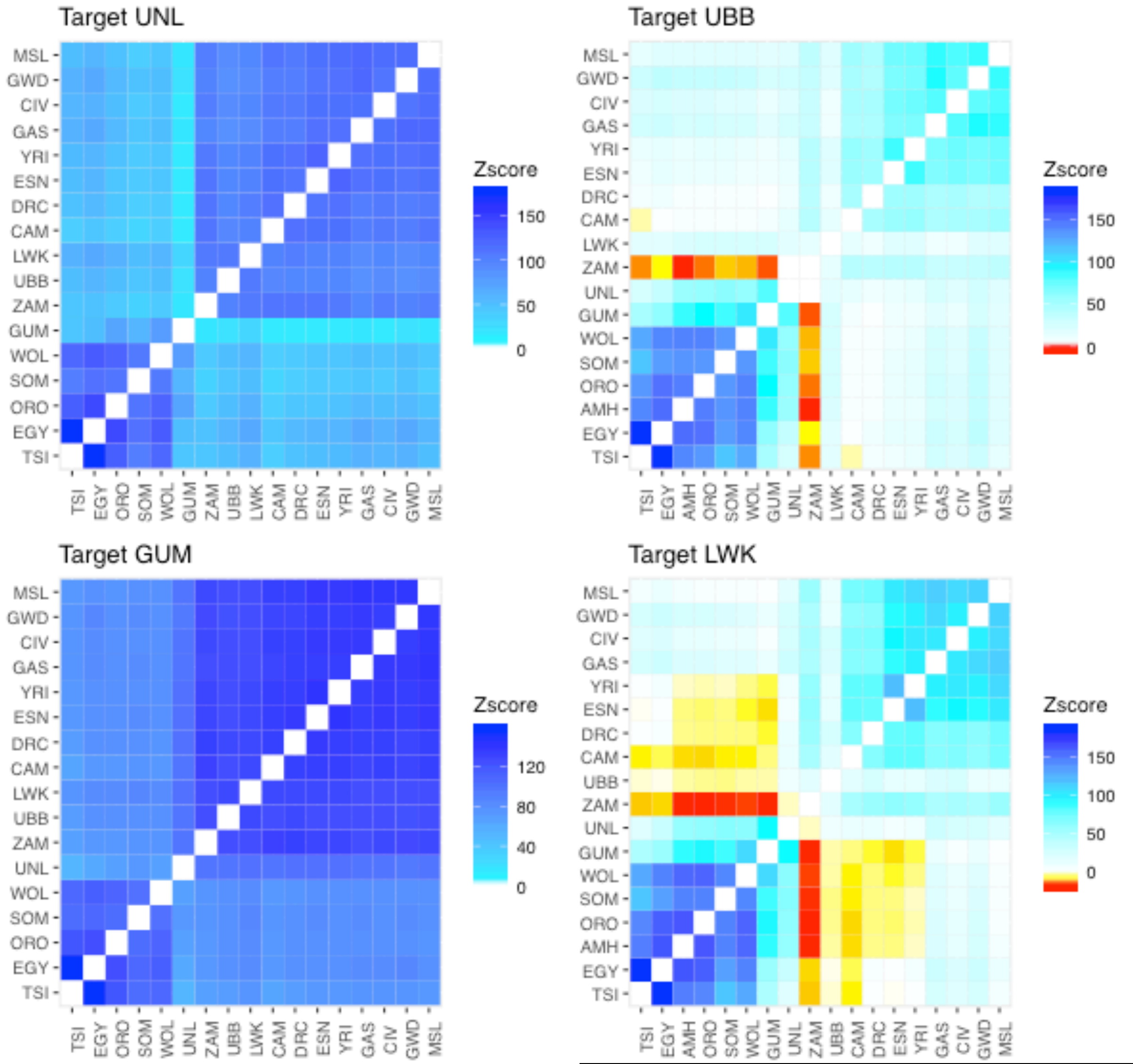

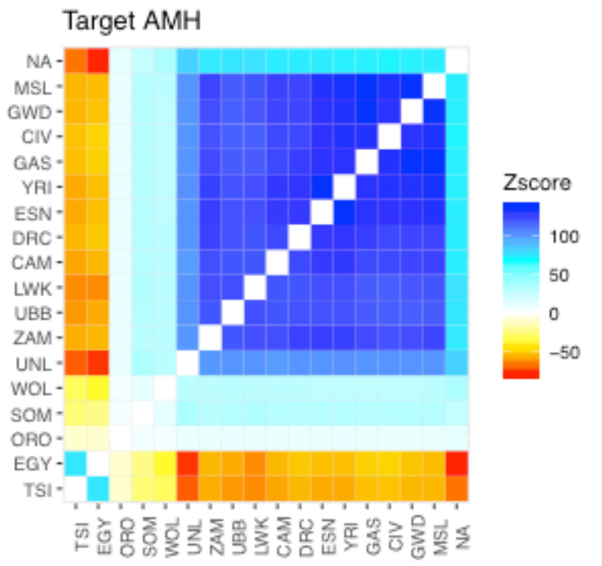

Figure S8. F3 tests to detect ancestral populations of the Lugbara (UNL), Basoga (UBB), Gumuz (GUN), Luhya (LWK) and Amhara (AMH) populations. Colour represents intensity of Z score for probability that a population contributes ancestral components to the target. Negative Z scores (yellow to red scale) are associated with increasingly strong evidence of a contribution and positive scores (cyan to blue) are associated with absence of evidence for a contribution.

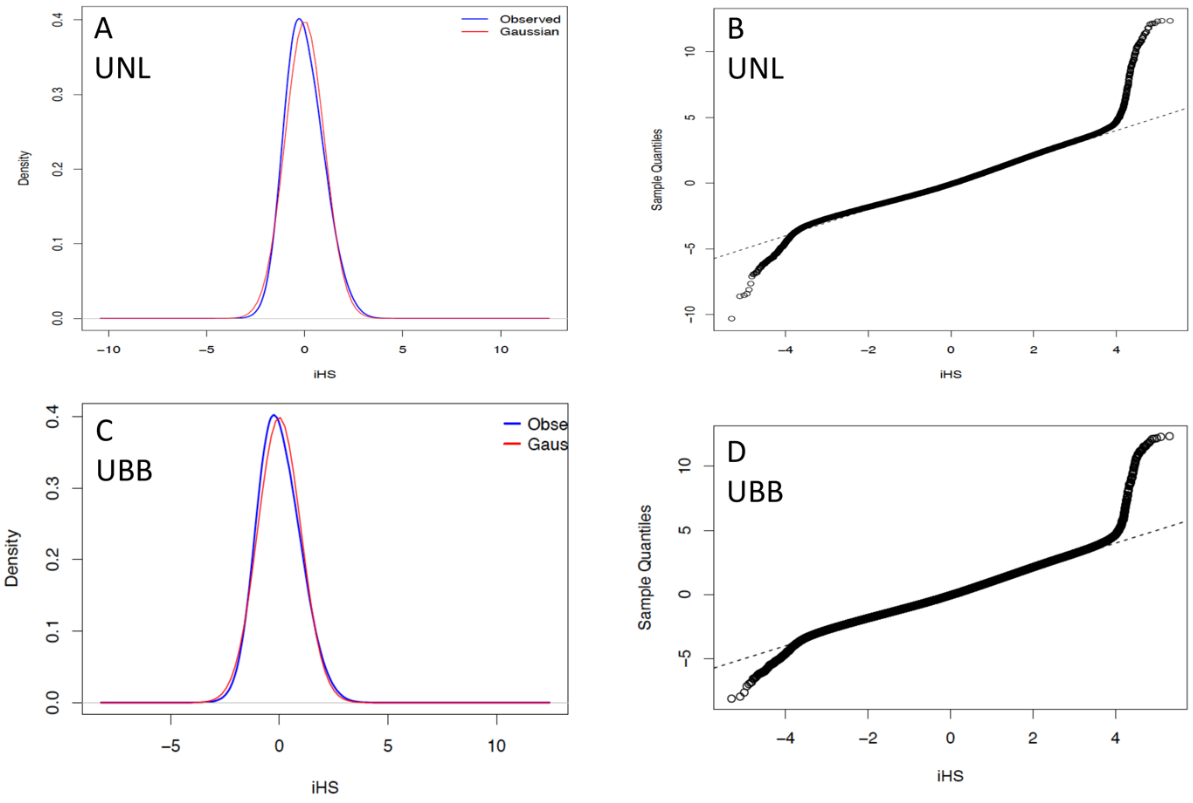

Figure S9. Analysis for signatures of selection in the Uganda Lugbara and Basoga populations. The UNL population **A.** genome wide density distribution histogram of the observed iHS values with respect to the Gaussian model and **B.** Q-Q plot of the genome wide iHS distribution for which the top iHS>3.0 were considered for selection analysis. **C** and **D** are the genome wide distribution and Q-Q plots respectively for the UBB population.

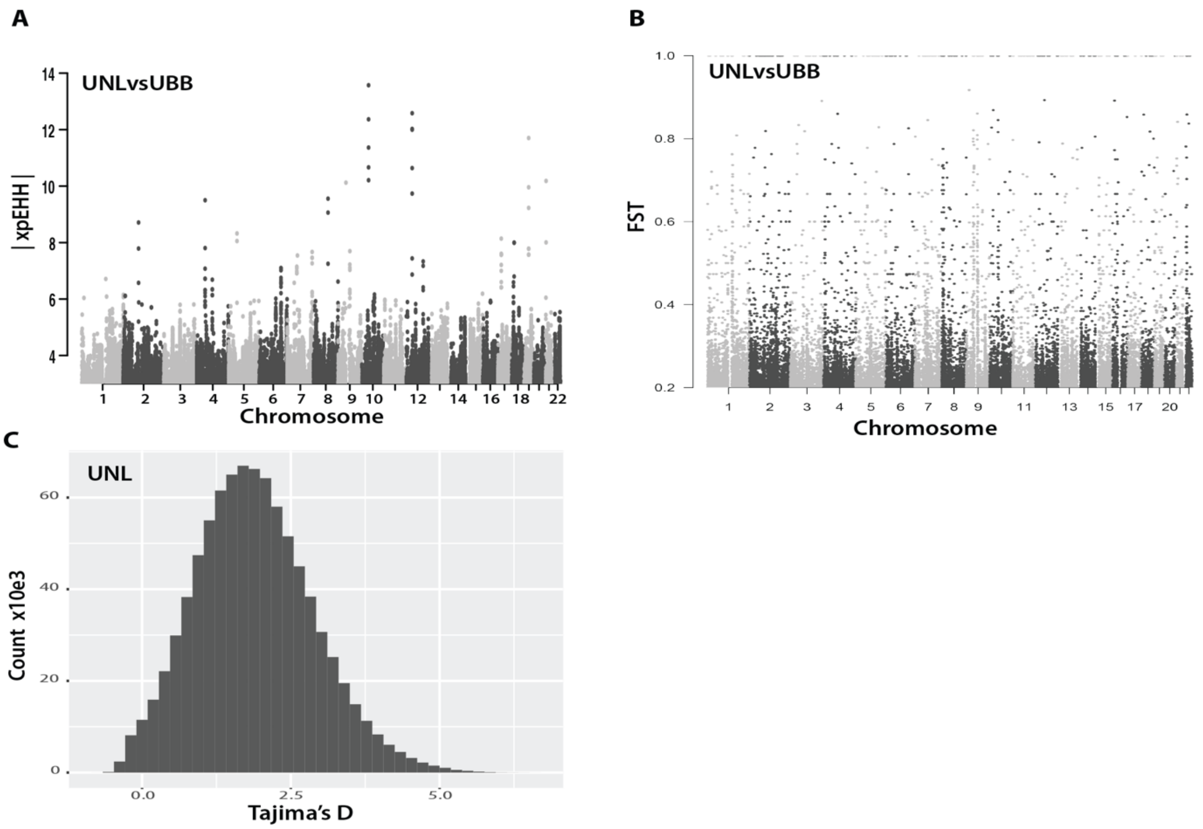

Figure S10. Genome wide signatures of selection that are differentiated between the UNL and UBB populations. **A.** The cross population extended haplotype analysis showing the xpEHH > 3.0. **B.** Genome wide distribution of FST>0.2 between the UNL and UBB populations. **C.** Normal distribution of the Tajima's D scores within the UNL population.

Supplemental tables

Country	Code	Ethnicity	Language Family and Major Branch	Ethnologue code	No. of Samples	Sample Source
Uganda	UNL	Lugbara	Nilo-Saharan, Central Sudanic	lgg	50	Tgen
	UBB	Basoga	Atlantic-Congo, Benue-Congo	xog	33	Tgen
Zambia	ZAM	Soli/Chikunda	Atlantic-Congo, Benue-Congo	sby; kdn	25	Tgen
		Tumbuka	Atlantic-Congo, Benue-Congo	tum	13	Tgen
		Bemba	Atlantic-Congo, Benue-Congo	bem	3	Tgen
Congo	DRC	Kimbala	Atlantic-Congo, Benue-Congo	mdp	20	Tgen
		Kingongo	Atlantic-Congo, Benue-Congo	noq	30	Tgen
Cameroon	CAM	Bamilike	Atlantic-Congo, Benue-Congo	fmp	6	Tgen
		Mundani	Atlantic-Congo, Benue-Congo	mnf	8	Tgen
		Ngoumba	Atlantic-Congo, Benue-Congo	nmg	12	Tgen
Ivory Coast	CIV	Baoule	Atlantic-Congo, Kwa	bci	11	Tgen
		Gouro	Mande	goa	21	Tgen
		More	Mande	Moa	12	Tgen
		Senoufo	Atlantic-Congo, Senufo	sef	4	Tgen
		Malinke	Mande	loi	1	Tgen
		Koyaka	Mande	kga	1	Tgen
Guinea	GAS	Soussou	Mande	sus	50	Tgen
Ethiopia	GUM	Gumuz	Nilo-Saharan, Kumuz	guk	24	AGVP
	AMH	Amharic	Afro-Asiatic, Semitic	amh	24	AGVP
	ORO	Oromo	Afro-Asiatic, Cushitic	hae	24	AGVP
	WOL	Wolaytta	Afro-Asiatic, Omotic	wal	24	AGVP
	SOM	Somali	Afro-Asiatic, Cushitic	som	24	AGVP
Egypt	EGY	Arabic	Afro-Asiatic, Semitic	arz	50	AGVP
Gambia	GWD	Mandika	Mande	mnk	50	1000G
Sierra Leone	MSL	Mende	Mande	men	50	1000G
Nigeria	ESN	Esan	Atlantic-Congo, Volta-Niger	ish	50	1000G
	YRI	Yoruba	Atlantic-Congo, Volta-Niger	yor	50	1000G
Kenya	LWK	Luhya	Atlantic-Congo, Benue-Congo	luy	50	1000G

Table S1. Ethno-linguistic classification of samples used for analysis. The Code is the abbreviation used for the group in the text and legends. Codes were assigned as follows: 1) name in original publication if previously published, 2) TrypanoGEN samples from a single country that clustered together on the MDS plot were designated as a population and assigned an abbreviation. Where there was a single linguistic group in a cluster from a country we referred to the samples from that cluster by a three letter code that consisted of, 1. Country/ geographical localisation, 2. Major Ethnic group and 3. Linguistic group. Eg GAS for Guinea, Niger-Congo-A, Soussou. For other clusters from a country where there were samples from multiple linguistic groups we referred to those samples by a three letter code for the country.

Cameroon (CAM)			Ivory Coast (CIV)			Republic of Congo (DRC)			Uganda, Basoga (UBB)			Uganda Lugbara (UNL)			Zambia (ZAM)			Guinea (GAS)		
Seq_ID	Ethnicity	Sex	Seq_ID	Ethnicity	Sex	Seq_ID	Ethnicity	Sex	Seq_ID	Ethnicity	Sex	Seq_ID	Ethnicity	Sex	Seq_ID	Ethnicity	Sex	Seq_ID	Ethnicity	Sex
CB12	Ngoumba	M	CIV_1	Gouro	F	DRC_1	Kingongo	M	UGB013C	Basoga	F	UGN005T	Lugbara	F	ZC08	Tumbuka	M	GUI_1	Soussou	F
CB14	Ngoumba	F	CIV_10	BaoulÈ	M	DRC_10	Kingongo	F	UGB015C	Basoga	M	UGN006C	Lugbara	F	ZC09	Tumbuka	M	GUI_10	Soussou	M
CB15	Ngoumba	M	CIV_11	MorÈ	M	DRC_11	Kingongo	F	UGB020C	Basoga	M	UGN044T	Lugbara	M	ZC10	Tumbuka	M	GUI_11	Soussou	F
CB16	Ngoumba	M	CIV_12	MorÈ	F	DRC_12	Kingongo	F	UGB022C	Basoga	F	UGN045C	Lugbara	M	ZC11	Tumbuka	F	GUI_12	Soussou	F
CB17	Ngoumba	M	CIV_13	MorÈ	M	DRC_13	Kingongo	F	UGB029C	Basoga	F	UGN046T	Lugbara	M	ZC13	Tumbuka	M	GUI_13	Soussou	F
CB24	Ngoumba	M	CIV_14	MorÈ	F	DRC_14	Kimbala	F	UGB038C	Basoga	F	UGN048T	Lugbara	M	ZC14	Tumbuka	M	GUI_14	Soussou	M
CB29	Ngoumba	M	CIV_15	Gouro	M	DRC_15	Kingongo	F	UGB039C	Basoga	F	UGN063T	Lugbara	F	ZC22	Tumbuka	F	GUI_15	Soussou	F
CB31	Ngoumba	M	CIV_16	Gouro	M	DRC_16	Kingongo	M	UGB040C	Basoga	F	UGN064C	Lugbara	F	ZC23	Tumbuka	M	GUI_16	Soussou	F
CB32	Ngoumba	F	CIV_17	Gouro	M	DRC_17	Kingongo	M	UGB044C	Basoga	M	UGN065C	Lugbara	F	ZC24	Tumbuka	M	GUI_18	Soussou	F
CB33	Ngoumba	F	CIV_18	Gouro	M	DRC_18	Kingongo	F	UGB046C	Basoga	F	UGN068C	Lugbara	M	ZC25	Tumbuka	M	GUI_2	Soussou	M
CB7	Ngoumba	F	CIV_19	MorÈ	F	DRC_19	Kingongo	M	UGB047C	Basoga	F	UGN069T	Lugbara	M	ZC26	Tumbuka	M	GUI_20	Soussou	M
CF24	Mundani	M	CIV_2	BaoulÈ	M	DRC_2	Kimbala	F	UGB049C	Basoga	F	UGN070C	Lugbara	M	ZC27	Tumbuka	F	GUI_21	Soussou	F
CF25	Mundani	M	CIV_20	BaoulÈ	F	DRC_20	Kingongo	F	UGB050C	Basoga	F	UGN071T	Lugbara	M	ZC28	Tumbuka	F	GUI_23	Soussou	M
CF26	Mundani	F	CIV_21	MorÈ	F	DRC_21	Kingongo	M	UGB051C	Basoga	F	UGN072C	Lugbara	M	ZM18	Bemba	M	GUI_24	Soussou	M
CF27	Mundani	M	CIV_22	Gouro	F	DRC_22	Kingongo	F	UGB056C	Basoga	F	UGN073C	Lugbara	M	ZM20	Bemba	M	GUI_25	Soussou	F
CF30	Mundani	M	CIV_23	Gouro	M	DRC_23	Kingongo	M	UGB059C	Basoga	F	UGN074T	Lugbara	F	ZM21	Bemba	F	GUI_26	Soussou	M
CF37	Mundani	M	CIV_24	Gouro	F	DRC_24	Kingongo	M	UGB062C	Basoga	M	UGN075C	Lugbara	F	ZR01	Soli/Chikunda	M	GUI_29	Soussou	M
CF46	Mundani	F	CIV_25	Gouro	M	DRC_25	Kingongo	M	UGB066C	Basoga	M	UGN076T	Lugbara	F	ZR02	Soli/Chikunda	M	GUI_3	Soussou	M
CF49	Mundani	F	CIV_26	Gouro	M	DRC_26	Kingongo	F	UGB067C	Basoga	M	UGN077C	Lugbara	F	ZR04	Soli/Chikunda	M	GUI_30	Soussou	M
CP17	Bamilike	F	CIV_27	Gouro	M	DRC_27	Kimbala	M	UGB068C	Basoga	F	UGN079C	Lugbara	F	ZR05	Soli/Chikunda	M	GUI_31	Soussou	M
CP28	Ngoumba	M	CIV_28	Gouro	M	DRC_28	Kingongo	F	UGB071C	Basoga	M	UGN080C	Lugbara	F	ZR06	Soli/Chikunda	M	GUI_32	Soussou	F
CP36	Bamilike	F	CIV_29	Gouro	M	DRC_29	Kingongo	M	UGB072C	Basoga	F	UGN082C	Lugbara	M	ZR07	Soli/Chikunda	M	GUI_33	Soussou	F
CP38	Bamilike	M	CIV_3	BaoulÈ	M	DRC_3	Kimbala	F	UGB073C	Basoga	F	UGN088C	Lugbara	M	ZR29	Soli/Chikunda	F	GUI_34	Soussou	M
CP40	Bamilike	F	CIV_30	MorÈ	M	DRC_30	Kimbala	F	UGB074C	Basoga	F	UGN090C	Lugbara	M	ZR30	Soli/Chikunda	F	GUI_35	Soussou	F
CP48	Bamilike	F	CIV_35	BaoulÈ	F	DRC_31	Kimbala	F	UGB077C	Basoga	F	UGN091C	Lugbara	M	ZR31	Soli/Chikunda	F	GUI_36	Soussou	F
CP9	Bamilike	M	CIV_36	Koyaka	F	DRC_32	Kimbala	F	UGB079C	Basoga	F	UGN092C	Lugbara	M	ZR32	Soli/Chikunda	M	GUI_37	Soussou	M

	CIV_37	BaoulÈ	F	DRC_33	Kimbala	M	UGB105C	Basoga	F	UGN093C	Lugbara	M	ZR33	Soli/Chikunda	F	GUI_38	Soussou	F
	CIV_38	BaoulÈ	M	DRC_34	Kingongo	M	UGB350C	Basoga	F	UGN098C	Lugbara	M	ZR34	Soli/Chikunda	M	GUI_39	Soussou	F
	CIV_39	SÈnoufo	M	DRC_35	Kingongo	M	UGB351C	Basoga	M	UGN099C	Lugbara	F	ZR35	Soli/Chikunda	F	GUI_4	Soussou	M
	CIV_4	Gouro	M	DRC_36	Kingongo	F	UGB369C	Basoga	M	UGN100C	Lugbara	F	ZR36	Soli/Chikunda	M	GUI_40	Soussou	M
	CIV_40	Gouro	F	DRC_37	Kingongo	F	UGB371C	Basoga	F	UGN105C	Lugbara	M	ZR37	Soli/Chikunda	M	GUI_41	Soussou	F
	CIV_41	More	F	DRC_38	Kingongo	M	UGB383C	Basoga	M	UGN106C	Lugbara	M	ZR38	Soli/Chikunda	M	GUI_42	Soussou	F
	CIV_42	BaoulÈ	M	DRC_39	Kimbala	M	UGB386C	Basoga	M	UGN107C	Lugbara	M	ZR39	Soli/Chikunda	F	GUI_43	Soussou	M
	CIV_43	MorÈ	M	DRC_4	Kimbala	F				UGN109C	Lugbara	M	ZR40	Soli/Chikunda	F	GUI_44	Soussou	F
	CIV_44	BaoulÈ	F	DRC_40	Kimbala	M				UGN113T	Lugbara	F	ZR41	Soli/Chikunda	M	GUI_45	Soussou	M
	CIV_45	Gouro	M	DRC_41	Kimbala	F				UGN114C	Lugbara	F	ZR42	Soli/Chikunda	M	GUI_46	Soussou	F
	CIV_48	BaoulÈ	M	DRC_42	Kimbala	M				UGN115C	Lugbara	F	ZR43	Soli/Chikunda	F	GUI_47	Soussou	M
	CIV_49	SÈnoufo	F	DRC_43	Kimbala	F				UGN124T	Lugbara	M	ZR44	Soli/Chikunda	F	GUI_48	Soussou	M
	CIV_5	Gouro	M	DRC_44	Kimbala	M				UGN125T	Lugbara	M	ZR46	Soli/Chikunda	M	GUI_49	Soussou	F
	CIV_50	Gouro	M	DRC_45	Kingongo	M				UGN127C	Lugbara	F	ZR47	Soli/Chikunda	F	GUI_5	Soussou	M
	CIV_51	Gouro	F	DRC_46	Kimbala	F				UGN134T	Lugbara	M	ZR49	Soli/Chikunda	M	GUI_50	Soussou	F
	CIV_52	ND	M	DRC_47	Kimbala	M				UGN136T	Lugbara	F				GUI_51	Soussou	F
	CIV_53	Gouro	M	DRC_48	Kingongo	F				UGN137C	Lugbara	F				GUI_52	Soussou	F
	CIV_54	SÈnoufo	M	DRC_49	Kingongo	M				UGN140T	Lugbara	F				GUI_53	Soussou	F
	CIV_55	Gouro	M	DRC_5	Kimbala	M				UGN142T	Lugbara	F				GUI_54	Soussou	F
	CIV_56	MorÈ	M	DRC_50	Kingongo	F				UGN144T	Lugbara	M				GUI_55	Soussou	M
	CIV_6	Gouro	M	DRC_6	Kimbala	F				UGN148T	Lugbara	F				GUI_6	Soussou	M
	CIV_7	MalinkÈ	M	DRC_7	Kingongo	F				UGN153T	Lugbara	M				GUI_7	Soussou	F
	CIV_8	MorÈ	F	DRC_8	Kingongo	F				UGN157T	Lugbara	F				GUI_8	Soussou	F
	CIV_9	BaoulÈ	F	DRC_9	Kimbala	F				UGN185C	Lugbara	M				GUI_9	Soussou	M

Table S2. Sample sequence identifier**,** ethnicity and sex of each participant whose DNA was sequenced

Filter	Count Loci
Total Loci Discovered	38,963,563
Minor allele count < 3	16,840,310
MAF < 0.01	4,764,259
pHWE < 0.001	1,106,883
Missing genotype data > 0.1	306,271
Total loci passing QC	15,945,840

Table S3 Number of loci discovered and number removed by each filter**.** Note that the number of loci removed by a given filter will depend on the order in which filters are applied. We have listed filters in order of effect size.

Variants	ZAM*	UBB	CIV	CAM*	DRC	GAS	UNL
**Total variants**	**21,346,657**	**20,244,883**	**21,546,091**	**20,154,485**	**22,100,090**	**21,703,906**	**21,891,961**
**Known_variants**	18,348,704	17,773,731	18,895,407	17,466,021	19,245,113	18,948,607	18,145,718
**Novel_variants**	2,997,953	2,471,152	2,650,684	2,688,464	2,854,977	2,755,299	3,746,243
**Known_low**	10,705	10,316	11,046	10,187	11,337	11,207	10,626
**Known_modifier**	18,242,755	17,674,567	18,788,695	17,367,207	19,134,729	18,839,449	18,040,733
**Known_moderate**	92,096	85,916	92,517	85,651	95,710	94,746	91,028
**Known_high**	3,147	2,931	3,148	2,975	3,336	3,204	2,981
**Novel_low**	339	323	347	312	285	330	702
**Novel_modifier**	2,989,075	2,464,046	2,642,851	2,680,549	2,847,670	2,747,181	3,732,449
**Novel_moderate**	7,996	6,357	6,933	7,073	6,569	7,271	12,290
**Novel_high**	543	426	553	530	453	517	802

Table S4. The number of variants obtained from the mapping and variant calling pipeline for each population. All samples were sequenced at 10X coverage except those from *Zambia and *Cameroon, which were at 30X coverage. The variants that were annotated with a dbSNP rsID were termed ‘Known_variants’ whereas those without were termed ‘Novel_variants’. The impact of the genomic variant as annotated by SnpEff were classified as ‘Low’, ‘Modifier’, ‘Moderate’ or ‘High’ based on their effect on transcription and/or translation. The Low impact variant features result in changes/mutations in the start & stop codons, splice site regions; Modifier variants affected mainly intergenic regions; Moderate impact variants features result in codon change, 3’ & 5’ UTR truncation exon loss, splice site branch region for U12 splicing machinery; High impact variant features occur in and affect chromosome deletion, exon deletion, frame shift, rare amino acid, splice site acceptor and donor, loss or gain of stop & start codons. Details of the classification are in table S6.

	AMH	CAM	CIV	DRC	EGY	ESN	GAS	GUM	GWD	LWK	MSL	ORO	SOM	UBB	UNL	WOL	YRI	ZAM
**AMH**	0.00000	0.04689	0.04462	0.04450	0.01703	0.04737	0.04450	0.04232	0.04470	0.03643	0.04676	0.00047	0.00981	0.03977	0.03659	0.00471	0.04624	0.04941
**CAM**	0.04689	0.00000	0.00585	0.00265	0.08740	0.00490	0.00732	0.03969	0.00908	0.00593	0.00680	0.04349	0.04284	0.00521	0.01585	0.03909	0.00454	0.00618
**CIV**	0.04462	0.00585	0.00000	0.00473	0.08127	0.00508	0.00242	0.04155	0.00604	0.00913	0.00366	0.04173	0.04163	0.00709	0.01734	0.03841	0.00375	0.01023
**DRC**	0.04450	0.00265	0.00473	0.00000	0.08183	0.00541	0.00644	0.03993	0.00949	0.00582	0.00769	0.04151	0.04112	0.00380	0.01581	0.03783	0.00497	0.00599
**EGY**	0.01703	0.08740	0.08127	0.08183	0.00000	0.08210	0.08120	0.08816	0.07802	0.07190	0.08220	0.01992	0.03472	0.07931	0.07969	0.03098	0.08066	0.08793
**ESN**	0.04737	0.00490	0.00508	0.00541	0.08210	0.00000	0.00716	0.04328	0.00710	0.00750	0.00513	0.04448	0.04397	0.00841	0.01980	0.04116	0.00080	0.00903
**GAS**	0.04450	0.00732	0.00242	0.00644	0.08120	0.00716	0.00000	0.04155	0.00343	0.01024	0.00278	0.04159	0.04154	0.00844	0.01790	0.03816	0.00581	0.01155
**GUM**	0.04232	0.03969	0.04155	0.03993	0.08816	0.04328	0.04155	0.00000	0.04254	0.03244	0.04236	0.03827	0.04041	0.03446	0.02525	0.03203	0.04235	0.04339
**GWD**	0.04470	0.00908	0.00604	0.00949	0.07802	0.00710	0.00343	0.04254	0.00000	0.01032	0.00360	0.04191	0.04166	0.01136	0.02030	0.03902	0.00589	0.01293
**LWK**	0.03643	0.00593	0.00913	0.00582	0.07190	0.00750	0.01024	0.03244	0.01032	0.00000	0.00912	0.03361	0.03336	0.00210	0.01258	0.03016	0.00690	0.00773
**MSL**	0.04676	0.00680	0.00366	0.00769	0.08220	0.00513	0.00278	0.04236	0.00360	0.00912	0.00000	0.04384	0.04350	0.00990	0.01993	0.04017	0.00395	0.01096
**ORO**	0.00047	0.04349	0.04173	0.04151	0.01992	0.04448	0.04159	0.03827	0.04191	0.03361	0.04384	0.00000	0.00885	0.03676	0.03335	0.00354	0.04344	0.04621
**SOM**	0.00981	0.04284	0.04163	0.04112	0.03472	0.04397	0.04154	0.04041	0.04166	0.03336	0.04350	0.00885	0.00000	0.03644	0.03191	0.01122	0.04289	0.04566
**UBB**	0.03977	0.00521	0.00709	0.00380	0.07931	0.00841	0.00844	0.03446	0.01136	0.00210	0.00990	0.03676	0.03644	0.00000	0.01191	0.03279	0.00778	0.00723
**UNL**	0.03659	0.01585	0.01734	0.01581	0.07969	0.01980	0.01790	0.02525	0.02030	0.01258	0.01993	0.03335	0.03191	0.01191	0.00000	0.02929	0.01890	0.02013
**WOL**	0.00471	0.03909	0.03841	0.03783	0.03098	0.04116	0.03816	0.03203	0.03902	0.03016	0.04017	0.00354	0.01122	0.03279	0.02929	0.00000	0.04014	0.04206
**YRI**	0.04624	0.00454	0.00375	0.00497	0.08066	0.00080	0.00581	0.04235	0.00589	0.00690	0.00395	0.04344	0.04289	0.00778	0.01890	0.04014	0.00000	0.00861
**ZAM**	0.04941	0.00618	0.01023	0.00599	0.08793	0.00903	0.01155	0.04339	0.01293	0.00773	0.01096	0.04621	0.04566	0.00723	0.02013	0.04206	0.00861	0.00000

Table S5A. Matrix of the weighted F_{ST} statistic values between the TrypanoGEN, 1000 genomes and AGVP data sets

		AFR	AFR	AFR	AFR	AFR	AFR	AFR	AMR	AMR	AMR	AMR	EAS	EAS	EAS	EAS	EAS	EUR	EUR	EUR	EUR	EUR	SAS	SAS	SAS	SAS	SAS
Super	pop	ACB	ASW	ESN	GWD	LWK	MSL	YRI	CLM	MXL	PEL	PUR	CDX	CHB	CHS	JPT	KHV	CEU	FIN	GBR	IBS	TSI	BEB	GIH	ITU	PJL	STU
AFR	ACB		0.002	0.003	0.006	0.006	0.004	0.002	0.082	0.095	0.127	0.071	0.129	0.130	0.131	0.131	0.127	0.103	0.107	0.103	0.099	0.101	0.094	0.097	0.096	0.093	0.095
AFR	ASW	0.002		0.009	0.010	0.009	0.010	0.008	0.064	0.078	0.110	0.053	0.116	0.116	0.117	0.117	0.113	0.085	0.088	0.085	0.081	0.083	0.077	0.080	0.079	0.076	0.079
AFR	ESN	0.003	0.009		0.007	0.008	0.005	0.001	0.106	0.119	0.149	0.094	0.150	0.151	0.152	0.152	0.148	0.130	0.133	0.130	0.126	0.127	0.116	0.120	0.119	0.117	0.118
AFR	GWD	0.006	0.010	0.007		0.011	0.004	0.006	0.101	0.114	0.143	0.089	0.145	0.146	0.147	0.147	0.143	0.124	0.127	0.124	0.120	0.121	0.112	0.115	0.114	0.112	0.113
AFR	LWK	0.006	0.009	0.008	0.011		0.009	0.007	0.096	0.109	0.139	0.084	0.140	0.141	0.142	0.142	0.138	0.119	0.122	0.119	0.115	0.116	0.106	0.110	0.109	0.106	0.108
AFR	MSL	0.004	0.010	0.005	0.004	0.009		0.004	0.106	0.119	0.149	0.094	0.151	0.152	0.154	0.153	0.149	0.130	0.134	0.130	0.126	0.128	0.117	0.121	0.119	0.117	0.119
AFR	YRI	0.002	0.008	0.001	0.006	0.007	0.004		0.104	0.117	0.146	0.092	0.148	0.149	0.150	0.150	0.146	0.128	0.131	0.128	0.124	0.125	0.115	0.119	0.117	0.115	0.117
AMR	CLM	0.082	0.064	0.106	0.101	0.096	0.106	0.104		0.009	0.035	0.005	0.068	0.064	0.067	0.066	0.064	0.014	0.017	0.014	0.013	0.014	0.026	0.026	0.029	0.022	0.029
AMR	MXL	0.095	0.078	0.119	0.114	0.109	0.119	0.117	0.009		0.016	0.017	0.064	0.058	0.061	0.059	0.060	0.032	0.033	0.033	0.032	0.033	0.033	0.035	0.037	0.031	0.037
AMR	PEL	0.127	0.110	0.149	0.143	0.139	0.149	0.146	0.035	0.016		0.051	0.079	0.072	0.075	0.073	0.075	0.077	0.074	0.077	0.078	0.078	0.063	0.068	0.068	0.065	0.068
AMR	PUR	0.071	0.053	0.094	0.089	0.084	0.094	0.092	0.005	0.017	0.051		0.073	0.070	0.072	0.072	0.069	0.010	0.015	0.010	0.008	0.009	0.026	0.025	0.028	0.021	0.028
EAS	CDX	0.129	0.116	0.150	0.145	0.140	0.151	0.148	0.068	0.064	0.079	0.073		0.008	0.005	0.016	0.002	0.094	0.089	0.094	0.093	0.093	0.050	0.066	0.062	0.063	0.060
EAS	CHB	0.130	0.116	0.151	0.146	0.141	0.152	0.149	0.064	0.058	0.072	0.070	0.008		0.001	0.007	0.006	0.091	0.085	0.092	0.091	0.091	0.049	0.064	0.060	0.062	0.059
EAS	CHS	0.131	0.117	0.152	0.147	0.142	0.154	0.150	0.067	0.061	0.075	0.072	0.005	0.001		0.008	0.003	0.093	0.088	0.094	0.093	0.093	0.050	0.065	0.062	0.063	0.060
EAS	JPT	0.131	0.117	0.152	0.147	0.142	0.153	0.150	0.066	0.059	0.073	0.072	0.016	0.007	0.008		0.013	0.093	0.087	0.093	0.092	0.093	0.051	0.065	0.062	0.063	0.060
EAS	KHV	0.127	0.113	0.148	0.143	0.138	0.149	0.146	0.064	0.060	0.075	0.069	0.002	0.006	0.003	0.013		0.090	0.085	0.090	0.089	0.089	0.047	0.062	0.058	0.059	0.056
EUR	CEU	0.103	0.085	0.130	0.124	0.119	0.130	0.128	0.014	0.032	0.077	0.010	0.094	0.091	0.093	0.093	0.090		0.006	0.000	0.002	0.003	0.035	0.031	0.036	0.025	0.036
EUR	FIN	0.107	0.088	0.133	0.127	0.122	0.134	0.131	0.017	0.033	0.074	0.015	0.089	0.085	0.088	0.087	0.085	0.006		0.007	0.010	0.011	0.035	0.032	0.037	0.027	0.037
EUR	GBR	0.103	0.085	0.130	0.124	0.119	0.130	0.128	0.014	0.033	0.077	0.010	0.094	0.092	0.094	0.093	0.090	0.000	0.007		0.002	0.004	0.035	0.031	0.036	0.026	0.037
EUR	IBS	0.099	0.081	0.126	0.120	0.115	0.126	0.124	0.013	0.032	0.078	0.008	0.093	0.091	0.093	0.092	0.089	0.002	0.010	0.002		0.002	0.035	0.031	0.036	0.026	0.036
EUR	TSI	0.101	0.083	0.127	0.121	0.116	0.128	0.125	0.014	0.033	0.078	0.009	0.093	0.091	0.093	0.093	0.089	0.003	0.011	0.004	0.002		0.034	0.030	0.034	0.025	0.035
SAS	BEB	0.094	0.077	0.116	0.112	0.106	0.117	0.115	0.026	0.033	0.063	0.026	0.050	0.049	0.050	0.051	0.047	0.035	0.035	0.035	0.035	0.034		0.004	0.002	0.003	0.002
SAS	GIH	0.097	0.080	0.120	0.115	0.110	0.121	0.119	0.026	0.035	0.068	0.025	0.066	0.064	0.065	0.065	0.062	0.031	0.032	0.031	0.031	0.030	0.004		0.004	0.004	0.004
SAS	ITU	0.096	0.079	0.119	0.114	0.109	0.119	0.117	0.029	0.037	0.068	0.028	0.062	0.060	0.062	0.062	0.058	0.036	0.037	0.036	0.036	0.034	0.002	0.004		0.003	0.001
SAS	PJL	0.093	0.076	0.117	0.112	0.106	0.117	0.115	0.022	0.031	0.065	0.021	0.063	0.062	0.063	0.063	0.059	0.025	0.027	0.026	0.026	0.025	0.003	0.004	0.003		0.003
SAS	STU	0.095	0.079	0.118	0.113	0.108	0.119	0.117	0.029	0.037	0.068	0.028	0.060	0.059	0.060	0.060	0.056	0.036	0.037	0.037	0.036	0.035	0.002	0.004	0.001	0.003	

Table S5B. Matrix of the weighted F_{ST} statistic values between the global 1000 genomes populations for comparison with African distances. The comparisons within super populations are highlighted in yellow and summarised in Table S5C below

	Max Fst	Mean Fst
**Africa**	0.0105	0.0063
**Americas**	0.0509	0.0222
**East Asia**	0.0160	0.0069
**West Eurasia**	0.0112	0.0047
**South Asia**	0.0043	0.0032

Table S5C. Summary of weighted F_{ST} statistic values within super populations of 1000 Genomes samples. Note the high values for F_{ST} within the Americas, which are presumably due to high levels of admixture. Although the values for Africa are similar to the values for the major Eurasian groups it should be remembered that the 1000 Genomes project only included samples from the Niger-Congo linguistic group and the other major linguistic groups were not represented.

Supplemental Excel Spreadsheets

Table S6. snpEff classification of effect of SNP and its impact.

Table S7. Genome wide distribution of extreme signatures of selection in the UNL. Ensembl annotations of the coding and non-coding regions of the genome harbouring extreme iHS scores (positive iHS > +3.0, negative iHS < -3).

Table S8. Protein coding genes under positive selection in the UNL. A list of unique genes having extreme iHS scores (> +3.0) including those that intersect with the UBB population.

Table S9. Top hits of significant genes in UNL. Top hits of significant genes in UNL. Genes in the 1% of 100kb bins with highest frequencies of SNP with absolute iHS > 2.

Table S10. **Top hits of significant genes unique to the UNL**. Genes in top 1% of 100kb bins from table S9 that are only present in the UNL and not found in the UBB population.

Table S11. **Top hits of significant genes highly differentiated between the UNL and UBB**. Genes were ranked individually on the parameters of xpEHH [UNL-UBB], high Fst [UNL-UBB], and Tajima's D [UNL], and then a combined rank was obtained by summation of the individual ranks.
